# A heterotypic assembly mechanism regulates CHIP E3 ligase activity

**DOI:** 10.15252/embj.2021109566

**Published:** 2022-06-28

**Authors:** Aniruddha Das, Pankaj Thapa, Ulises Santiago, Nilesh Shanmugam, Katarzyna Banasiak, Katarzyna Dąbrowska, Hendrik Nolte, Natalia A Szulc, Rose M Gathungu, Dominik Cysewski, Marcus Krüger, Michał Dadlez, Marcin Nowotny, Carlos J Camacho, Thorsten Hoppe, Wojciech Pokrzywa

**Affiliations:** ^1^ Laboratory of Protein Metabolism International Institute of Molecular and Cell Biology in Warsaw Warsaw Poland; ^2^ Department of Computational and Systems Biology University of Pittsburgh Pittsburgh PA USA; ^3^ Institute of Biochemistry and Biophysics PAS Warsaw Poland; ^4^ Institute for Genetics and Cologne Excellence Cluster on Cellular Stress Responses in Aging‐Associated Diseases (CECAD) University of Cologne Cologne Germany; ^5^ Metabolomics Core Facility EMBL Heidelberg Germany; ^6^ Center for Molecular Medicine (CMMC), Faculty of Medicine University Hospital of Cologne Cologne Germany; ^7^ Laboratory of Protein Structure International Institute of Molecular and Cell Biology in Warsaw Warsaw Poland; ^8^ Present address: Max‐Planck‐Institute for Biology of Ageing Cologne Germany

**Keywords:** *C. elegans*, CHIP/STUB1/CHN‐1, metabolism, ubiquitin ligase, UFD‐2, Post-translational Modifications & Proteolysis, Translation & Protein Quality

## Abstract

CHIP (C‐terminus of Hsc70‐interacting protein) and its worm ortholog CHN‐1 are E3 ubiquitin ligases that link the chaperone system with the ubiquitin‐proteasome system (UPS). CHN‐1 can cooperate with UFD‐2, another E3 ligase, to accelerate ubiquitin chain formation; however, the basis for the high processivity of this E3s set has remained obscure. Here, we studied the molecular mechanism and function of the CHN‐1–UFD‐2 complex in *Caenorhabditis elegans*. Our data show that UFD‐2 binding promotes the cooperation between CHN‐1 and ubiquitin‐conjugating E2 enzymes by stabilizing the CHN‐1 U‐box dimer. However, HSP70/HSP‐1 chaperone outcompetes UFD‐2 for CHN‐1 binding, thereby promoting a shift to the autoinhibited CHN‐1 state by acting on a conserved residue in its U‐box domain. The interaction with UFD‐2 enables CHN‐1 to efficiently ubiquitylate and regulate *S*‐adenosylhomocysteinase (AHCY‐1), a key enzyme in the *S*‐adenosylmethionine (SAM) regeneration cycle, which is essential for SAM‐dependent methylation. Our results define the molecular mechanism underlying the synergistic cooperation of CHN‐1 and UFD‐2 in substrate ubiquitylation.

## Graphical abstract

CHIP/CHN‐1 ubiquitylation processivity is controlled by differential association with UFD‐2 E3 ligase and HSP70/HSP‐1 chaperone, and is involved in *C. elegans* lipid metabolism.

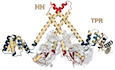

## Introduction

The ubiquitin‐proteasome system (UPS) comprises a well‐studied enzymatic cascade that transfers the small protein ubiquitin (Ub) onto a protein substrate (Kerscher *et al*, 2006). The last step in the UPS enzymatic cascade is mediated by ubiquitin ligases (E3s), the largest and most diverse group of proteins within the UPS, which are responsible for substrate selection and specificity (Komander, 2009; Buetow & Huang, 2016). Mechanistically, two classes of E3 enzymes are commonly found. HECT (homologous to E6AP C‐terminus) E3s form an intermediate thioester bond with ubiquitin before catalyzing substrate ubiquitylation. By contrast, RING (Really Interesting New Gene)/U‐box E3s form molecular scaffolds that bring E2‐Ub and the target protein into proximity, thereby facilitating direct Ub transfer to the latter (Wenzel *et al*, 2011; Riley *et al*, 2013; Buetow & Huang, 2016). In some instances, other proteins, called ubiquitin chain elongation factors, or E4s, may be required to achieve efficient poly‐ubiquitylation of substrates. The first E4 described was yeast Ufd2p (Richly *et al*, 2005), a U‐box domain‐containing protein that engages Ub via its N‐terminal region, thus enhancing Ub chain elongation on a pre‐ubiquitylated substrate (Koegl *et al*, 1999; Hatakeyama *et al*, 2001; Buetow & Huang, 2016). Although higher eukaryotes, including humans, have an ortholog of yeast Ufd2p, its Ub‐interacting motif has little sequence homology (Hänzelmann *et al*, 2010; Liu *et al*, 2017), suggesting that the function of UFD‐2 as an E4 is not evolutionarily conserved.

Early *Caenorhabditis elegans* studies showed that UFD‐2 interacts directly with CHN‐1 (the nematode ortholog of mammalian CHIP) to form an E3‐E4 complex that can efficiently oligo‐ubiquitylate the myosin chaperone UNC‐45 (Hoppe *et al*, 2004). CHIP (C‐terminus of Hsc70‐interacting protein), initially identified as a tetratricopeptide repeat (TPR) protein that interacts with heat shock proteins (Ballinger *et al*, 1999), is a U‐box E3 ubiquitin ligase that mediates ubiquitylation of chaperone client proteins, promoting their degradation (Murata *et al*, 2001; Paul & Ghosh, 2014; Joshi *et al*, 2016). In contrast to the model proposed based on these early findings, more recent studies have revealed that UFD‐2 acts as a true E3 ligase that poly‐ubiquitylates UNC‐45 independent of CHN‐1, suggesting that both UFD‐2 and CHN‐1 act as E3s in the same or an overlapping substrate space (Hellerschmied *et al*, 2018). A recent study aimed at identifying substrates of human CHIP and the human UFD‐2 ortholog UBE4B supports the possibility of shared substrate scope (Bhuripanyo *et al*, 2018). Nevertheless, despite the key role of CHIP/CHN‐1 in protein quality control networks, little is known about the regulation of its activity and its interaction with UFD‐2, as well as the functional role of this E3 pair.

To address these questions, we combined *in vitro* and *in vivo* assays with computational approaches and lipidomic and proteomic studies in *C. elegans* to uncover the mechanism that controls CHN‐1 activity. The crystal structure of murine CHIP bound to the C‐terminal decapeptide of the HSP90 chaperone via the TPR domain revealed an asymmetric dimerization in which the two CHIP protomers adopt a “closed” conformation that restricts E2 access to one of the U‐box domains, and thus E3 activity (Zhang *et al*, 2005). Subsequent molecular modeling of mouse CHIP indicates dynamics between symmetric and asymmetric autoinhibited dimers, which can be regulated by binding proteins (Ye *et al*, 2017). Our results show that although metazoan UFD‐2 lacks E4 activity, it acts as a pre‐conditioning factor to influence the conformational flexibility of CHN‐1, thus boosting its processivity. Mechanistically, UFD‐2 binding to the TPR domain stabilizes the open conformation of CHN‐1, allowing the U‐box dimer to discharge more Ub‐conjugating enzymes (E2) in a single ubiquitylation cycle. We also demonstrated that the heat‐shock protein HSP70/HSP‐1 interacts with the TPR and U‐box domains of CHN‐1 to stabilize the closed/auto‐inhibitory state of the CHN‐1 dimer, thus limiting its interaction with E2 and UFD‐2. Furthermore, we identified potential substrates for the CHN‐1–UFD‐2 pair, including *S*‐adenosylhomocysteinase (AHCY‐1), a metabolic enzyme previously not known to be a client of heat‐shock proteins. However, the UFD‐2‐dependent increase in CHN‐1 processivity and consequent CHN‐1 auto‐ubiquitylation (auto‐Ub) also lead to CHN‐1 turnover, thereby reducing organismal proteostasis capacity. Collectively, our results indicate an interplay between chaperones and UFD‐2 in modulating CHN‐1 activity. This processivity‐switching behavior of CHN‐1 has important implications for its roles in regulating proteostasis, metabolism, and potentially other cellular processes.

## Results

### UFD‐2 promotes CHN‐1 processivity and its cooperation with E2s

**Figure 1:**
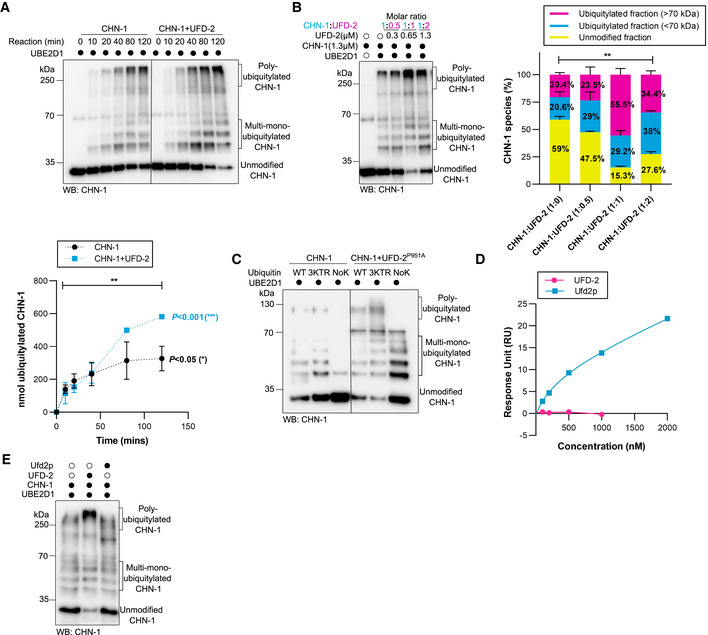
UFD‐2 activates CHN‐1 ATime‐dependent (0, 10, 20, 40, 80, and 120 min) CHN‐1 auto‐Ub was performed as indicated using Ub^WT^ and UBE2D1 E2. Protein samples were resolved via SDS–PAGE and immunoblotted with anti‐CHN‐1 antibodies. Below, a graph representing the nmol of ubiquitylated CHN‐1 vs. time for CHN‐1 alone (black) or CHN‐1 + UFD‐2 (cyan). Plotted data are the mean of three technical replicates. Error bars represent the standard error of measurement (SEM); statistical significance was determined using Pearson's correlation coefficients which define the statistical relation between two continuous variables [CHN‐1 vs. time, CHN‐1 + UFD‐2 vs. time, and CHN‐1 vs. CHN‐1 + UFD‐2 with increasing time] (*P < 0.05; **P < 0.01; ***P < 0.001).BCHN‐1 auto‐Ub was performed in the presence of UFD‐2 with the increasing molar concentration as indicated. Protein samples were resolved via SDS–PAGE and immunoblotted with anti‐CHN‐1 antibodies. Right, signal quantification of the unmodified CHN‐1 (yellow), ubiquitylated fraction < 70 kDa (cyan) and > 70 kDa (magenta), plotted as a percentage of different CHN‐1 species present in the indicated condition. Plotted data are the mean from the three technical replicates. Error bars represent the SEM; statistical significance was determined using a two‐way ANOVA test (**P < 0.01).CAuto‐Ub was performed as indicated using recombinant CHN‐1 and UFD‐2^P951A^, UBE2D1 E2, Ub^WT^, Ub^NoK^, or Ub with substitutions of lysines 29, 48, and 63 to arginines (Ub^3KTR^). Protein samples were resolved via SDS–PAGE and immunoblotted with anti‐CHN‐1 antibodies.DSurface plasmon resonance (SPR) sensorgrams of the interaction between linear di‐Ub (M1‐ linear from UbiQ) and *C. elegans* UFD‐2 (magenta) or *S. cerevisiae* Ufd2p (cyan). Y‐axis: Response unit (RU) value. X‐axis: nmolar (nM) concentration of linear di‐Ub.ECHN‐1 auto‐Ub was performed as indicated in the presence of recombinant *C. elegans* UFD‐2 or *S. cerevisiae* Ufd2p and UBE2D1 E2. Protein samples were resolved via SDS–PAGE and immunoblotted with anti‐CHN‐1 antibodies. Data information: Representative immunoblots for at least three independent experiments are shown in the panels.

**Figure EV1:**
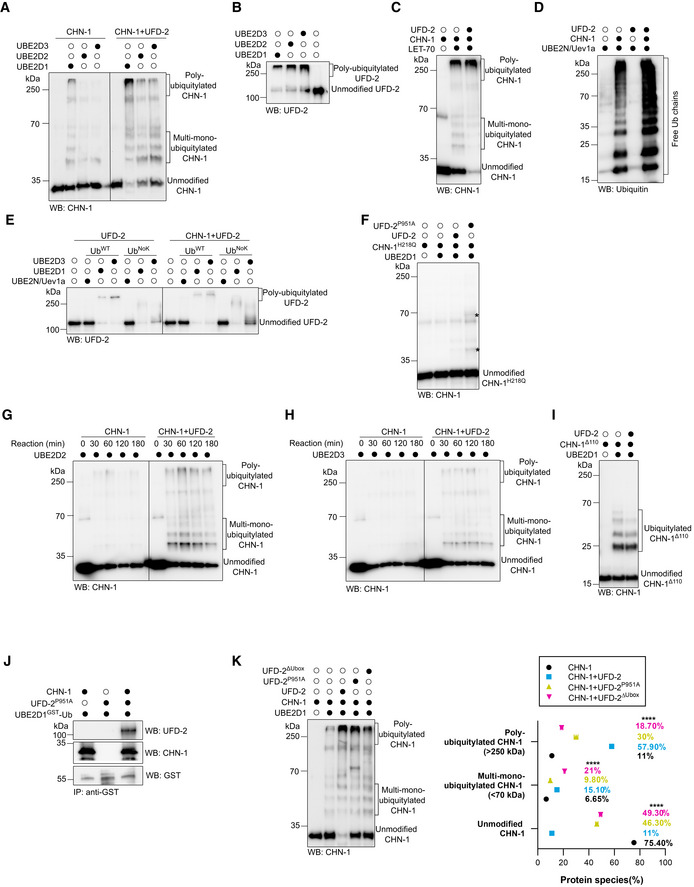
Interaction with UFD‐2 increases the ubiquitylation activity of CHN‐1 AAuto‐Ub of recombinant CHN‐1 only and in the presence of UFD‐2 was carried using UBE2D1, UBE2D2, or UBE2D3 E2. Protein samples were resolved via SDS–PAGE and immunoblotted with anti‐CHN‐1 antibodies.BAuto‐Ub of recombinant UFD‐2 was performed using UBE2D1, UBE2D2, or UBE2D3 E2. Protein samples were resolved via SDS–PAGE and immunoblotted with anti‐UFD‐2 antibodies.CAuto‐Ub of recombinant CHN‐1 only and in the presence of UFD‐2 was carried using LET‐70 E2. Protein samples were resolved via SDS–PAGE and immunoblotted with anti‐CHN‐1 antibodies.D
*In vitro* ubiquitylation assay performed in the presence of CHN‐1, UFD‐2 or both as indicated using UBE2N‐Uev1a E2. Protein samples were resolved via SDS–PAGE and immunoblotted with anti‐Ub antibodies.EAuto‐Ub of recombinant UFD‐2 only and in the presence of CHN‐1 was performed as indicated using UBE2N‐Uev1a, UBE2D1, or UBE2D3 E2, and Ub^WT^ or Ub^NoK^. Protein samples were resolved via SDS–PAGE and immunoblotted with anti‐UFD‐2 antibodies.FAuto‐Ub of recombinant CHN‐1^H218Q^ was performed in the presence of UFD‐2 or UFD‐2^P951A^ as indicated using UBE2D1 E2. Protein samples were resolved via SDS–PAGE and immunoblotted with anti‐CHN‐1 antibodies. Asterisk (*) on the blot represented the signal that appeared in the presence of UFD‐2^P951A^.GTime‐dependent (0, 30, 60, 120, and 180 min) auto‐Ub of CHN‐1 only and in the presence of UFD‐2 was performed as indicated using UBE2D2 E2. Protein samples were resolved via SDS–PAGE and immunoblotted with anti‐CHN‐1 antibodies.HTime‐dependent (0, 30, 60, 120, and 180 min) auto‐Ub of CHN‐1 only and in the presence of UFD‐2 was performed as indicated using UBE2D3 E2. Protein samples were resolved via SDS–PAGE and immunoblotted with anti‐CHN‐1 antibodies.IAuto‐Ub of recombinant CHN‐1^Δ110^ only and in the presence of UFD‐2 was performed as indicated using UBE2D1 E2. Protein samples were resolved via SDS–PAGE and immunoblotted with anti‐CHN‐1 antibodies.JCo‐immunoprecipitation of ubiquitin‐charged GST‐UBE2D1 from a mixture of ubiquitin‐charged GST‐UBE2D1 and CHN‐1, ubiquitin‐charged GST‐UBE2D1 and UFD‐2^P951A^, or the ternary mixture of ubiquitin‐charged GST‐UBE2D1, CHN‐1 and UFD‐2^P951A^ using Dynabeads conjugated with anti‐GST antibody. Protein samples were resolved via SDS–PAGE and immunoblotted with anti‐GST, anti‐UFD‐2, and anti‐CHN‐1 antibodies.KAuto‐Ub of recombinant CHN‐1 only and in the presence of UFD‐2, UFD‐2^P951A^, or UFD‐2^ΔUbox^ was performed as indicated using UBE2D1 E2. Bands were labeled as Unmodified CHN‐1, Multi‐mono‐Ubiquitylated CHN‐1, and Poly‐Ubiquitylated CHN‐1. Right, quantification of CHN‐1 modifications (Unmodified, Multi‐mono‐ubiquitylated, Poly‐ubiquitylated) plotted as percentages. Graph plotted for CHN‐1 alone (black), CHN‐1 + UFD‐2 (cyan), CHN‐1 + UFD‐2^P951A^ (yellow), or CHN‐1 + UFD‐2^ΔUbox^ (magenta). Plotted data are the mean of three technical replicates. Error bars represent the SEM; statistical significance was determined using a two‐way ANOVA test (****P < 0.0001). Data information: Representative immunoblots for at least three independent experiments are shown in the panels.

Binding between CHN‐1 and UFD‐2 was previously demonstrated via yeast two‐hybrid and *in vitro* pull‐down assays (Hoppe *et al*, 2004). Beyond the physical interaction, the molecular regulation of the potent ubiquitylation activity of the CHN‐1–UFD‐2 complex has not yet been studied in detail. A quantitative assessment of an E3 ligase activity is generally performed by examining its auto‐ubiquitylation (auto‐Ub) ability. For this purpose, an *in vitro* ubiquitylation assay with recombinant E1, E2, Ub, and E3 proteins with an ATP source can be used. The activity of the E3 enzyme can then be determined via Western blot analysis using antibodies against ubiquitin ligase or Ub itself. First, we chose E2 conjugating enzymes with which CHN‐1 and UFD‐2 are known to cooperate in the auto‐Ub reaction. Mammalian CHIP can interact with various E2s, particularly from the UbcH5/UBE2D family (UbcH5a/UBE2D1, ‐b/2, and ‐c/3) (Jiang *et al*, 2001; Soss *et al*, 2011). Similarly, CHN‐1 cooperates with UBE2D2 to mono‐ubiquitylate (mono‐Ub) *C. elegans* DAF‐2, the nematode insulin/insulin‐like growth factor 1 (IGF‐1) receptor (Tawo *et al*, 2017). To study the activity of CHN‐1 and UFD‐2, we compared their abilities to auto‐Ub in the presence of each of the UBE2D‐family proteins. We observed that CHN‐1 interacted most efficiently with UBE2D1 and least efficiently with UBE2D3, whereas UFD‐2 interacts similarly with UBE2D1‐3 (Fig EV1A and B). When we performed an auto‐Ub reaction with both E3s, we observed a significant increase in CHN‐1 poly‐ubiquitylation (poly‐Ub) activity, even when the E2 used in the reaction was UBE2D2 or UBE2D3, with which CHN‐1 alone inefficiently cooperates (Figs 1A and EV1A). The presence of UFD‐2 in the reaction also potentiated CHN‐1 auto‐Ub with LET‐70, the *C. elegans* ortholog of UBE2D proteins (Fig EV1C). Furthermore, the presence of UFD‐2 increased CHN‐1 activity with the UBE2N‐Uev1a E2 complex (Fig EV1D), which catalyzes the formation of free Ub chains that are then transferred to substrate proteins (Soss *et al*, 2011). We also concluded that the induction of E3 ligase activity is unidirectional as we did not detect any significant changes in the auto‐Ub of UFD‐2 under the same conditions (Fig EV1E). Interestingly, UFD‐2 did not interact with UBE2N‐Uev1a, indicating specificity between E2s with U‐box domain‐containing E3s (Fig EV1D and E). We also ruled out the possibility that it was UFD‐2 that modified CHN‐1 because it was unable to ubiquitylate inactive CHN‐1^H218Q^, which probably lost its affinity toward its cognate E2 (Tawo *et al*, 2017) (Fig EV1F). However, we noted that CHN‐1^H218Q^ was modified specifically in the presence of an inactive, recombinant UFD‐2 mutant with a P951A substitution (Ackermann *et al*, 2016) (bands marked with an asterisk), which might suggest recovery of CHN‐1^H218Q^ minimal activity, reflecting possible structural changes in the CHN‐1 U‐box domain during an interaction with UFD‐2.

To gain insight into CHN‐1 processivity, we performed time‐dependent auto‐Ub experiments (with reaction times of 10, 20, 40, 80, and 120 min). We observed that the presence of UFD‐2 increased both mono‐ and poly‐Ub of CHN‐1 from the 40‐min time point (Fig 1A). When we included the UBE2D2 or UBE2D3 E2s in the reaction, which CHN‐1 does not efficiently utilize (Fig EV1A), we also observed a significant increase in CHN‐1 auto‐Ub in the presence of UFD‐2, even at the earliest time point (30 min) (Fig EV1G and H). Next, we aimed to determine what molar ratio of the two E3 triggers high CHN‐1 processivity. To this end, we performed UFD‐2 titrations (0.3, 0.65, and 1.3 μM) with a fixed concentration of CHN‐1 (1.3 μM). We observed an almost twofold increase in CHN‐1 auto‐Ub in the presence of 0.65 μM UFD‐2, which roughly translates to one CHN‐1 dimer per one UFD‐2 monomer (Fig 1B). At higher UFD‐2 concentrations, we did not observe any further increase in CHN‐1 ubiquitylation; however, this effect could also be related to a Ub shortage in the reaction buffer, as UFD‐2 robustly consumes the available Ub for its auto‐Ub (Fig EV1B). Additionally, by deleting the CHN‐1 TPR domain and generating CHN‐1(Δ110aa), we confirmed the involvement of the TPR domain in UFD‐2 binding (Hoppe *et al*, 2004), as we did not observe an increase of CHN‐1(Δ110aa) activity by UFD‐2 (Fig EV1I). Therefore, we wanted to test whether UFD‐2 can regulate the processivity of CHN‐1 independent of its E3 activity. First, we found that the defect in UFD‐2^P951A^ activity is due to its inability to bind an E2 enzyme (Fig EV1J). Next, we performed a CHN‐1 ubiquitylation reaction in the presence of UFD‐2^P951A^. We detected substantial enhancement of both the mono‐ (using lysine‐less Ub (UbK0)) and the poly‐Ub activity of CHN‐1, regardless of the type of Ub chain (wild‐type Ub or variants with substitutions of lysines 29, 48, 63 to arginines (Ub3KTR) (Fig 1C). To rule out the possibility that UFD‐2^P951A^ retained residual activity, we also used a UFD‐2 variant (1–910 aa) lacking the entire U‐box domain (909–984 aa). We confirmed that this UFD‐2 deletion mutant could still stimulate CHN‐1 activity (Fig EV1K). Our results suggest that UFD‐2 binding to CHN‐1 via its TPR domain enhances the cooperation between CHN‐1 and E2s, thus resulting in more efficient auto‐Ub.

Budding yeast protein Ufd2p can operate as a Ub chain elongation factor by interacting directly with Ub through its N‐terminal region (Liu *et al*, 2017). Although higher eukaryotes have an ortholog of yeast Ufd2p, the Ub‐interacting motif has little sequence homology (Hänzelmann *et al*, 2010; Liu *et al*, 2017), suggesting that the function of UFD‐2 as an E4 is not evolutionarily conserved. To investigate whether the increased activity of the CHN‐1–UFD‐2 complex might stem from the elongation function of UFD‐2, we tested whether UFD‐2 retained its ability to interact with Ub using surface plasmon resonance (SPR) experiments. By contrast to Ufd2p, full‐length UFD‐2 did not bind linear Ub chains (Fig 1D). Unlike yeast Ufd2p, and perhaps to compensate for Ub binding loss, UFD‐2 can induce processivity of its partner CHN‐1 (Fig 1A and E). This observation suggests that UFD‐2 lost its ability to directly elongate Ub chains during evolution.

### UFD‐2 induces a structural gain of function in CHN‐1

**Figure EV2:**
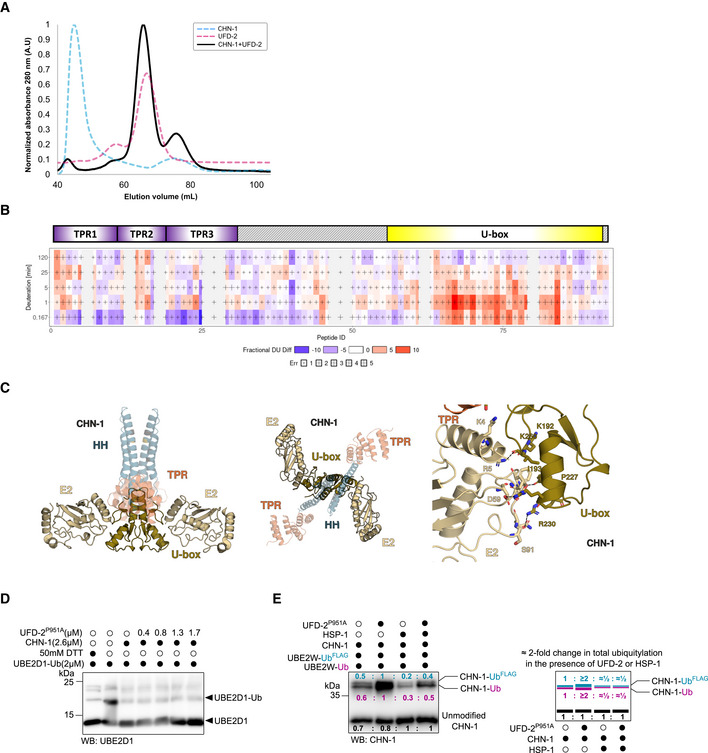
UFD‐2, unlike HSP‐1, potentiates E2 accessibility of CHN‐1 ASize‐exclusion chromatography (SEC) profiles of the recombinant proteins CHN‐1 (cyan), UFD‐2 (magenta), and CHN‐1 + UFD‐2 mixture (black) resolved in the S200 Superdex column.BChiclet plot showing the differences in deuterium uptake by CHN‐1 peptides due to the presence of UFD‐2 across the five time points. The X‐axis spans the peptide length of CHN‐1 and the time points are plotted on the Y‐axis (total of 99 peptides with 84.2% sequence coverage and 4.55 redundancy). Above the chiclet plot is the domain organization of CHN‐1, indicating TPR and U‐box domains.CModel of the CHN‐1 U‐box dimer with two E2 enzymes. UbcH5 E2 (gold) (PDB ID: 2OXQ) was aligned to the co‐crystal structure of CHIP (*Danio rerio*). The two structures aligned with low RMSD = 0.376. Marked are conserved residues that stabilize the critical interaction between the U‐box domain and E2.DDischarging assay of Ub‐charged UBE2D1 was carried with increasing molar concentrations of recombinant UFD‐2^P951A^ as indicated. The reaction was stopped after 30 min via the addition of Laemmli sample buffer. Protein samples were resolved via SDS–PAGE and immunoblotted with anti‐UBE2D1antibodies.ECHN‐1 auto‐Ub was performed as indicated in the presence of Ube2W‐Ub or Ube2W‐Ub^FLAG^ with and without a complexing equimolar concentration of recombinant CHN‐1 and UFD‐2^P951A^ and in the absence or presence of HSP‐1. Protein samples were resolved via SDS–PAGE and immunoblotted with anti‐CHN‐1 antibodies. For each sample, the quantified relative signal after probing the blot using anti‐CHN‐1 antibodies is represented as a ratio above the respective signal. Right, schematic of the CHN‐1‐Ub^FLAG^ (cyan), CHN‐1‐Ub (magenta), and unmodified CHN‐1 signal (black) presented as ratio and the signal fold change among CHN‐1, CHN‐1 + UFD‐2^P951A^, CHN‐1 + HSP‐1, and CHN‐1 + UFD‐2^P951A^ + HSP‐1. Data information: Representative immunoblots for at least three independent experiments are shown in the panels. Source data for HDX‐MS measurements are available in the Table EV1.

The different conformations afforded by dynamic and flexible motifs and oligomerization are important for the functionality of various E3 ligases (Liu & Nussinov, 2011; Kamadurai *et al*, 2013; Narayan *et al*, 2015; Koliopoulos *et al*, 2016; Faull *et al*, 2019). Therefore, we decided to analyze CHN‐1 for oligomerization and conformational flexibility after binding to UFD‐2. We observed a tendency of CHN‐1 to form oligomers, which can be seen in the size‐exclusion chromatography (SEC) as a prominent peak corresponding to its oligomeric distribution followed by a peak corresponding to the CHN‐1 dimer (Fig EV2A). When we mixed CHN‐1 and UFD‐2 in equal molar ratios and performed SEC separation, we obtained peaks corresponding to the respective proteins without the CHN‐1 oligomerization signal, suggesting a shift toward CHN‐1 dimer stabilization by UFD‐2 (Fig EV2A). Unfortunately, we did not detect a stable CHN‐1–UFD‐2 complex upon SEC separation, highlighting the dynamic and transient nature of this interaction.

**Figure 2:**
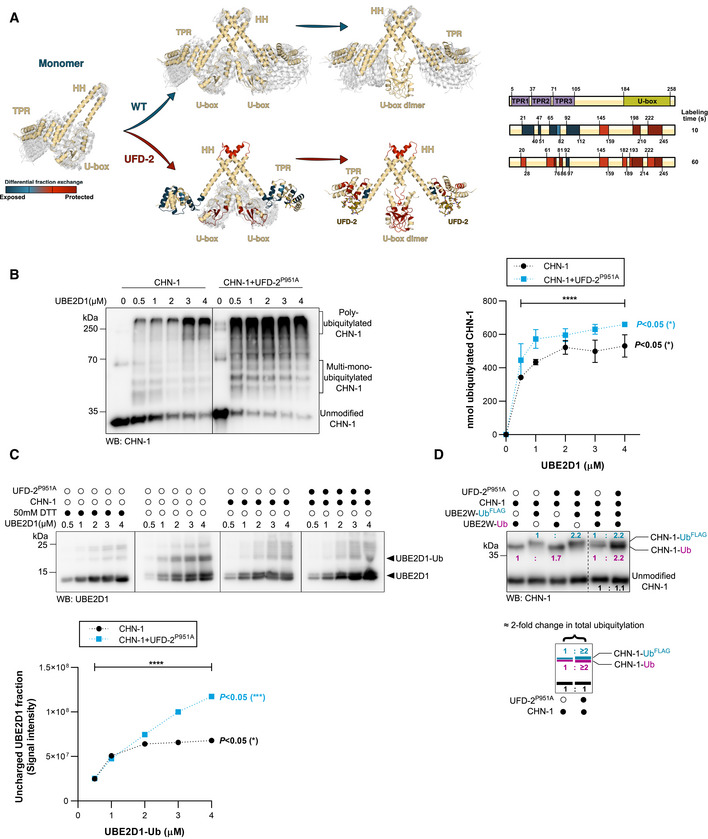
UFD‐2 stabilizes CHN‐1 U‐box dimer AHDX‐MS was used to analyze changes in the structural dynamics of residues within CHN‐1 when complexed with UFD‐2. The model diagram represents regions of retarded (red) and enhanced (blue) exchange in CHN‐1. Right, schematics showing CHN‐1 domain organization and the rate of deuterium exchange (colored box: blue, light red, medium red, and dark red) in the different domains upon interaction with UFD‐2.BCHN‐1 auto‐Ub was performed as indicated using increasing molar concentrations (0.5, 1, 2, 3, 4 μM) of UBE2D1 E2 without and with a complexing equimolar concentration (1:1) of recombinant CHN‐1 and UFD‐2^P951A^ (1.5 μM CHN‐1 with 1.5 μM of recombinant UFD‐2^P951A^). Protein samples were resolved via SDS–PAGE and immunoblotted with anti‐CHN‐1 antibodies. Right, a graph representing the nmol of ubiquitylated CHN‐1 vs. UBE2D1 (μM) for CHN‐1 alone (black) or CHN‐1 + UFD‐2^P951A^ (cyan). Plotted data are the mean from the three technical replicates. Error bars represent the SEM; statistical significance was determined using Pearson's correlation coefficients which define the statistical relation between two continuous variables [nmol of ubiquitylated CHN‐1 vs. UBE2D1(μM) in the presence of CHN‐1 or CHN‐1 + UFD‐2^P951A^ and CHN‐1 vs. CHN‐1 + UFD‐2^P951A^ with increasing UBE2D1(μM)] (*P < 0.05; ****P < 0.0001).CDischarging assay of Ub‐charged UBE2D1 was carried out in the presence of CHN‐1 or CHN‐1‐UFD‐2^P951A^. The experimental sample was run together with the control with and without a reducing agent (50 mM DTT). The reaction was stopped after 30 min via the addition of Laemmli sample buffer. Proteins were resolved via SDS–PAGE and immunoblotted with anti‐UBE2D1 antibodies. Below, quantification of uncharged UBE2D1 plotted as the uncharged UBE2D1 fraction vs. UBE2D1‐Ub (μM). Plotted data are the mean of three technical replicates. Statistical significance was determined using Pearson's correlation coefficients which define the statistical relation between two continuous variables [UBE2D1 vs. UBE2D1‐Ub(μM) in the presence of CHN‐1 or CHN‐1 + UFD‐2^P951A^ and CHN‐1 vs. CHN‐1 + UFD‐2^P951A^ with increasing UBE2D1‐Ub(μM)] (*P* < 0.05; ***P < 0.001; ****P < 0.0001).DCHN‐1 auto‐Ub was performed as indicated in the presence of Ube2W‐Ub or Ube2W‐Ub^FLAG^ without and with equimolar concentration of recombinant CHN‐1 and UFD‐2^P951A^. Protein samples were resolved via SDS–PAGE and immunoblotted with anti‐CHN‐1 antibodies. For each sample, the quantified relative signal after probing the blot using anti‐CHN‐1 antibodies is shown as a ratio above the respective signal. Below, a schematic of the CHN‐1‐Ub^FLAG^ (cyan), CHN‐1‐Ub (magenta), and unmodified CHN‐1 signal (black) presented as the ratio and the signal fold change between CHN‐1 and CHN‐1‐UFD‐2^P951A^. Data information: Representative immunoblots for at least three independent experiments are shown in the panels. Source data for HDX‐MS measurements are available in the Table EV1.

To gain mechanistic insight into the role of UFD‐2 binding to CHN‐1, we performed hydrogen‐deuterium exchange mass spectrometry (HDX‐MS) of the dimerization process of both CHN‐1 alone and CHN‐1 in the presence of UFD‐2. We detected 99 peptides with 84.2% sequence coverage of CHN‐1. Some discrepancies in the profile of detected peptides between the two conditions (CHN‐1 alone and CHN‐1 + UFD‐2) (Fig EV2B and Table EV1). In this table, peptides with missing "Frac Diff DU %" values are CHN‐1 peptides that were not detected when CHN‐1 was complexed with UFD‐2. However, most of these were redundant with other detected peptides, except for the "NNLKMT" peptide, which extends from the 52^nd^ to 57^th^ residue on CHN‐1 and is a linker between the pair of antiparallel alpha helices of the 2^nd^ TPR in CHN‐1. This may indicate that CHN‐1 interacts with UFD‐2 through this region. However, this peptide was not detected over the entire time range of the HDX‐MS experiment, and therefore, its role in the CHN‐1‐UFD‐2 complex cannot be determined. Figures 2A and EV2B depict the following model of our HDX‐MS data, which detects at least three dynamic events at 10 s and 60 s. Namely: (i) the turn in the coil‐coil motif (aa 145–159) is stabilized early on upon dimerization of the coil‐coil domains, (ii) the TPR domain is stabilized upon recognition by UFD‐2, leaving the distal helices (aa 21–40 and 92–112) exposed to the solvent. At later times, the TPR domain stabilizes against the long helix of the coil‐coil domain, (iii) the U‐box domain (aa 198–245) transitions from a weak interaction with its coil‐coil domain to a stable dimer on a longer time scale. Available crystal structures of CHIP homologs support our HDX‐MS analysis without a chaperone (Nikolay *et al*, 2004) and with HSP90 (Zhang *et al*, 2005). In the absence of a TPR‐binding chaperone, only the dimer domains are revealed by the crystal structure, with no resolution of either the turn in the coil‐coil domain or the TPR domain. Notably, the TPR domain has only been resolved by NMR, whereas it stabilizes into its crystal form in the presence of an HSP substrate (Zhang *et al*, 2005). Furthermore, structural analysis of murine CHIP shows that the bound TPR domain is further stabilized against the long helix of its coiled‐coil domain in one of its monomers (Zhang *et al*, 2005; Ye *et al*, 2017). Our computational model noted that this interaction is much weaker in CHN‐1, suggesting more structural dynamics in worms. Based on prediction with AlphaFold‐Multimer (preprint: Evans *et al*, 2022), we argue that, unlike CHIP (Zhang *et al*, 2005; Ye *et al*, 2017), CHN‐1 folds into a symmetric structure. It has been shown that HSP90 negatively regulates CHIP activity (Narayan *et al*, 2015), presumably by blocking the E2 binding site of one of the protomers (Zhang *et al*, 2005). Thus, our finding that UFD‐2 promotes CHN‐1 processivity is consistent with a symmetric structure for CHN‐1—in such a system, upon binding, UFD‐2 stabilizes the U‐box dimer with both E2 sites (Fig EV2C).

To investigate this possibility, we titrated UBE2D1 (0.5–4 μM) at a fixed concentration of CHN‐1 (1.3 μM) or CHN‐1 complexed with UFD‐2^P951A^, which cannot interact with UBE2D1, and conducted an auto‐Ub assay. We observed that at a constant Ub concentration, increasing the E2 concentration led to increased CHN‐1 activity. However, even at the highest E2 concentration (4 μM), CHN‐1 processivity did not reach the same level as in the presence of inactive UFD‐2^P951A^ and approximately eightfold lower E2 concentration (0.5 μM) (Fig 2B). Thus, the increased CHN‐1 activity of the CHN‐1–UFD‐2 complex was not due to an increased local E2 concentration but rather to the enhanced processivity of the E2 enzyme bound to the CHN‐1 U‐box domains. To verify this hypothesis, we performed an E2‐discharging assay in the presence of CHN‐1 alone or after mixing with UFD‐2^P951A^ to track the use of charged‐E2 by CHN‐1 only. We observed that in the presence of UFD‐2^P951A^, CHN‐1 could discharge at least twice as much UBE2D1‐Ub compared with CHN‐1 alone, which becomes prominent when used 2 μM concentration of UBE2D1‐Ub, which was revealed by the increasing accumulation of uncharged UBE2D1 (Figs 2C and EV2D). These results suggest that the binding of inactive UFD‐2^P951A^ can stabilize the U‐box dimer CHN‐1 and that both domains can bind and unload E2. To further verify this possibility, we performed another auto‐Ub assay with the Ube2W conjugating enzyme, which is known to maintain a strict 1:1 stoichiometry with a substrate (Christensen *et al*, 2007; Tatham *et al*, 2013; Vittal *et al*, 2013) and to catalyze mono‐Ub of CHIP (Scaglione *et al*, 2011). Considering these characteristics, we added CHN‐1 or CHN‐1/UFD‐2^P951A^ to Ub‐charged Ube2W or Ub^FLAG^‐charged Ube2W and followed the ubiquitylation profile. When both charged E2 species are included in the reaction, we should observe mono‐Ub of CHN‐1 by Ub and Ub^FLAG^ (due to the FLAG tag, Ub molecules migrate slower on SDS–PAGE, allowing differentiation of mono‐Ub from mono‐Ub^FLAG^ on a single immunoblot) and a twofold increase in the level of CHN‐1 ubiquitylation for the complex with UFD‐2^P951A^ compared with that of CHN‐1 alone. Indeed, we noted the predicted increase in CHN‐1 mono‐Ub with the two Ub variants (Fig 2D), which confirms double increase in E2 enzyme capacity of CHN‐1 in the presence of UFD‐2. It has been previously shown that HSP70 chaperone can reduce CHIP‐dependent ubiquitylation of folded substrates (Wang *et al*, 2011; Narayan *et al*, 2015; Kim *et al*, 2017). To verify whether the worm ortholog of HSP70, HSP‐1, affects the interaction of CHN‐1 with E2, we performed another auto‐Ub experiment with charged Ube2W. Indeed, the presence of HSP‐1 in the reaction reduced the Ub conjugation to CHN‐1 and altered the ratio of Ub to Ub^FLAG^ (Fig EV2E). The increase in the stoichiometric ratio of ubiquitylated CHN‐1 in the presence of UFD‐2^P951A^ suggests the existence of dimeric CHN‐1 with two available U‐box domains, while its HSP‐1‐induced decrease might suggest that HSP‐1 promotes a CHN‐1 conformation that limits E2 access to the U‐box domains.

### HSP‐1 and UFD‐2 modulate CHN‐1 processivity by stabilizing its inactive and active conformation, respectively

**Figure 3:**
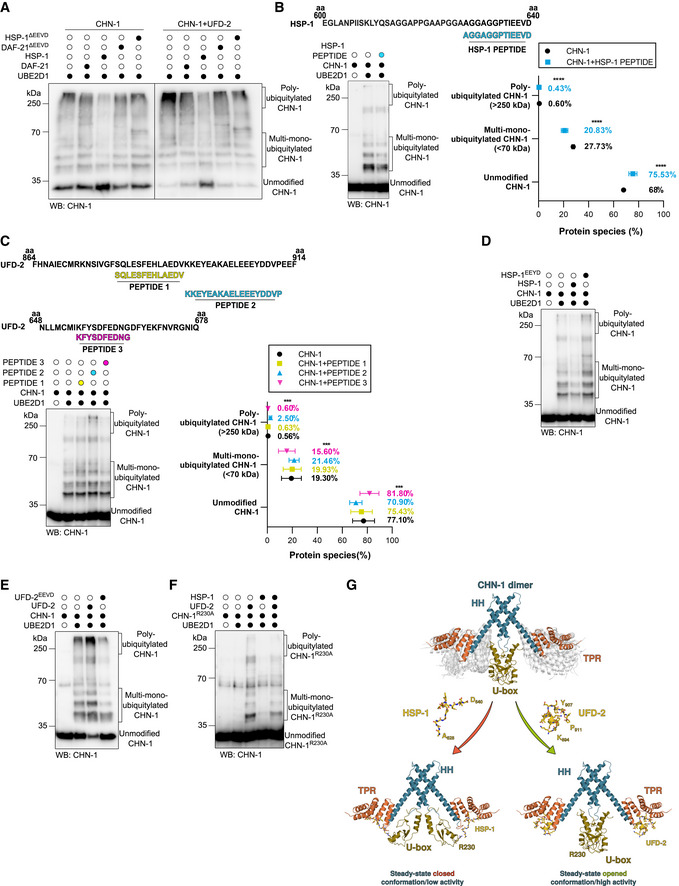
UFD‐2 stabilizes an open/active CHN‐1 conformation and HSP‐1 stabilizes a closed/non‐active CHN‐1 conformation AAuto‐Ub of recombinant CHN‐1 using UBE2D1 E2 was performed as indicated, alone or when complexed with UFD‐2, and in the presence of recombinant DAF‐21, DAF‐21^ΔEEVD^, HSP‐1, or HSP‐1^ΔEEVD^. Protein samples were resolved via SDS–PAGE and immunoblotted with anti‐CHN‐1 antibodies.BTop, schematics of the HSP‐1 peptide sequence (cyan) aligned with the C‐terminal sequence of full‐length HSP‐1 (amino acids 600–640) used in the ubiquitylation reaction. Auto‐Ub of recombinant CHN‐1 was performed as indicated in the presence of HSP‐1‐derived peptide and UBE2D1 E2. Protein samples were resolved via SDS–PAGE and immunoblotted with anti‐CHN‐1 antibodies. Right, quantification of the CHN‐1 modifications (Unmodified, Multi‐mono‐ubiquitylated, Poly‐ubiquitylated) when CHN‐1 alone (black) or CHN‐1 + HSP‐1 peptide (cyan). Plotted data are the mean of three technical replicates. Error bars represent the SEM; statistical significance was determined using a two‐way ANOVA test (****P < 0.0001).CTop, schematics of the UFD‐2 peptide sequences aligned with the C‐terminal sequence of full‐length UFD‐2 (amino acids 864–914) for PEPTIDE 1 (yellow) and PEPTIDE 2 (cyan), and (UFD‐2 amino acids 648–678) for PEPTIDE 3 (magenta) used in the ubiquitylation reaction. Below, auto‐Ub of recombinant CHN‐1 was performed as indicated in the presence of UFD‐2‐derived peptides using UBE2D1 E2. Protein samples were resolved via SDS–PAGE and immunoblotted with anti‐CHN‐1 antibodies. Right, quantification of the CHN‐1 modifications (Unmodified, Multi‐mono‐ubiquitylated, Poly‐ubiquitylated) when CHN‐1 alone (black), CHN‐1 + PEPTIDE 1 (yellow), CHN‐1 + PEPTIDE 2 (cyan), or CHN‐1 + PEPTIDE 3 (magenta). Plotted data are the mean of three technical replicates. Error bars represent the SEM; statistical significance was determined using a two‐way ANOVA test (***P < 0.001).DAuto‐Ub of recombinant CHN‐1 was performed as indicated using UBE2D1 E2 in the presence of recombinant HSP‐1 or HSP‐1^EEYD^. Protein samples were resolved via SDS–PAGE and immunoblotted with anti‐CHN‐1 antibodies.EAuto‐Ub of recombinant CHN‐1 was performed as indicated using UBE2D1 E2 in the presence of recombinant UFD‐2 or UFD‐2^EEVD^. Protein samples were resolved via SDS–PAGE and immunoblotted with anti‐CHN‐1 antibodies.FCHN‐1^R230A^ auto‐Ub was performed as indicated using UBE2D1 E2 in the presence of recombinant UFD‐2 and HSP‐1. Protein samples were resolved via SDS–PAGE and immunoblotted with anti‐CHN‐1 antibodies.GModel of UFD‐2 activation and HSP‐1 inhibition of CHN‐1. Dimeric CHN‐1 with the TPR domain, U‐box, and helix‐turn‐helix (HH) is indicated by magenta, gold, and cyan, respectively. UFD‐2 and HSP‐1 peptides are shown in yellow with the indicated amino acid positions in the full‐length proteins. Data information: Representative immunoblots for at least three independent experiments are shown in the panels.

**Figure EV3:**
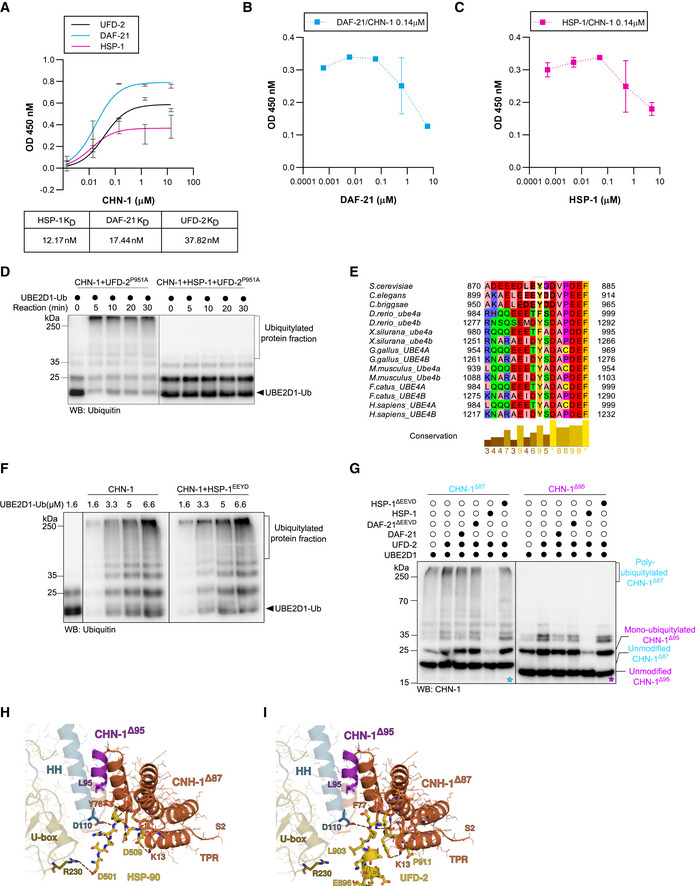
CHN‐1 activity switch is induced by the interaction of its TPR domain with the HSP‐1 EEVD or UFD‐2 EEYD motif AELISA‐based titration assay to determine the dissociation constants (K_D_) between DAF‐21, HSP‐1, UFD‐2, and CHN‐1. Y‐axis: CHN‐1 concentration (μM). X‐axis: absorbance (OD) value at 450 nm as a function of the converted substrate (Alkaline Phosphatase Yellow). Below, a table showing the K_D_ value (nM) of the corresponding protein with recombinant CHN‐1. Plotted data are the mean of three technical replicates. Error bars represent the SEM.BELISA‐based titration assay performed using recombinant CHN‐1, UFD‐2, and DAF‐21 with the results plotted as the DAF‐21 concentration (μM) vs. absorbance (OD) value at 450 nm as a function of the converted substrate (Alkaline Phosphatase Yellow). Plotted data are the mean of three technical replicates. Error bars represent the SEM.CELISA‐based titration assay performed using recombinant CHN‐1, UFD‐2, and HSP‐1 with the results plotted as the HSP‐1 concentration (μM) vs. absorbance (OD) value at 450 nm as a function of the converted substrate (Alkaline Phosphatase Yellow). Plotted data are the mean of three technical replicates. Error bars represent the SEM.D
*In vitro* ubiquitylation assay was performed as indicated using Ub‐charged UBE2D1 in the presence of CHN‐1, UFD‐2^P951A^ or ternary mixture of recombinant CHN‐1, UFD‐2^P951A^ and HSP‐1. The reaction was stopped after the indicated time via the addition of Laemmli sample buffer. Protein samples were resolved via SDS–PAGE and immunoblotted with anti‐Ubiquitin antibodies.EMultiple sequence alignment (MSA) of UFD‐2 from different species. Orthologous sequences from selected species were obtained from the eggNOG5 database (from Orthologous Group ID ENOG5038DSP) (Huerta‐Cepas *et al*, 2019) and aligned using the T‐Coffee web server with default parameters (Notredame *et al*, 2000; Di Tommaso *et al*, 2011). Vertebrates possess two UFD‐2 orthologs, which have been independently annotated. The MSA was visualized in the Jalview Desktop software (Waterhouse *et al*, 2009) with residues colored according to their physicochemical properties; conserved tyrosine (Y) residues and the EEYD motif in *C. elegans* are highlighted in white frames.F
*In vitro* ubiquitylation assay was performed as indicated using an increasing concentration of Ub‐charged UBE2D1 (1.6, 3.3, 5, 6.6 μM) in the presence of CHN‐1 or CHN‐1 and HSP‐1^EEYD^. The reaction was stopped after 30 min via the addition of Laemmli sample buffer. Protein samples were resolved via SDS–PAGE and immunoblotted with anti‐Ubiquitin antibodies.GAuto‐Ub of recombinant CHN‐1^Δ87^ (cyan) or CHN‐1^Δ95^ (magenta) truncation mutants as indicated using UBE2D1 E2 in the presence of UFD‐2, DAF‐21, DAF‐21^ΔEEVD^, HSP‐1 or HSP‐1^ΔEEVD^. Samples were analyzed via SDS–PAGE and immunoblotted with anti‐CHN‐1 antibodies. Cyan asterisk (*) on the blot represented the auto‐ub of CHN‐1^Δ87^ and magenta asterisk (*) on the blot represented the auto‐ub of CHN‐1^Δ95^.HModel of the CHN‐1 TPR domain docked with the UFD‐2 EEYD peptide. Residues 1–86 are colored in orange and residues 87–95 of CHN‐1, which sequester the EEYD motif away from the CHN‐1 R230 residue, are colored in magenta.IA co‐crystal structure of the murine CHIP TPR domain interacting with the HSP90 EEVD peptide (PDB ID 2C2L) reveals that CHIP R273 (conserved in CHN‐1 as R230) is sufficiently close in proximity to interact with HSP90 D501. Data information: Representative immunoblots for at least three independent experiments are shown in the panels.

The three TPR domains in CHIP act as a binding platform for C‐terminal peptides of the HSP70 and HSP90 chaperones, which contain a conserved EEVD motif (Zhang *et al*, 2005, 2015; Paul & Ghosh, 2014). As CHN‐1 also binds UFD‐2 via the TPR domain, we investigated whether HSP‐1 or DAF‐21 (the nematode HSP90 ortholog) could interfere with the interaction between CHN‐1 and UFD‐2. We first examined protein‐protein interactions between CHN‐1 and UFD‐2, HSP‐1, or DAF‐21 using enzyme‐linked immunosorbent assays (ELISAs). CHN‐1 showed a higher affinity for HSP‐1 and DAF‐21 compared with UFD‐2 (Fig EV3A). Next, we tested whether the chaperones could compete with UFD‐2 for CHN‐1 binding. We performed an ELISA‐based titration assay to determine the dissociation of CHN‐1 from immobilized UFD‐2 induced by the presence of HSP‐1 or DAF‐21. As the concentration of chaperones increased, the CHN‐1 signal decreased (increased dissociation from the complex with UFD‐2), indicating that chaperones compete with UFD‐2 for the CHN‐1 (Fig EV3B and C). To verify the influence of HSP‐1 and DAF‐21 on the activity of the CHN‐1‐UFD‐2 pair, we performed auto‐Ub reactions in the presence of the chaperones. HSP‐1 significantly reduced the auto‐Ub activity of CHN‐1 and blocked the stimulatory capacity of UFD‐2 in this process (Figs 3A and EV3D). Removal of the C‐terminal EEVD motif mitigated the inhibitory effect of HSP‐1. By contrast, DAF‐21 did not affect the UFD‐2‐dependent enhancement of CHN‐1 activity (Fig 3A).

Next, we performed peptide mapping on peptide microarrays to pinpoint the interaction interface between the two ligases. For this, we used purified CHN‐1 tagged with His::SUMO and His::SUMO alone (as a control). These proteins were incubated on two UFD‐2 peptide microarrays consisting of 7‐ and 13‐aa peptides. Signal enrichment analysis suggested that the two UFD‐2 consensus sequences, EAKAELEEE and EEYDDVPE, were the predominant interactor motifs. HSP70/90 uses a similar acidic C‐terminal peptide with the EEVD sequence to bind to the TPR domain of target proteins (Scheufler *et al*, 2000; Gazda *et al*, 2013), and the HSP‐1 C‐terminal EEVD peptide affected CHN‐1 activity (Fig 3B). Therefore, we examined whether the identified UFD‐2 peptides could also regulate CHN‐1. To this end, we performed CHN‐1 auto‐Ub reactions in the presence of the UFD‐2‐derived peptides identified in the peptide microarray data. We found that only the KKEYEAKAELEEEYDDVP peptide from UFD‐2 significantly stimulated CHN‐1 auto‐Ub (Fig 3C). An EEYD sequence is present in this peptide, suggesting that UFD‐2 can utilize an EEVD‐like motif for CHN‐1 binding. Furthermore, multiple sequence alignment revealed that in the EEYD motif of UFD‐2, the tyrosine (Y) is evolutionarily conserved among higher eukaryotes (Fig EV3E). To define the functional role of the UFD‐2 EEYD motif, we generated a chimeric recombinant HSP‐1 protein carrying an N‐terminal EEYD instead of EEVD and UFD‐2 with the opposite change (EEYD to EEVD). Notably, we observed stimulation of CHN‐1 auto‐Ub by HSP‐1^EEYD^, opposite to the inhibitory effect of wild‐type HSP‐1 (Figs 3D and EV3F). By contrast, UFD‐2^EEVD^ no longer stimulated CHN‐1 activity and even mildly reduced it (Fig 3E). This observation suggests a key role for the conserved tyrosine residue at the C‐terminus of UFD‐2 in the binding and regulating of the CHN‐1 activity switch.

Next, we assessed the contribution of specific regions of the CHN‐1 TPR domain in chaperone and UFD‐2 binding by generating truncation variants. We showed that the first 87 amino acids (Δ87 variant) are not responsible for the interaction with UFD‐2 and HSP‐1 and, therefore, are not involved in the modulation of CHN‐1 processivity. By contrast, removing the subsequent eight residues (Δ95 variant) abrogated the CHN‐1 poly‐auto‐Ub activity. Interestingly, the stimulating effect of UFD‐2 was still observed, as indicated by an increase in mono‐Ub CHN‐1^Δ95^ (Fig EV3G). CHN‐1^Δ95^ has residues that might be involved in an interaction with UFD‐2, including D110 and subsequent coils and helices; thus, the CHN‐1^Δ110^ mutant (lacking D110) does not show any gain of activity in the presence of UFD‐2 (Fig EV1I). It is known that a position homologous to D110 in mouse CHIP (D135) is involved in HSP90 binding (Fig EV3H), suggesting that this residue is also important for the interaction with UFD‐2 (Fig EV3I).

To understand why HSP‐1 and UFD‐2 peptides exhibit distinct effects on CHN‐1 activity, we looked closely at the mechanism by which increased HSP90 or HSP70 concentrations reduce CHIP activity (Narayan *et al*, 2015). HSP90 stabilizes an auto‐inhibited monomer in murine CHIP (Zhang *et al*, 2005). This state involves a salt bridge between HSP90 D501 and CHIP R273, latching the U‐box and TPR domains (Fig EV3H). This observation suggests that chaperone binding can directly restrain the U‐box from participating in Ub processivity. To show that a similar mechanism is at play in inhibiting ubiquitylation by HSP‐1, we mutated R230 (homologous position to R273 in CHIP) to alanine to weaken the CHN‐1 U‐box interaction with the HSP‐1 peptide, thus abrogating its inhibitory effect. Indeed, we observed reduced inhibition of the CHN‐1^R230A^–UFD‐2 complex by HSP‐1 (Fig 3F). This finding is consistent with the model that HSP‐1 stabilizes the autoinhibited state of CHN‐1 by interacting with the TPR and U‐box domains, thereby affecting its interaction with E2 enzymes (Fig 3G). On the other hand, UFD‐2 can avoid interacting with R230 by, for example, forming a helix that cannot extend toward the U‐box, thus inducing uncorrelated mobility of the TPR domains with respect to the U‐box domains that promotes a steady‐state open conformation of CHN‐1 (Fig 3G), which explains maintaining its boosting effect on CHN‐1^R230A^.

### UFD‐2 promotes CHN‐1 turnover independent of E3 activity

**Figure 4:**
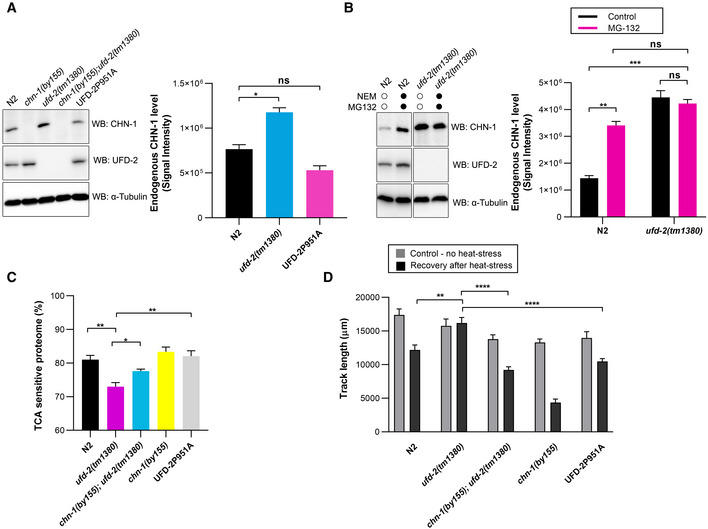
UFD‐2 regulates CHN‐1 turnover ATo detect CHN‐1 and UFD‐2, indicated lysates of young adult worms were subjected to immunoblotting with anti‐CHN‐1 and anti‐UFD‐2 antibodies. Tubulin served as a loading control. Right, quantification of the CHN‐1 signals normalized to tubulin levels and plotted as N2 (wild‐type; black), *ufd‐2(tm1380)* (cyan), and UFD‐2P951A (magenta). Plotted data are the mean of three biological replicates. Error bars represent the SEM; statistical significance was determined using an unpaired *t*‐test (*P < 0.05).BCHN‐1 protein levels were determined in the indicated lysates of young adult worms treated with a proteasome inhibitor (MG‐132, 10 μM) and DUB inhibitor (NEM, 100 mM). Tubulin served as a loading control. Right, quantification of the CHN‐1 signals normalized to tubulin levels plotted as control (black), or MG‐132 treated (magenta) in N2 (wild‐type) and *ufd‐2(tm1380)*. Plotted data are the mean of three biological replicates. Error bars represent the SEM; statistical significance was determined using an unpaired *t*‐test (**P < 0.01; ***P < 0.001).CGraph showing the comparison of protein solubility in the presence of 10% trichloroacetic acid (TCA) presented as TCA sensitive proteome in percentage (%) in N2 (wild‐type; black), *ufd‐2(tm1380)* (magenta), *chn‐1(by155); ufd‐2(tm1380)* (cyan), *chn‐1(by155)* (yellow) and UFD‐2P951A (gray) worms. Y‐axis shows the percent of the entire protein sample sensitive to TCA treatment. Plotted data are the mean of three biological replicates. Error bars represent SEM; statistical significance was determined using a one‐way ANOVA test (*P < 0.05; **P < 0.01).DMeasurement of the mobility of the indicated young adult worms exposed to 33°C heat stress (2 h). Graph plotted as control (gray), or recovery after heat stress (black) for N2 (wild‐type), *ufd‐2(tm1380)*, *chn‐1(by155); ufd‐2(tm1380)*, *chn‐1(by155)* and UFD‐2P951A worms. Plotted data are the mean of three biological replicates. Error bars represent SEM; statistical significance was determined using a one‐way ANOVA test (**P < 0.01; ****P < 0.0001).

Our *in vitro* studies indicate that interaction with UFD‐2 enhances CHN‐1 auto‐Ub; therefore, *in vivo* interaction with UFD‐2 could promote CHN‐1 turnover. To verify this hypothesis, we performed a Western blot analysis of CHN‐1 and UFD‐2 protein levels in young adult worms. As expected, CHN‐1 abundance was significantly increased in *ufd‐2(tm1380)* null allele worms. Next, we used CRISPR/Cas9 editing to generate animals expressing the catalytically inactive UFD‐2P951A. Consistent with our prediction, in worms expressing UFD‐2P951A, the CHN‐1 protein level was significantly lower than in *ufd‐2(tm1380)* animals and comparable to wild‐type worms (Fig 4A). Furthermore, proteasome inhibition by MG132 stabilized CHN‐1 in wild‐type worms to levels similar to those in *ufd‐2(tm1380)* worms (Fig 4B). Finally, in *ufd‐2(tm1380)* animals, MG132 treatment did not increase the CHN‐1 level further (Fig 4B). These data indicate that independent of ubiquitylation activity, UFD‐2 is involved in CHN‐1 turnover, presumably by promoting CHN‐1 auto‐Ub, which might lead to its proteasomal degradation. This outcome might have negative consequences for proteostasis, for example, during proteotoxic stress, as it would decrease the level of CHN‐1, which is an important quality control E3 ligase. Therefore, we hypothesized that *ufd‐2* deletion might enhance the proteostasis capacity of the organism by reducing CHN‐1 turnover. To test this possibility, we examined the worm proteome that was sensitive to trichloroacetic acid (TCA) precipitation, an established method to assess the fraction of unfolded and aggregation‐prone proteins (Cortese *et al*, 2005; Rajalingam *et al*, 2009; Depuydt *et al*, 2016). Indeed, we observed an increased fraction of stable proteins (insensitive to TCA) in the proteome of *ufd‐2(tm1380)* worms compared with wild‐type, and this effect was suppressed by *chn‐1* deletion (*chn‐1(by155); ufd‐2(tm1380))*. In contrast to the *ufd‐2(tm1380)* worms, animals expressing the UFD‐2P951A displayed similar levels of unstable proteins compared with wild‐type worms (Fig 4C). To further explore the functional importance of CHN‐1 and UFD‐2 cooperation for organismal proteostasis, we measured the motility recovery rate of synchronized worms after heat stress. The results showed improved recovery in *ufd‐2(tm1380)* nematodes compared with controls. As expected, this effect was suppressed in the *chn‐1(by155); ufd‐2(tm1380*) double mutant, while worms expressing UFD‐2P951A showed similar recovery to that of control animals (Fig 4D). In summary, our results suggest that in an E3‐independent manner, stimulation of CHN‐1 processivity by UFD‐2 can simultaneously potentiate CHN‐1 auto‐Ub. One possible consequence of this interaction could be a limiting effect on the proteostasis network induced by CHN‐1 turnover.

### The CHN‐1/UFD‐2 pair regulates phosphatidylcholine synthesis via AHCY‐1

**Figure 5:**
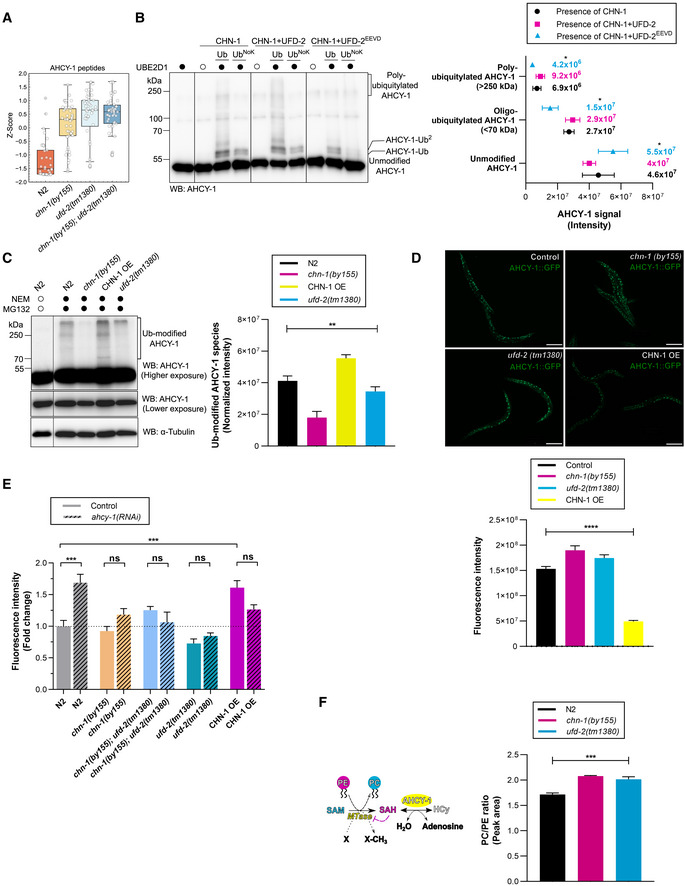
The CHN‐1/UFD‐2 pair regulates lipid metabolism via AHCY‐1 ABoxplot analysis showing the Z‐score of normalized intensities of the 50 LC‐MS/MS‐identified peptides from ACHY‐1 detected in N2 (wild‐type), *chn‐1(by155)*, *ufd‐2(tm1380)*, and *chn‐1(by155); ufd‐2(tm1380)* mutant worms. The central band of each box is the median value, and the box defines the 25^th^ (lower) and 75^th^ (higher) quantile. The whiskers represent the minimum and maximum values in the data, excluding outliers. A data point is considered an outlier if the distance to the median is greater than 1.5 * inter quantile range distance to the median.BUbiquitylation of recombinant AHCY‐1 was performed as indicated. Protein samples were resolved via SDS–PAGE and immunoblotted with anti‐AHCY‐1 antibodies. Right, quantification of the AHCY‐1 modifications (unmodified, oligo‐monoubiquitylated, poly‐ubiquitylated) when CHN‐1 alone (black), CHN‐1–UFD‐2 (magenta), or CHN‐1–UFD‐2^EEVD^ (cyan). Plotted data are the mean of three technical replicates. Error bars represent SEM; statistical significance was determined using a two‐way ANOVA test (*P < 0.05).CProtein level of endogenous AHCY‐1 in N2 (wild‐type), *chn‐1(by155)*, CHN‐1 OE, and *ufd‐2(tm1380)* young adult worms treated with the proteasome inhibitor (MG‐132, 10 μM) and DUB inhibitor (NEM, 100 mM). Protein samples were resolved via SDS–PAGE and immunoblotted with anti‐AHCY‐1 antibodies. Tubulin served as a loading control. Right, quantification of the modified AHCY‐1 signals plotted as Ub‐modified AHCY‐1 species normalized to unmodified endogenous AHCY‐1 signal and plotted for N2 (wild‐type; black), *chn‐1(by155)* (magenta), CHN‐1 OE (yellow), and *ufd‐2(tm1380)* (cyan). Plotted data are the mean of three biological replicates. Error bars represent SEM; statistical significance was determined using an unpaired *t‐*test (**P < 0.01).DRepresentative images of GFP::AHCY‐1 fluorescence in *chn‐1(by155)*, *ufd‐2(tm1380)*, and CHN‐1 OE background. Scale bar = 200 μm. Below, quantification of the AHCY‐1 GFP signal plotted as fluorescence intensity for GFP::AHCY‐1 expressing worms (control; black), *chn‐1(by155)* (magenta), *ufd‐2(tm1380)* (cyan) or CHN‐1 OE (yellow). Plotted data are the mean of three biological replicates. Error bars represent SEM; statistical significance was determined using a one‐way ANOVA test (****P < 0.0001).ETotal lipid content in N2 (wild‐type), *chn‐1(by155)*, *ufd‐2(tm1380)*, *chn‐1(by155)*, *ufd‐2(tm1380)*, and CHN‐1 OE young adult worms grown on control (plain) and *ahcy‐1(RNAi)* (lined) feeding plates. Higher fluorescence intensity indicates increased lipid levels. Plotted data are the mean of three biological replicates. Error bars indicate SEM; statistical significance was determined using a one‐way ANOVA test (***P < 0.001).FSchematic diagram representing the core function of AHCY‐1. AHCY‐1 catalyzes the reversible hydrolysis of SAH (*S*‐adenosylhomocysteine) to HCy (homocysteine). SAH accumulation inhibits PC (phosphatidylcholine) synthesis from PE (phosphatidylethanolamine). Right, ratio of phosphatidylcholine (PC) to phosphatidylethanolamine (PE) in N2 (wild‐type; black), *chn‐1(by155)* (magenta), and *ufd‐2(tm1380)* (cyan) young adult worms. Plotted data are the mean of three biological replicates. Error bars indicate SEM; statistical significance was determined using a one‐way ANOVA test (***P < 0.001).

**Figure EV4:**
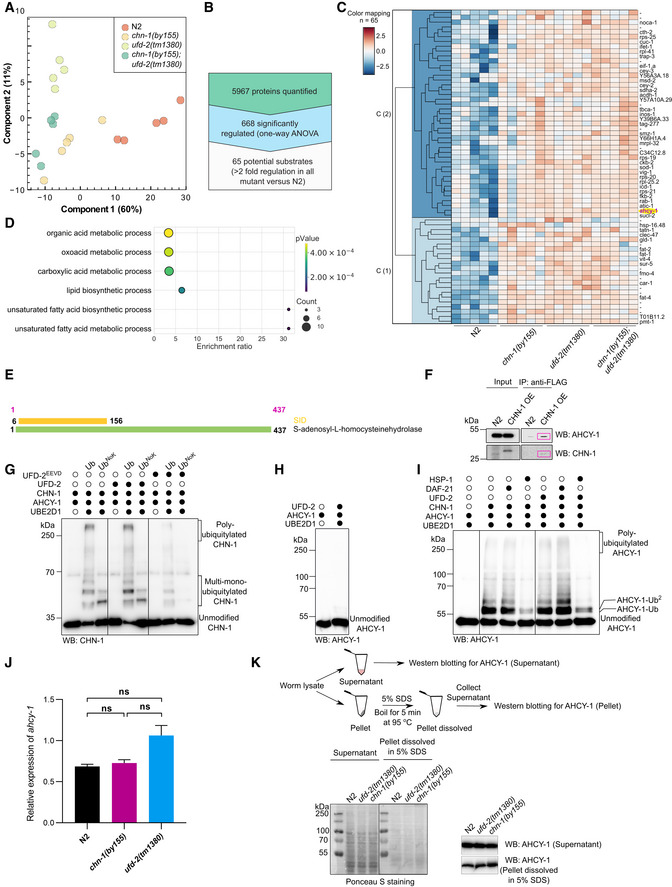
Poly‐ubiquitylation of AHCY‐1 is mediated by the CHN‐1‐UFD‐2 complex APCA showing the first and second principal components of the significantly altered proteins (ANOVA FDR < 0.05) performed in the Perseus software (Tyanova *et al*, 2016). The percentage of explained variance is represented on the axis labels.BSchematic representation of the number of identified proteins in a single‐shot analysis of LC‐MS/MS gradients in five biological replicates that led to the identification of proteins with significant abundance changes in *chn‐1(by155)*, *ufd‐2(tm1380)*, and *chn‐1(by155); ufd‐2(tm1380)* worms (twofold enrichment in all mutants versus wild‐type N2 animals).CHierarchical clustering of the Z‐scores of proteins whose levels increased in *chn‐1(by155)*, *ufd‐2(tm1380)*, and *chn‐1(by155); ufd‐2(tm1380)* mutant worms (twofold enrichment in all mutants versus wild‐type N2 animals from the LC‐MS/MS experiment).DGene ontology biological process terms found to be associated with *C. elegans* genes upregulated (minimum twofold enrichment versus N2 (control), with FDR < 0.05 for ANOVA or pairwise *t*‐test) in all mutants; all proteins detected in the LC‐MS/MS analysis comprised a reference set. Overrepresentation analysis was performed using the WebGestalt web server with default parameters (Liao *et al*, 2019). FDR was controlled to 0.25 using the Benjamini‐Hochberg method for multiple testing.EYeast two‐hybrid prey fragment analysis. Schematic representations of the AHCY‐1 fragments interacting with CHN‐1 identified in the yeast two‐hybrid screen (Hybrigenics). The coding sequence for CHN‐1 was used as bait to screen a random‐primed *C. elegans* mixed‐stage cDNA library. The selected interaction domain (SID) is shown in yellow.FCo‐immunoprecipitation of AHCY‐1 from young adult worms expressing CHN‐1::FLAG using beads conjugated with anti‐FLAG antibody. Protein samples were resolved via SDS–PAGE and immunoblotted with anti‐AHCY‐1 and anti‐FLAG antibodies (the red boxes mark the protein band).GCHN‐1 auto‐Ub was performed as indicated. Protein samples were resolved via SDS–PAGE and immunoblotted with anti‐CHN‐1 antibodies.HUbiquitylation of recombinant AHCY‐1 was performed as indicated using recombinant UFD‐2 and UBE2D1 E2. Protein samples were resolved via SDS–PAGE and immunoblotted with anti‐AHCY‐1 antibodies.IUbiquitylation of recombinant AHCY‐1 was performed as indicated using recombinant CHN‐1, UFD‐2, DAF‐21, or HSP‐1 in the presence of UBE2D1 E2. Protein samples were resolved via SDS–PAGE and immunoblotted with anti‐AHCY‐1 antibodies.JQuantitative PCR analyses of *ahcy‐1* transcript levels in young adult N2 (wild‐type; black), *chn‐1(by155)* (magenta) and *ufd‐2(tm1380)* (cyan) worms. Plotted data are the mean of three biological replicates. Error bars represent SEM; statistical significance was determined using a one‐way ANOVA test.KProtein level of endogenous AHCY‐1 in N2 (wild‐type), *chn‐1(by155)*, and *ufd‐2(tm1380)* young adult worms. After centrifugation, the supernatant obtained from the worm lysate and the resulting pellet were dissolved in 5% SDS and boiled for 5 min. Protein samples were resolved via SDS–PAGE and immunoblotted with anti‐AHCY‐1 antibodies. Data information: Representative immunoblots for at least three independent experiments are shown in the panels.

Based on our results, we hypothesized that CHN‐1, when functioning unaided, would exhibit low poly‐Ub activity, mainly catalyzing mono‐Ub of its substrates. Indeed, earlier, we showed that CHN‐1 alone mono‐Ub the DAF‐2 insulin receptor in worms (Tawo *et al*, 2017). We further assumed that interaction with UFD‐2 would stimulate the poly‐Ub activity of CHN‐1, consequently leading to efficient degradation of its specific substrates. Thus, to delineate the role of CHN‐1 and UFD‐2 *in vivo*, we decided to identify such substrates. We searched for proteins whose levels increased after *chn‐1* deletion (substrate ubiquitylation by CHN‐1 would be affected directly) or *ufd‐2* (CHN‐1 would not be stimulated to efficiently poly‐Ub its substrates). To unbiasedly define the consequences of *chn‐1* and *ufd‐2* deletion on the *C. elegans* proteome and to detect proteins that accumulate in the deletion mutants, we performed label‐free mass spectrometry (LC‐MS/MS)‐based proteomics experiment. We analyzed *chn‐1(by155)*, *ufd‐2(tm1380)*, and *chn‐1(by155); ufd‐2(tm1380)* double‐mutant worms via single‐shot LC‐MS/MS gradients with five biological replicates. To obtain a view on the global structure of the data, we performed dimensional reduction using principal component analysis (PCA). We observed that the proteomes of the *chn‐1(by155)*, *ufd‐2(tm1380)*, and *chn‐1(by155); ufd‐2(tm1380)* mutants clustered closer together with respect to the proteomes from wild‐type animals (N2 strain) (Fig EV4A). We hypothesized that potential substrates should accumulate in all mutants; therefore, we filtered the set of significantly altered proteins requiring a two‐fold enrichment in all mutants versus the N2 control strain. We obtained 65 potential substrate candidates, which we visualized via hierarchical clustering (Fig EV4B and C). These potential substrates were enriched in metabolic processes, including lipid biosynthesis, as shown in Gene Ontology over‐representation analysis (Fig EV4D). Among them, we identified the AHCY‐1 enzyme (Figs 5A and EV4C). AHCY‐1 catalyzes the reversible hydrolysis of *S*‐adenosylhomocysteine (SAH) to homocysteine and adenosine (Palmer & Abeles, 1976, 1979). Despite the fundamental role of AHCY‐1 in metabolism, its regulatory mechanisms are still enigmatic. In a yeast two‐hybrid screen using a *C. elegans* cDNA library, we identified AHCY‐1 as the prominent interactor of CHN‐1 (Fig EV4E). We confirmed the interaction between the two proteins in worms via co‐immunoprecipitation (Fig EV4F). Next, we tested whether AHCY‐1 is a CHN‐1 substrate by performing *in vitro* ubiquitylation assays. We confirmed that recombinant AHCY‐1 is a specific substrate of CHN‐1 that UFD‐2 does not ubiquitylate (Fig EV4G and H). Furthermore, in the presence of UFD‐2, CHN‐1 poly‐Ub AHCY‐1 more effectively, and the level of this modification was reduced by UFD‐2^EEVD^ or HSP‐1 (Figs 5B, EV4G and I). The cooperation between CHN‐1 and UFD‐2 is also consistent with the detection of a similar increase in the AHCY‐1 level in *chn‐1(by155)*, *ufd‐2(tm1380)*, and double‐mutant worms in our proteomic analysis (Fig 5A). To further validate this observation, we monitored the endogenous AHCY‐1 level via Western blotting of total lysates of wild‐type worms, *chn‐1(by155)*, and *ufd‐2(tm1380)* mutant worms, as well as worms overexpressing *chn‐1* (CHN‐1 OE), treated with the proteasome (MG132) and deubiquitinating enzyme (DUB) (*N*‐methylmaleimide, NEM) inhibitors. We did not observe any significant changes in the AHCY‐1 level, which, according to our other observations, is a stable and abundant protein in *C. elegans*. However, immunoblotting with anti‐AHCY‐1 antibodies detected higher molecular weight smeared bands when *chn‐1* was overexpressed, likely corresponding to poly‐Ub AHCY‐1 species (Fig 5C). Furthermore, these bands were reduced in *chn‐1(by155)* and *ufd‐2(tm1380)* mutant worms compared with the ACHY‐1 status in wild‐type animals (Fig 5C). As we did not observe a change in the stability of unmodified AHCY‐1 in worm lysates, which could be related to the tendency of AHCY‐1 to precipitate during sample preparation (Fig EV4K), we generated a CRISPR/Cas9 knock‐in GFP‐based worm line to label AHCY‐1 to track its localization and abundance without compromising the integrity of the worms while maintaining its natural expression level. The GFP tag did not affect AHCY‐1 functionality, as its knockout is lethal to worms (WormBase and our observations). Next, we crossed *chn‐1(by155)*, *ufd‐2(tm1380)*, and CHN‐1 OE worms with animals expressing GFP::AHCY‐1. Microscopic analysis of GFP::AHCY‐1 fluorescence levels revealed a significant increase in *chn‐1(by155)* and *ufd‐2(tm1380)* null allele worms and a decrease when CHN‐1 is overexpressed (Fig 5D). Quantitative PCR analyses showed no statistically significant changes in the AHCY‐1 transcript levels (Fig EV4J), suggesting that the increase in AHCY‐1 levels is posttranslationally regulated by CHN‐1 and UFD‐2.

Elevated homocysteine levels are linked to the deregulation of lipid metabolism and increased fat accumulation, apparent after RNA interference (RNAi) depletion of AHCY‐1 in worms (Vrablik *et al*, 2015; Visram *et al*, 2018). Using the lipophilic fluorophore RediStain WormDye Lipid Green to stain and quantify the fat content of *C. elegans*, we confirmed that AHCY‐1 depletion increases the abundance of lipids in wild‐type worms by almost 60%. Overexpression of *chn‐1* caused an increase in total lipid content to a level similar to that detected in *ahcy‐1* RNAi‐treated worms, and this effect was not further enhanced by AHCY‐1 depletion (Fig 5E). Interestingly, mutations in *chn‐1* and *ufd‐2* caused a reduction in the overall lipid levels and uncoupled the stimulation of lipid biogenesis induced by *ahcy‐1* RNAi (Fig 5E). Synthesis of phosphatidylcholine (PC) from phosphatidylethanolamine (PE) via the *de novo* phospholipid methylation pathway requires a significant amount of *S*‐adenosylmethionine (SAM) and is particularly sensitive to SAH levels (Tehlivets, 2011). Consistent with our assumption that deletion of either *chn‐1* or *ufd‐2* would positively affect AHCY‐1 stability, leading to intensification of SAM‐dependent methylation and PE to PC conversion, we noted that the ratio of PC to PE increased in *chn‐1(by155)* and *ufd‐2(tm1380)* worms (Fig 5F). In conclusion, our data suggest a functional role for the CHN‐1–UFD‐2 complex in AHCY‐1‐dependent lipid metabolism regulation.

## Discussion

The crystal structure of murine CHIP E3 bound to an HSP90 decapeptide containing the EEVD motif revealed an asymmetric dimerization in which the two CHIP protomers adopt different conformations. Given the limited conformational accessibility to E2 enzymes, we consider this a “closed” state, where only one of the U‐box domains in the dimer is accessible for E2 binding, and the TPR domain blocks the other (Zhang *et al*, 2005). In agreement with a computational model of human CHIP (Ye *et al*, 2017), our homology modeling of the CHN‐1 dimer suggested that it can take the form of both a metastable symmetric dimer (“open” state, representing unrestricted conformational accessibility to E2), in which both U‐box domains can simultaneously bind E2 enzymes and an asymmetric dimer with low ubiquitylation activity. We showed that the interaction of UFD‐2 with the CHN‐1 TPR domain reduces its dynamics, thus liberating its U‐box domains. In this steady‐state open conformation, CHN‐1 achieves high poly‐Ub activity due to the full functionality of the U‐box dimer. Consistently, we observed a twofold increase in the utilization of charged E2 by the CHN‐1‐UFD‐2^P951A^ complex compared with that of CHN‐1 alone. We also showed that not only poly‐Ub but also CHN‐1 mono‐Ub, which is the rate‐limiting step of ubiquitylation, is also enhanced upon UFD‐2 binding (Petroski & Deshaies, 2005). We also found that UFD‐2 is unaffected, unlike CHN‐1, in the complex and that CHN‐1 is not a substrate of UFD‐2. We believe that CHN‐1 undergoes different conformational flexibility upon binding to interaction partners, affecting its activity and providing a functional regulation layer.

The N‐terminal TPR domain of CHIP has been shown to interact specifically with the C‐terminal EEVD motif of HSP70 and HSP90 (Zhang *et al*, 2005; Xu *et al*, 2006; Graf *et al*, 2010). We discovered that UFD‐2 uses a slightly modified motif, that is, EEYD, to engage the CHN‐1 TPR domain. Furthermore, we demonstrated that only the presence of a UFD‐2 peptide containing the EEYD sequence was sufficient to promote CHN‐1 activity. By contrast, the *C. elegans* HSP70 homolog, HSP‐1, negatively regulates CHN‐1 and CHN‐1/UFD‐2 complex activity by promoting its “closed” state and preventing E2 discharge. The GHFDPVTR sequence in the U‐box domain is evolutionarily conserved in CHIP homologs from different species, but its role was not previously known. Here, we showed that CHN‐1 activity is negatively regulated by the interaction between positions associated with the EEVD motif of HSP‐1 and the conserved R230 position in the GHFDPVTR sequence. Through direct interactions with the CHN‐1 TPR and U‐box domains, HSP‐1 brings both regions into proximity, thereby impairing the U‐box dimer. This effect depends only on the local interaction of the HSP‐1 C‐terminus with the U‐box and not on steric hindrance induced by the entire chaperone that could limit E2 access to U‐box domains. In a co‐crystal structure with CHIP, HSP90 also forms hydrogen bonds (H‐bonds) between T and S in its C‐terminal peptide (TSRMEEVD) and the CHIP TPR domain (Zhang *et al*, 2005). The existence of these H‐bonds between the HSP‐1 peptide (GPTIEEVD) and CHN‐1 is not apparent. However, the HSP‐1 C‐terminal sequence is rich in glycines that could more efficiently tailor the binding by forming H‐bonds with the CHN‐1 backbone, possibly leading to a very close interaction. It has been shown that HSP70 can interact with the CHIP TPR domain through the conserved EEVCNPIITKLYQSAGGMP sequence (in addition to the EEVD motif) (Zhang *et al*, 2015). However, we did not find a similar bipartite interaction between UFD‐2 and CHN‐1. After many attempts, we were unable to obtain a co‐crystal of CHN‐1 with UFD‐2, and, thus, detailed insight into the organization of the complex remains enigmatic.

We observed that worm DAF‐21/HSP90 has a lower affinity for CHN‐1 and does not affect CHN‐1 activity, unlike HSP‐1/HSP70. Consistent with this observation, the C‐terminal HSP70 peptide blocks CHIP activity markedly greater than the HSP90 peptide, which binds to the CHIP TPR domain weaker than the HSP70 peptide (Narayan *et al*, 2015). The K30A missense mutation in CHIP, which likely mimics HSP70 binding, also reduces CHIP activity. Furthermore, HSP70 inhibits CHIP‐dependent ubiquitylation of folded substrates such as Smad1/5 (Wang *et al*, 2011), PPARγ2 (Kim *et al*, 2017), p53, or IRF‐1 (Narayan *et al*, 2015). It is noteworthy that HSP70 can stimulate BAG‐1 cochaperone ubiquitylation under experimental conditions that inhibit p53 and IRF‐1 modification; however, the increase in BAG‐1 modification was not accompanied by an increase in CHIP auto‐Ub, an indicator of its activity. Moreover, stimulation of BAG‐1 ubiquitylation was suppressed by the C‐terminal peptide of HSP70 (^634^GPTIEEVD^641^). Thus, the authors suggest that HSP70, through its direct interaction with BAG‐1, may facilitate its modification by CHIP. HSP70 can also exert different effects on CHIP‐dependent ubiquitylation of TP63 isoforms (a homolog of the p53 tumor suppressor), that is, it potentiates ubiquitylation of the TAp63 isoform and reduces modification of the ΔNp63 isoform (Wu *et al*, 2021). However, the authors did not present results on CHIP auto‐Ub or on the competition between TP63 isoforms and HSP70 for CHIP binding, which would allow a precise determination of the effect of HSP70 on CHIP processivity in their experimental system. Two different heat‐shock cognate protein 70 (HSC70, a member of the heat‐shock protein 70 family) cochaperones, BAG‐2 and HspBP1, limit CHIP activity (Alberti *et al*, 2004; Arndt *et al*, 2005; (Dai *et al*, 2005). BAG‐2 mediated inhibition is associated with reduced E2 accessibility, which is likely related to a shift favoring the CHIP “closed” state and stimulation of chaperone‐assisted CFTR maturation. Moreover, CHIP can enhance the ubiquitylation of Pael‐R (Parkin‐associated endothelin receptor‐like receptor) by Parkin E3 ligase, and this modification was inhibited by HSP70 (Imai *et al*, 2002). The *C. elegans* Parkin ortholog, PDR‐1, also interacts with CHN‐1, and both are expressed in neurons and body wall muscles. However, their mechanism of action and the substrates modified by the CHN‐1–PDR‐1 complex are obscure. In concert with our data, the above examples indicate an evolutionarily conserved role for HSP70/HSP‐1 as a negative regulator of CHIP/CHN‐1. We cannot exclude the possibility that posttranslational modifications of CHN‐1 or the presence of specific factors that would limit the interaction of HSP‐1 with the U‐box domains could sustain the ability of CHN‐1 to ubiquitylate chaperone‐bound substrates.

To propose a non‐quality‐control role for the CHN‐1–UFD‐2 pair, we wanted to determine the protein(s) regulated by these E3s. We identified AHCY‐1 as a novel substrate that undergoes ubiquitylation‐dependent turnover driven by the CHN‐1–UFD‐2 complex. AHCY‐1 is the only eukaryotic enzyme capable of hydrolyzing SAH, which is essential for SAM‐dependent methylation (Cantoni, 1975). According to the results of the yeast two‐hybrid screen (conducted by Hybrigenics), AHCY‐1 uses its substrate‐binding domain (amino acid residues 1–156) to interact with CHN‐1. This finding might suggest that CHN‐1 binding alone can regulate SAH processing by AHCY‐1, and we intend to investigate this possibility. PC synthesis from PE via phospholipid methylation involves a significant amount of SAM and is sensitive to SAH levels; thus, maintenance of this process requires AHCY‐1 (Tehlivets, 2011). Consistent with the regulatory effect of the CHN‐1–UFD‐2 pair on AHCY‐1, we observed an increase in PCs in worms lacking CHN‐1 or UFD‐2. To further understand the effect of CHN‐1–UFD‐2 on the SAM cycle, analysis of the levels of bound metabolites such as methionine, homocysteine, SAM, SAH, MTA, or GSH would be required. This metabolomic analysis might also help to explain the cause of the sensitivity of *chn‐1(by155)* worms to oxidative stress (Tawo *et al*, 2017). This sensitivity might be related to the deregulation of homocysteine formation due to the impaired control of AHCY‐1 as approximately 50% of the cysteine in glutathione, which plays a crucial role in cellular defence against reactive oxygen species, is derived from homocysteine (Vitvitsky *et al*, 2003). RNAi depletion of AHCY‐1 increases fat accumulation in worms (Vrablik *et al*, 2015; Visram *et al*, 2018 and our results). Interestingly, we found that *chn‐1* or *ufd‐2* knockout inhibits the lipid biogenesis induced by *ahcy‐1* RNAi. Perhaps, this effect is related to AHCY‐1 stabilization in tissues that are less sensitive to RNAi, meaning that its depletion was incomplete. Recent findings support the importance of CHIP in regulating the methylation status of the cellular proteome by mediating proteasomal turnover of the SAM‐dependent methyltransferases PRMT1, PRMT5, and EZH2 (Zhang *et al*, 2016; Bhuripanyo *et al*, 2018). However, further studies are necessary to delineate the involvement of the CHN‐1–UFD‐2 pair in modulating the cellular methylation potential. Additional CHN‐1–UFD‐2 substrates are likely to be present in our proteomic data set, although their confirmation requires detailed kinetic analyses.

CHN‐1–UFD‐2 assembly may be desirable by cells in certain contexts, such as efficiently loading a substrate for the proteasome. We observed that *in vitro*, the CHN‐1–UFD‐2 pair generates long Ub chains. Long Ub chains linked via Lys48 are resistant to multiple DUBs, which might promote proteasomal signaling of substrates by providing efficient targeting for degradation (Schaefer & Morgan, 2011). Furthermore, we showed that through interactions with UFD‐2, CHN‐1 could cooperate with various classes of E2 enzymes. We cannot exclude the possibility that in the complex, each ligase might be able to recruit different E2s, which could, in turn, enhance the generation of a mixed or branched type of Ub chains. In addition, Kuhlbrodt and coworkers suggested a link between the DUB enzyme ataxin‐3 and the CHN‐1–UFD‐2 complex, which could allow modulation of Ub chain signaling with various functional consequences for their targets (Kuhlbrodt *et al*, 2005). While our analyses do not provide a comprehensive view of the functions of the CHN‐1–UFD‐2 pair *in vivo*, they establish a starting point for elucidating the details of its regulation and cellular functions.

Based on our *in vitro* data, we hypothesized that interaction of CHN‐1 with UFD‐2 would simultaneously increase ubiquitylation of specific CHN‐1 targets and turnover of CHN‐1 *in vivo*, resulting from its increased auto‐Ub. Indeed, deletion of *ufd‐2* in worms led to increased CHN‐1 stability and protection against proteasomal degradation. Concerning the protein quality control role of CHN‐1/CHIP in *C. elegans*, this stabilization would increase the proteostasis capacity. We believe that a reduction in CHN‐1 processivity is probably desirable for HSP‐1 as it would not lead to an imbalance between chaperone‐mediated folding/maturation and degradation, inducing the latter. In summary, our data provide mechanistic insight into the distinct regulation of CHN‐1 activity by HSP‐1 and UFD‐2.

## Materials and Methods

### Reagents and Tools table

**Table:** 

Reagent/Resource	Source	Identifier
**Chemicals, peptides, and Recombinant proteins**		
UBE1	Boston Biochem	Cat. # E‐304
UBE2D1	Boston Biochem	Cat. # E2‐616
GST UbcH5a/UBE2D1	Boston Biochem	Cat. # E2‐615
UBE2D2	Boston Biochem	Cat. # E2‐622
UBE2D3	Boston Biochem	Cat. # E2‐627
UBE2N/Uev1a	Boston Biochem	Cat. # E2‐664
UBE2W	Boston Biochem	Cat. # E2‐740
10X E3 Ligase Reaction Buffer	Boston Biochem	Cat. # B‐71
10X Ubiquitin conjugation Reaction Buffer	Boston Biochem	Cat. # B‐70
10X Energy Regeneration Solution	Boston Biochem	Cat. # B‐10
Ubiquitin	Boston Biochem	Cat. # U‐100H
FLAG (DYKDDDDK)‐Ubiquitin	Boston Biochem	Cat. # U‐120
UbNoK	Boston Biochem	Cat. # UM‐NOK
Ub3KTR	Boston Biochem	Cat. # UM‐3KTR
M1linked‐ linear ubiquitin	UbiQ	Cat. # UbiQ‐L01
UBE2D1 ubiquitin charged	Boston Biochem	Cat. # E2‐800
N‐Ethylmaleimide (NEM)	Sigma‐Aldrich	Cat. # E3876
cOmplete™, EDTA‐free Protease Inhibitor Cocktail	Roche	Cat. # 11873580001
MG132	Selleckchem	Cat. # S2619
Apyrase	Sigma‐Aldrich	Cat. # A2230
Alkaline Phosphatase Yellow (pNPP) Liquid Substrate	Sigma‐Aldrich	Cat. # P7998
Q5 Site‐Directed Mutagenesis Kit	NEB	Cat. # E0552S
4x Laemmli Sample Buffer	Bio‐Rad	Cat. # 1610747
β‐Mercaptoethanol	Sigma‐Aldrich	Cat. # M6250
Dynabeads™ Co‐Immunoprecipitation Kit	Invitrogen	Cat. # 14321D
Pierce™ Anti‐DYKDDDDK Magnetic Agarose	Invitrogen	Cat. # A36797
RediStain™ WormDye Lipid Green	NemaMetrix	Cat. # DYE9439
Rapid Gold BCA Protein Assay Kit	Thermo Fisher Scientific	Cat. # A53225
IMPACT™ Kit	NEB	Cat. # E6901S
AHCY‐1::6xHis	This paper	
CHN‐1	This paper	
6xHis::SUMO::CHN‐1	This paper	
CHN‐1^Δ110^	This paper	
CHN‐1^Δ87^	This paper	
CHN‐1^Δ95^	This paper	
CHN‐1^R230A^	This paper	
UFD‐2	This paper	
UFD‐2^EEVD^	This paper	
6xHis::UFD‐2	This paper	
6xHis::UFD‐2^P951A^	Ackermann *et al* (2016)	
6xHis::UFD‐2^ΔUbox^	This paper	
6xHis::Ufd2p	This paper	
HSP‐1::6xHis	This paper	
6xHis::DAF‐21	This paper	
HSP‐1^ΔEEVD^::6xHis	This paper	
6xHis::DAF‐21^ΔEEVD^	This paper	
HSP‐1^EEYD^::6xHis	This paper	
**Antibodies**		
Anti‐CHN‐1 antibody	Tawo *et al* (2017)	
Anti‐UFD‐2 antibody	Ackermann *et al* (2016)	
Anti‐AHCY‐1 antibody	This study	
Anti‐Ubiquitin antibody	Cell signaling Technology	Cat. # 3936s
Anti‐Histidine antibody	Santa Cruz Biotechnology, Inc.	Cat. # SC‐53073
Anti‐GST antibody	Sigma‐Aldrich	Cat. # G1160
Anti‐UBE2D1 antibody	Biorad	Cat. # VPA00296
**Bacterial Strains**		
*E. coli* RNAi feeding strain	Caenorhabditis Genetics Center	HT115(DE3)
*E. coli* feeding strain	Caenorhabditis Genetics Center	OP50
Ahringer RNAi library	Source BioScience	*C. elegans* RNAi Collection (Ahringer)
Rosetta™ 2 (DE3)	Novagen	Cat. # 71400
BL21 Star™ (DE3)	Thermo Fisher Scientific	Cat. # C601003
Top10	Thermo Fisher Scientific	Cat. # C4040
**Oligonucleotides**		
pTYB21‐MBP::Intein‐UFD‐2 Forward: GGTGGTTGCTCTTCCAACATGATTGAAGACGAGAAAGCAGG Reverse: GGTGGTCTGCAGTCATTATTTCTTTGAATTTCTTT	This paper	
pET‐6xHis::SUMO‐CHN‐1^Δ110^ Forward: ATTGAGAACGCCCTCAAAC Reverse: GCTAGCTAGACCACCAATC	This paper	
pET‐6xHis::SUMO‐CHN‐1^Δ87^ Forward: TACAGTGAAGCAATAAGCTG Reverse: GCTAGCTAGACCACCAATC	This paper	
pET‐6xHis::SUMO‐CHN‐1^Δ95^ Forward: TCCAAAGCGCTCTACCAT Reverse: GCTAGCTAGACCACCAATC	This paper	
pET‐21a‐VSV‐HSP‐1^ΔEEVD^::6xHis Forward: GCGGCCGCACTCGAG Reverse: TCCTCCGGCGGCTCCTCC	This paper	
pET‐21a‐6xHis::DAF‐21^ΔEEVD^ Forward: TAATGAGGATCCGAATTCGAG Reverse: CTCAGCTCCCTCAATCTT	This paper	
pET‐6xHis::SUMO‐CHN‐1^R230A^ Forward: TCCAGTCACAGCAAAACCACTTAC Reverse: TCGAAATGGCCAATTCTTC	This paper	
pTYB21‐MBP::Intein‐UFD‐2 ^Y907V(EEVD)^ Forward: GAAGAAGAGGTTGATGATGTACCA Reverse: TGGTACATCATCAACCTCTTCTTC	This paper	
pET‐21a‐VSV‐HSP‐1 ^V639Y(EEYD)^ Forward: ATCGAGGAGTACGACGCGGCC Reverse: GGCCGCGTCGTACTCCTCGAT	This paper	
**Recombiant DNA**		
pET28a‐6xHis::Ufd2p	Liu *et al* (2017)	
pET‐6xHis::SUMO::CHN‐1	This paper	
pET28a‐6xHis::UFD‐2	This paper	
pLATE31‐AHCY‐1::6xHis	This paper	
pET21a‐VSV::HSP‐1::6xHis	This paper	
pET21a‐6xHis::DAF‐21	This paper	
pTYB21‐MBP::Intein‐UFD‐2	This paper	
pET‐6xHis::SUMO‐CHN‐1^Δ110^	This paper	
pET‐6xHis::SUMO‐CHN‐1^Δ87^	This paper	
pET‐6xHis::SUMO‐CHN‐1^Δ95^	This paper	
pET‐21a‐VSV‐HSP‐1^EEYD^::6xHis	This paper	
pET‐21a‐VSV‐HSP‐1^ΔEEVD^::6xHis	This paper	
pET‐21a‐6xHis::DAF‐21^ΔEEVD^	This paper	
pET‐6xHis::SUMO‐CHN‐1^R230A^	This paper	
pTYB21‐MBP::Intein‐UFD‐2 ^Y907V(EEVD)^	This paper	
pET‐21a‐VSV‐HSP‐1 ^V639Y(EEYD)^	This paper	
**Experimental models: Organisms/Strains**		
*C. elegans*: Bristol (N2) strain as wild‐type	CGC	N/A
*C. elegans*: chn‐1(by155)I	CGC	WormBase ID: WBVar00000641
*C. elegans*: unc‐119(ed4)III; hhIs136[unc‐119(+); chn‐1p::chn‐1::FLAG]	Tawo *et al* (2017)	N/A
*C. elegans*: ufd‐2(tm1380)II	CGC	WormBase ID: WBVar00250374
*C. elegans*: chn‐1(by155)I; ufd‐2(tm1380) II	This paper	N/A
*C. elegans*: chn‐1(by155)I; ahcy‐1(syb646[ahcy‐1::GFP]I	This paper	N/A
*C. elegans*: ahcy‐1(syb646[ahcy‐1::GFP]I; ufd‐2(tm1380)II;	This paper	N/A
*C. elegans*: unc‐119(ed4)III; hhIs136[unc‐119(+); chn‐1p::chn‐1::FLAG]; ahcy‐1(syb646[ahcy‐1::GFP]I	This paper	N/A
**Software and Algorithms**		
Graph Pad Prism	Graph Pad Software, Inc.	www.graphpad.com
Image Lab™ Version 6.0.0 build 25	Bio‐Rad Laboratories, Inc.	www.bio‐rad.com/de‐de/product/image‐lab‐software?ID=KRE6P5E8Z
ImageJ 1.53c	Wayne Rasband, NIH, USA	www.imagej.nih.gov/ij
**Materials**		
Nunc MaxiSorp™ flat‐bottom	Thermo Fisher Scientific	Cat. # 44‐2404
Hiload 16/600 Superdex S200	GE Healthcare	Cat. # GE28‐9893‐35

### Methods and Protocols

#### *C. elegans* strains

Worms were maintained on nematode growth medium (NGM) plates seeded with OP50 *Escherichia coli* bacteria at 20°C unless otherwise stated (Brenner, 1974). The following strains were used in this study: Bristol (N2) as wild‐type strain, *chn‐1(by155)I*, *ufd‐2(tm1380)II, chn‐1(by155)I*; *ufd‐2(tm1380)II, unc‐119(ed4)III*; *hhIs136[unc‐119(+), chn‐1p::chn‐1::FLAG]* (Tawo *et al*, 2017). These strains were at least six times outcrossed against the wild‐type strain to provide isogenic conditions. Strain generated in this study—PHX646 (ahcy‐1(syb646[ahcy‐1::GFP]I) and PHX792 (ufd‐2(syb792[ufd‐2^P951A^]II) were generated by SunyBiotech using CRISPR services (http://www.sunybiotech.com). PHX646 was outcrossed 2X to N2 to generate strain WOP122. PHX792 was outcrossed 2X to N2 to generate strain WOP160.

#### Generation of recombinant proteins

All recombinant proteins were produced using a bacterial expression system. CHN‐1 and the CHN‐1 variants were expressed and purified from Rosetta™ 2 (DE3) cells. UFD‐2, HSP‐1, DAF‐21, and their variants were expressed and purified from BL21 Star™ (DE3) cells. Truncations and point mutations in the protein constructs were introduced using the Q5 Site‐Directed Mutagenesis Kit (New England Biolabs; NEB). Protein over‐expression was induced using 0.4 mM IPTG at 22°C for 16 h. Respective induced cell pellets were harvested via centrifugation at 4,000 rpm for 20 min at 4°C. Cells were lysed in a lysis buffer (20 mM HEPES pH 8, NaCl 300 mM, 2 mM BME, protease inhibitor, and DNase) by sonication. After sonication, the supernatant and pellet fractions were separated via high‐speed centrifugation at 14,000 rpm for 1 h at 4°C. Tagged proteins were purified from the soluble fraction of the cell lysates using appropriate Ni‐NTA or GST‐trap columns or chitin beads (NEB). After removing the affinity tags, affinity‐purified protein fractions were subjected to gel filtration chromatography (Hiload 16/600 Superdex S200, GE Healthcare) to obtain more than 95% pure protein fractions for use in subsequent biophysical and biochemical experiments. For the *in vitro* ubiquitylation reactions, we first generated a pTYB21‐UFD‐2 expression vector and purified tagless UFD‐2 fraction using the intein cleavage site as per the manufacturer's protocol (NEB). The lysis buffer used for purifying this variant contained HEPES 20 mM, TritonX 0.1%, 5% glycerol, 500 mM NaCl, pH 8.0. To generate tagless CHN‐1 and His‐tagged CHN‐1, we affinity‐purified the proteins using Ni‐NTA columns. Furthermore, His‐SUMO tag were cleaved using SUMO protease treatment (16 h) at 4°C, and untagged CHN‐1 dimeric fraction was purified via SEC.

#### Peptide microarray for protein‐peptide interaction studies

This assay was performed by PEPperPRINT GmbH (https://www.pepperprint.com/). Briefly, the UFD‐2 sequences were elongated with neutral GSGSGSG linkers on the C‐ and N‐termini to avoid truncated peptides and translated into 7 and 13 amino acid peptides with overlaps of 6 and 12 amino acids. The resulting UFD‐2 peptide microarrays (containing 1,986 different peptides) were printed in duplicate and incubated with recombinant 6xHis::SUMO::CHN‐1 for 16 h at 4°C. After washing, the microarrays were incubated with mouse anti‐6x‐His Epitope Tag DyLight680 secondary antibodies to detect bound 6XHis::SUMO::CHN‐1.

#### Ubiquitylation assays

*In vitro* assays were performed according to an earlier protocol (Hellerschmied *et al*, 2018). The reactions were run at 30°C for 90 min using 60 μM ubiquitin and its variants (Boston Biochem) in the presence of 100 nM E1 (UBE1, Boston Biochem), 0.6 μM E2 (Boston Biochem), E3 ligase (CHN‐1 and variants or UFD‐2 and variants), E3 ligase reaction buffer (Boston Biochem), and Energy Regeneration Solution (Boston Biochem). For performing the *in vitro* reaction in the presence of both the CHN‐1 and UFD‐2 or His‐tagged UFD‐2^P951A^, proteins were first preincubated at 16°C for 30 min in the presence of E3 ligase reaction buffer. After that, the remaining reagents were added for the ubiquitylation reaction and incubated at 30°C for the indicated time. For substrate ubiquitylation, *C. elegans* AHCY‐1 was added as the substrate along with the other reagents and mixed with preincubated CHN‐1 or pre‐incubated CHN‐1/UFD‐2 and incubated at 30°C for 90 min. For performing the *in vitro* reaction in the presence of a chaperone, *C. elegans* 1 μM His‐tagged HSP‐1, His‐tagged DAF‐21, or other variants were preincubated with CHN‐1 or CHN‐1/UFD‐2 at 16°C for 30 min in the presence of 1× E3 ligase reaction buffer. After that, the remaining reagents were added for the reaction and incubated at 30°C for 90 min. Reaction stopped by adding Laemmli sample buffer (Bio‐Rad), including β‐mercaptoethanol (Sigma‐Aldrich), and incubated at 95°C for 5 min. Samples were run in 12% SDS–PAGE gels and blotted with an antibody against the protein of interest.

#### E2 discharging assays

E2 discharging experimental protocol was designed based on a modified method from Page *et al*, 2012. Discharging of increasing molar concentration of charged UBE2D1 (UBE2D1‐Ub, Boston Biochem) was performed at 30°C for 40 min in ubiquitin conjugation reaction buffer (Boston Biochem, Cat#B‐70). Similarly, a time‐dependent assay was performed using 3.3 μM UBE2D1‐Ub at 30°C for different time points (5, 10, 20, 30 min) with equimolar concentrations (1 μM) of CHN‐1, His‐tagged UFD‐2^P951A^ and His‐tagged HSP‐1. The reaction was stopped by the addition of Laemmli sample buffer (Bio‐Rad). As a control experiment to check the total UBE2D1 used in the reaction, the charged UBE2D1 were treated with 50 mM DTT and boiled the sample at 90°C for 5 min to obtain the total uncharged E2. Samples were run in a 15% SDS–PAGE gel. For detecting the available UBE2D1‐Ub in each condition, Western blotting was performed using an anti‐ubiquitin or anti‐UBE2D1 antibody. Normalized chemiluminescence intensity was obtained after maximum background subtraction from each lane. The discharging capacity at a specific condition was analyzed by detecting the band intensity of the uncharged fraction of UBE2D1 while probing with an anti‐UBE2D1 antibody or available charged UBE2D1 while probing with an anti‐ubiquitin antibody at the end of the reaction.

#### Generation of ubiquitin‐charged GST‐tagged UbcH5a/UBE2D1

1.25 μM E2 GST::UbcH5a/UBE2D1 (Boston Biochem) was charged using 58 μM ubiquitin (Boston Biochem), 0.25 μM E1 (UBE1, Boston Biochem), ATP and ubiquitin‐conjugating buffer (Boston Biochem), and incubated at 30°C for 30 min. Charging reaction was stopped by adding 5 mM Ayrase (Sigma‐Aldrich). The reaction mix was used as a source of UBE2D1^GST^‐Ub.

#### E3‐ubiquitin stoichiometry analysis using UBE2W ubiquitylation assay

0.6 μM E2 UBE2W (Boston Biochem) was charged using 60 μM ubiquitin (Boston Biochem) or 60 μM FLAG‐Ubiquitin (Boston Biochem) in the presence of ATP, ubiquitin‐conjugating buffer (Boston Biochem) and incubated at 30°C for 30 min. After that, the conjugation reaction was stopped by adding 5 mM Ayrase (Sigma‐Aldrich). Then, reaction mix containing UBE2W‐Ub or UBE2W‐Ub^FLAG^ was used in a discharging assay bin at 30°C for 40 min. The reaction was stopped by the addition of Laemmli sample buffer (Bio‐Rad) including β‐mercaptoethanol (Sigma‐Aldrich), and boiled for 5 min. Samples were run in 12% SDS–PAGE gels and blotted with an antibody against the protein of interest.

#### Western blotting and quantification

Protein samples in SDS‐loading dye (reducing/non‐reducing) were run in 12 or 15% acrylamide gels using running buffer (25 mM Tris, 190 mM glycine, 0.1% SDS) at 120 volts (constant). The wet transfer was made at a constant 200 mA for 2 h at room temperature using transfer buffer (25 mM Tris, 190 mM glycine, 10% methanol, pH 8.3). Blots were blocked with 5% skimmed milk in TBST (50 mM Tris, 150 mM NaCl, 0.1% Tween 20, pH 7.5) for 1 h at room temperature and incubated overnight with primary antibody prepared in 5% skimmed milk in TBST at 4°C. The blots were then washed three times with TBST for 10 min. Finally, the blots were incubated with secondary antibodies prepared in 5% skimmed milk in TBST for 1 h at room temperature. Imaging was performed using a ChemiDoc™ Imaging System (Bio‐Rad). All antibodies used in this study are listed in the resource table. Image Lab™ (version 6.0.0 build 25) software was used for quantification. After probing with a particular antibody, first, the lane marked the bands that appeared were marked in high sensitivity mode and quantified. Normalized chemiluminescence intensities were determined after maximum background subtraction from each lane. The graphs were plotted using GraphPad Prism 9.

#### Enzyme‐linked immunosorbent assay (ELISA)

2 μg/ml of UFD‐2, His‐tagged DAF‐21, and His‐tagged HSP‐1 in coating buffer (100 mM NaHCO_3_, 32 mM Na_2_CO_3_, pH 9.2) were immobilized on Nunc‐Immuno plates for ELISA (Thermo Fisher Scientific) overnight at 4°C. Blocking was performed with 2% BSA for 1 h at 25°C, followed by washing with TBST (0.1% Tween 20). After incubation with increasing CHN‐1 concentrations for 1 h at 16°C, unbound CHN‐1 was washed away by subsequent TBST washing steps. Interacting proteins were detected using an antibody against CHN‐1 (1:5,000 dilution, overnight 4°C), followed by TBST washing and the addition of an HRP‐conjugated secondary antibody. After the final wash, 100 μl of pnPP substrate (Alkaline Phosphatase Yellow, Sigma‐Aldrich) was added in the dark. After 15 min, the reaction was stopped by adding 50 μl of 3 M NaOH, and the absorbance was measured at 450 nm.

#### Microscopy of worms

Day 0 adult worms maintained at 20°C were immobilized with tetramisole and immediately imaged using Nikon SMZ25 microscope. Data analysis: Image processing was performed with ImageJ (Fiji) using Binary Mask and Particle Analysis Procedure with background signal subtraction.

#### qPCR

RNA extraction, genomic DNA digestion at the young adult (adult day 0‐1) stage was performed as described in Ly *et al*, 2015, with the exception that min. A total of 100 worms were used instead of a single worm per sample as in the publication, cDNA synthesis was performed using the Maxima H Minus cDNA synthesis kit (Thermo Fisher). RT‐PCR was performed with SG qPCR Master Mix (EURx), according to the manufacturer's protocol. Equipment used—Light Cycler 96. Data analyzed as the ratio between CT candidate gene (*ahcy‐1*) averaged from 3 replicates/CT ref (actin coding gene *act‐1* as a reference).

#### Modeling and molecular dynamics

CHN‐1 model was generated by homology modeling using the SWISS‐MODEL web server (Waterhouse *et al*, 2018) with PDB ID 2F42 and 2C2L as the templates. The primary sequence of peptides used for docking on the CHN‐1 dimer model was 628–640 HSP‐1 (P09446) and 894–911 UFD‐2 (Q09349). The protein and peptide complex structures were subjected to an energy minimization strategy using pmem.cuda (Goetz *et al*, 2012; Salomon‐Ferrer *et al*, 2013) from the AMBER18 package (Case *et al*, 2018). tLeap binary (part of AMBER18) was used for solvating the structures in an octahedral TIP3P water box with 15 Å distance from the structure surface to the box edges; closeness parameter was set to 0.75 Å. The system was neutralized and solvated in a solution of 150 mM NaCl. Simulations were carried out by equilibrating the system for 1 ns (NPT) at 1 atm in 300K, followed by 10 ns NPT for nonbonded interaction using the AMBER ff14SB force field (Maier *et al*, 2015). The particle mesh Ewald (PME) method was used to treat the long‐range electrostatic interactions; hydrogen bonds were constrained using SHAKE algorithm and integration time‐step at 2 fs. (Ryckaert *et al*, 1977).

#### Hydrogen deuterium exchange mass spectrometry (HDX‐MS)

Prior to HDX‐MS reactions, a complex of CHN‐1 (3 mg/ml) and His‐tagged UFD‐2 (2 mg/ml) was formed by mixing the proteins in a 1:1 molar ratio followed by incubation at 25°C for 30 min. HDX‐MS of CHN‐1 and CHN‐1 in complex with UFD‐2 were performed at five time points during the incubation with deuterium (10 s, 1 min, 5 min, 25 min, and 2 h) in triplicate. 5 μl aliquots of proteins were added to 45 μl of deuterated buffer (10 mM HEPES, 150 mM NaCl in 99.99% D_2_O, pH = 8.0) at room temperature. The exchange reaction was quenched by moving the exchange aliquots to precooled tubes (on ice) containing 10 μl of quenching buffer (2 M glycine, 4 M guanidine hydrochloride, 100 mM TCEP in 99.99% D_2_O, pH 2.3). After quenching, samples were frozen immediately in liquid nitrogen and kept at ‐80°C until mass spectrometry measurement. Samples were thawed directly before measurement and injected manually onto the nano ACQUITY UPLC system equipped with HDX‐MS Manager (Waters). Proteins were digested on 2.1 mm × 20 mm columns with immobilized Nepenthesin‐2 (AffiPro), for 1.5 min at 20°C and eluted with 0.07% formic acid in water at a flow rate of 200 μl/min. Digested peptides were passed directly to the ACQUITY BEH C18 VanGuard pre‐column from which they were eluted onto the reversed‐phase ACQUITY UPLC BEH C18 column (Waters) using a 6–40% gradient of acetonitrile in 0.01% of formic acid at a flow rate of 90 μl/min at 0.5°C. Samples were measured on the SYNAPTG2 HDX‐MS instrument (Waters) in IMS mode. The instrument parameters for MS detection were as follows: ESI—positive mode; capillary voltage—3 kV; sampling cone voltage—35 V; extraction cone voltage—3 V; source temperature—80°C; desolvation temperature—175°C; and desolvation gas flow 800 l/h. The CHN‐1 peptide list was obtained using non‐deuterated protein samples, processed as described above for HDX experiments, and measured in Mse mode. Peptides were identified using ProteinLynx Global Server Software (Waters). The HDX‐MS experiment was analyzed using DynamX 3.0 (Waters) software. The PLGS peptide list was filtered by minimum intensity criteria—3000 and minimal product per amino acid—0.3. All MS spectra were inspected manually. Final data analysis was carried out using the in‐house HaDeX software (Puchała *et al*, 2020). Differential deuterium exchange of residues was mapped to the model of CHN‐1 generated using the 2C2L CHIP structure in the SWISS‐MODEL Web server (https://swissmodel.expasy.org/). The uncertainty measurement was calculated using the uncertainty transfer law consistent with ISO standards and community guidelines.

The exact process is described in our documentation: https://hadexversum.github.io/HaDeX/articles/datafiles.html.

#### Surface plasmon resonance (SPR)

SPR‐based interaction analysis was carried out at 25°C on a Biacore S200 instrument (GE Healthcare). Recombinant purified His‐tagged UFD‐2 and His‐tagged Ufd2p proteins were immobilized on NTA Biacore sensor Chips (Series S) at 20 μg/ml. Single‐cycle kinetics studies were performed by passing increasing concentrations (0, 100, 200, 500, 1,000, and 2,000 nM) of analyte M1 diUb conjugates (UbiQ) in SPR buffer (10 mM HEPES, 150 mM NaCl, 0.05% Tween 20, 0.1% BSA, 50 μM EDTA, pH 8.0). The runs for both proteins were carried out under identical conditions. All injections were compiled in the same sensorgram with the response unit (RU) on Y‐axis versus time (sec) on the X‐axis.

#### Preparation of *C. elegans* lysates and co‐immunoprecipitation

Worms were grown at 20°C. For protein extraction, worms were collected in M9 buffer and lysed using a lysis buffer (50 mM KCl, 10 mM Tris‐HCl pH 8.2, 2.5 mM MgCl_2_, 0.07% NP‐40, 0.7% Tween‐20, 0.1% gelatine) with protease inhibitor (Roche) and in the presence of DUB inhibitor, NEM (Sigma‐Aldrich). First, worms in lysis buffer were snap‐frozen in liquid nitrogen. Next, the frozen samples were sonicated (40% amplitude, 5 cycles of 30 s pulses at 30 s intervals, Vibra‐Cell™) on ice. Samples were centrifuged at 13,000 rpm for 15 min and the supernatants were collected. For co‐immunoprecipitation, anti‐DYKDDDDK (FLAG tag) magnetic beads (Anti‐DYKDDDDK Magnetic Agarose, Pierce) were used. 50 μl of anti‐DYKDDDDK magnetic beads slurry were used for 200 μl of worm lysate. Lysate of CHN‐1::FLAG‐expressing worms was used as the experimental sample and wild‐type (N2) worms were used as a negative control. Worm lysates were incubated with equilibrated magnetic beads at 4°C for 1 and 2 h for UFD‐2 and AHCY‐1 pull down, respectively. After the desired incubations, the beads were washed three times using washing buffer (PBS with 100 mM NaCl). Samples were eluted via the addition of Laemmli sample buffer (Bio‐Rad) containing β‐mercaptoethanol (Sigma‐Aldrich), and boiled for 5 min.

#### RNA interference (RNAi)

RNAi was performed using the standard RNAi feeding method and RNAi clones (Kamath & Ahringer, 2003). NGM plates supplemented with 1 mM IPTG and 25 μg/μl carbenicillin were seeded with HT115 *E. coli* expressing double‐stranded RNA (dsRNA) against the gene of interest or, as a control, bacteria with the empty vector were used. Worms were placed on freshly prepared RNAi plates as age‐synchronized L1 larvae.

#### TCA precipitation

Approximately 1,000 young adult worms were broken by sonication in 50% Tris‐sodium dodecyl sulfate buffer (25 mM Tris, 250 mM NaCl, 5% sodium dodecyl sulfate, pH 7.4), and the debris was pelleted by centrifugation for 5 min at 20,000 rcf. To precipitate proteins in the supernatant, trichloroacetic acid (TCA, final concentration 9.3%) was added to the supernatant and incubated at room temperature for 1 h. The supernatant was removed, leaving the protein pellet intact. Pellet was washed twice with 200 μl of cold acetone and centrifuged at 14,000 rpm for 5 min. Next, the pellet was dried by placing the tube in a 95°C heat block for 5–10 min to evaporate the acetone. The protein precipitate (TCA‐insoluble fraction) was dissolved in 150 μl of 350 mM NaOH for 1 h at room temperature. Total protein concentration was determined using the Rapid Gold BCA Protein Assay (Thermo Scientific).

#### Heat stress recovery

Approximately 50 young adult worms were washed from NGM plates and rinsed three times with M9 buffer. The worms were further suspended in a 1 ml M9 buffer. The worms to be heat stressed were incubated at 33°C for 60 min, whereas the control animals were incubated at 20°C for 60 min. Next, worm movement was recorded for 2 min using the WormLab system (MBF Bioscience). The frame rate, exposure time, and gain were set to 7.5 frames per second, 0.0031 s, and 1, respectively. The distance the worms travelled before and after heat stress was analyzed using the WormLab software (MBF Bioscience).

#### Proteomics

For proteomic analysis, the following young adult strains were utilized: N2, *ufd‐2(tm1380)*, *chn‐1(by155)* and *ufd‐2(tm1380); chn‐1(by155)*. Protein digestion: 4% SDS in 100 mM HEPES pH = 8.5 was used for lysis, and the protein concentrations were determined. 50 μg of protein was subjected to tryptic digestion. Proteins were reduced (10 mM TCEP) and alkylated (20 mM CAA) in the dark for 45 min at 45°C. Samples were subjected to SP3‐based digestion (Hughes *et al*, 2014). Washed SP3 beads (SP3 beads (Sera‐Magcan) Magnetic Carboxylate Modified Particles (Hydrophobic), and Seracan (TM) Magnetic Carboxylate Modified Particles (Hydrophilic)) were mixed equally, and 3 μl of beads were added to each sample. Acetonitrile was added to a final concentration of 50%, and the samples were washed twice using 70% ethanol (200 μl) on an in‐house‐made magnet. After an additional acetonitrile wash (200 μl), 5 μl of digestion solution (10 mM HEPES pH 8.5 containing 0.5 μg Trypsin (Sigma‐Aldrich) and 0.5 μg LysC (Wako) was added to each sample and incubated overnight at 37°C. Peptides were cleaned on a magnet using 2 × 200 μl acetonitrile washes and eluted in 10 μl of 5% DMSO in an ultrasonic bath for 10 min. Formic acid and acetonitrile were added to final concentrations of 2.5% and 2%, respectively. Samples were frozen until LC‐MS/MS analysis. Liquid chromatography and mass spectrometry: LC‐MS/MS instrumentation consisted of a nLC 1200 coupled to a nanoelectrospray source to a QExactive HF‐x (Thermo Fisher Scientific) mass spectrometer. Peptide separation was performed on an in‐house‐packed column (75 μm inner diameter, 360 μm outer diameter), and the column temperature was maintained at 50°C using a column oven (PRSO‐V2). The LC buffer system consisted out of 0.1% formic acid (A) and 0.1% formic acid in 80% acetonitrile (B). Peptides were separated using a 90 min gradient applying a linear gradient for 70 min from 7 to 29 % B and then ramped to 65% B within 10 min, followed by a linear increase to 95% B within 5 min. 95% B was held for 5 min. Before each run, the column was re‐equilibrated to 0% B. The mass spectrometer operated in a data‐dependent acquisition mode targeting the top 22 peaks for collision‐induced fragmentation and MS2 spectra acquisition. MS1 spectra were acquired in a scan range from 350 to 1,650 *m/z* allowing a maximum injection time of 20 ms for an AGC target of 3e6. Spectra were acquired at a resolution of 60,000 (at 200 *m/z*). Ions were isolated in an isolation window of 1.3 *m/z* using an AGC target of 1e6 and a canaximum injection time of 22 ms. Spectra were acquired at a resolution of 15,000. The scan range for the MS2 spectra was set to 200–2,000 *m/z*. The normalized collision energy was 28. Dynamic exclusion was set to 20 s. Data analysis: Acquired raw files were correlated to the Uniprot reference *C. elegans* proteome using MaxQuant (1.5.3.8) (Cox & Mann, 2008) and the implemented Andromeda search engine (Cox *et al*, 2011). Label‐free quantification and matching between runs were enabled using default settings. Carbamidomethylation of cysteine residues was set as a fixed modification. Oxidation of methionine residues and acetylation of protein N‐termini were defined as variable modifications. The false discovery rate (FDR) was controlled using the implemented revert algorithm to 1% at the protein and the peptide‐spectrum match (PSM). To identify significantly changed proteins, we performed a one‐way analysis of variance (ANOVA), correcting for multiple testing using a permutation‐based approach (FDR < 0.05, number of permutations: 500).

#### Lipidomics

The following young adult strains were utilized for lipidomic analysis: N2 (wild‐type), *ufd‐2(tm1380)*, and *chn‐1(by155)*. Lipid extraction: Lipids from a homogenized sample comprising 15 000 worms were extracted using the Folch method as follows: 200 μl of methanol was added to each sample, followed by 10 s of vortexing. Next, 500 μl of chloroform was added, followed by 10 s vortexing. This was followed by the addition of 200 μl of water to each sample to induce phase separation, followed by vortexing for 20 s. The samples were then kept in the cold for 10 min and centrifuged at 14,500 rpm for the next 10 min. The bottom layer was then pipetted out, and the solvent was dried under a stream of nitrogen. The lipid extract was reconstituted in 200 μl of 1:1 isopropanol:methanol solution before LC‐MS analysis. LC‐MS analysis: LC‐MS analysis was performed as previously described (Alseekh *et al*, 2021). Briefly, lipid extracts were separated on a Kinetex C18 2.1 × 100 mm, 2.6 μm column (Phenomonex, Aschaffenburg, De). Separation was achieved via gradient elution in a binary solvent, Vanquish UHPLC (Thermo Scientific, Bremen, DE). Mobile Phase A consisted of ACN:H_2_O (60:40), while mobile phase B consisted of IPA:ACN (90:10). For positive ionization, the mobile phases were modified with 10 mM ammonium formate and 0.1% formic acid, while for the negative ionization mode, the mobile phases were modified with 5 mM ammonium acetate and 0.1% acetic acid. A flow rate of 260 μl/min was used for separation, and the column and sample tray were held constant at 30°C and 4°C, respectively. 2 μl of each sample was injected onto the LC column. MS Instrumentation: MS analysis was performed on a Q‐Exactive Plus Mass Spectrometer (Thermo Scientific, Bremen, DE) equipped with a heated electrospray ionization probe. In both the positive and the negative ionization modes, the S‐Lens RF level was set to 65, and the capillary temperature was set to 320°C, and the sheath gas flow was set to 30 units and the auxiliary gas was set to five units. The spray voltage was set to 3.5 kV in the negative ionization mode and 4.5 kV in the positive ionization mode. In both modes, full scan mass spectra (scan range *m/z* 100–1,500, R = 35K) were acquired along with data‐dependent (DDA) MS/MS spectra of the five most abundant ions. DDA MS/MS spectra were acquired using normalized collision energies of 30, 40, and 50 units (R = 17.5K and an isolation width = 1 *m/z*). The instrument was controlled using Xcalibur (version 4.0). Data analysis and lipid annotation: Progenesis Q1, version 2.0 (Non‐Linear Dynamics, A Waters Company, Newcastle upon Tyne, UK) was used for peak picking and chromatographic alignment of all samples, with a pooled sample used as a reference. Lipids were annotated using the Progenesis Metascope Basic Lipids the LipidBlast databases with consideration made only of compounds that had MS/MS data. In both databases, the precursor ion tolerance was set to 10 ppm, and the fragmentation ion tolerance was set to 15 ppm. Putative lipid identifications were based on manual curation of database matches with fragmentation scores > 10%.

#### Fluorescent labeling of lipids in *C. elegans*

Lipid content in young adult worms was determined by RediStain WormDye Lipid Green (NemaMetrix) staining according to the manufacturer's protocol (incubating worms with the reagent for 30 min at room temperature with shaking). Working dye concentration: 1 μl of dye/200 μl of M9 buffer. Worms were protected from light, and several washes in M9 buffer were performed after staining. Subsequently, after immobilization of the worms with tetramisole, imaging was performed on a Nikon SMZ25 microscope. Data analysis: Image processing was performed with ImageJ (Fiji) using Binary Mask and Particle Analysis Procedure with background signal subtraction.

#### Yeast two‐hybrid screening

Yeast two‐hybrid screening was performed by the Hybrigenics Services (http://www.hybrigenics‐services.com). The coding sequence for *C. elegans* CHN‐1 (NM_059380.5, aa 1–266) was PCR‐amplified and cloned into pB27 as a C‐terminal fusion to LexA (LexA‐CHN‐1). The construct was checked by sequencing the entire insert and used as a bait to screen a random‐primed *C. elegans* mixed‐stage cDNA library constructed into pP6. pB27 and pP6 were derived from the original pBTM116 (Vojtek & Hollenberg, 1995) and pGADGH (Bartel *et al*, 1993) plasmids, respectively. A total of 61 million clones (sixfold the complexity of the library) were screened using a mating approach with YHGX13 (Y187 ade2‐101::loxP‐kanMX‐loxP, matα) and L40Gal4 (mata) yeast strains as previously described (Fromont‐Racine *et al*, 1997). 202 His^+^ colonies were selected on a medium lacking tryptophan, leucine, and histidine and supplemented with 50 mM 3‐aminotriazole to prevent bait autoactivation. The prey fragments of the positive clones were amplified by PCR and sequenced at their 5' and 3' junctions. The resulting sequences were used to identify the corresponding interacting proteins in the GenBank database (NCBI) using a fully automated procedure. A confidence score (PBS, Predicted Biological Score) was attributed to each interaction as previously described (Formstecher *et al*, 2005). The PBS relies on two different levels of analysis. First, a local score considers the redundancy and independence of prey fragments and the distribution of reading frames and stop codons in overlapping fragments. Second, a global score considers the interactions found in all of the screens performed by the Hybrigenics Services using the same library. This global score represents the probability of interaction being nonspecific. The scores were divided into four categories for practical use, from A (highest confidence) to D (lowest confidence). A fifth category (E) flags explicit interactions involving highly connected prey domains previously found several times in screens performed on libraries derived from the same organism.

#### Statistical analysis

For normal distribution with one independent variable experiments, unpaired *t‐*test or one‐way ANOVA analysis was used, while two‐way ANOVA was used for the data with more than one independent variable. For nonlinear data, the correlation between the data sets computed using Pearson's correlation coefficients to determine the extent of statistical relationalship using *P‐*value (Two‐tailed test). Level of significance was represented in the form of *P*‐value (*P < 0.05; **P < 0.01; ***P < 0.001; ****P < 0.0001). All plots, unless stated otherwise, were plotted in the GraphPad Prism 9.

## Author contributions

**Aniruddha Das:** Conceptualization; data curation; formal analysis; validation; investigation; visualization; methodology. **Pankaj Thapa:** Conceptualization; data curation; formal analysis; investigation; visualization; methodology. **Ulises Santiago:** Formal analysis; visualization. **Nilesh Shanmugam:** Formal analysis; investigation. **Katarzyna Banasiak:** Formal analysis; investigation; visualization. **Katarzyna Dąbrowska:** Formal analysis; investigation. **Hendrik Nolte:** Formal analysis; investigation; visualization. **Natalia A Szulc:** Formal analysis; visualization. **Rose M Gathungu:** Investigation. **Dominik Cysewski:** Formal analysis. **Marcus Krüger:** Resources. **Michał Dadlez:** Resources. **Marcin Nowotny:** Resources; supervision. **Carlos J Camacho:** Conceptualization; formal analysis. **Thorsten Hoppe:** Resources. **Wojciech Pokrzywa:** Conceptualization; resources; data curation; formal analysis; supervision; funding acquisition; validation; visualization; project administration.

## Disclosure and competing interests statement

The authors declare that they have no conflict of interest.

## Supporting information



Expanded View Figures PDF

Table EV1

## Data Availability

Computational models, HDX‐MS data, plasmids, antibodies and worms generated by the authors will be distributed upon request to other researchers. The mass spectrometry proteomics data were deposited to the ProteomeXchange Consortium via the PRIDE partner repository with the dataset identifier PXD028023 (Perez‐Riverol *et al*, 2019), and are accessible at http://proteomecentral.proteomexchange.org/cgi/GetDataset?ID=PXD028023
