# Author Correction: A heterotypic assembly mechanism regulates CHIP E3 ligase activity

**DOI:** 10.1038/s44318-024-00042-3

**Published:** 2024-02-22

**Authors:** Aniruddha Das, Pankaj Thapa, Ulises Santiago, Nilesh Shanmugam, Katarzyna Banasiak, Katarzyna Dązbrowska, Hendrik Nolte, Natalia A Szulc, Rose M Gathungu, Dominik Cysewski, Marcus Krüeger, Michał Dadlez, Marcin Nowotny, Carlos J Camacho, Thorsten Hoppe, Wojciech Pokrzywa

**Affiliations:** 1 Laboratory of Protein Metabolism, International Institute of Molecular and Cell Biology in Warsaw, Warsaw, Poland; 2 Department of Computational and Systems Biology, University of Pittsburgh, Pittsburgh, Pennsylvania USA; 3 Institute of Biochemistry and Biophysics, PAS, Warsaw, Poland; 4 Institute for Genetics and Cologne Excellence Cluster on Cellular Stress Responses in Aging-Associated Diseases (CECAD), University of Cologne, Cologne, Germany; 5 Metabolomics Core Facility, EMBL, Heidelberg, Germany; 6 Center for Molecular Medicine (CMMC), Faculty of Medicine and University Hospital of Cologne, 50931 Cologne, Germany; 7 Laboratory of Protein Structure, International Institute of Molecular and Cell Biology in Warsaw, Warsaw, Poland; 8 Present Address: Max-Planck-Institute for Biology of Ageing, Cologne, Germany

## Abstract

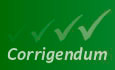

**Correction to:** *EMBO J* (2022) 41:e109566. 10.15252/embj.2021109566 | Published online 28 June 2022

The authors contacted the journal after becoming aware of an error in the terminology of the graphical abstract, the results section of the manuscript and within the Figure 5A figure legend.

**The graphical abstract is corrected**.

**Original:** 
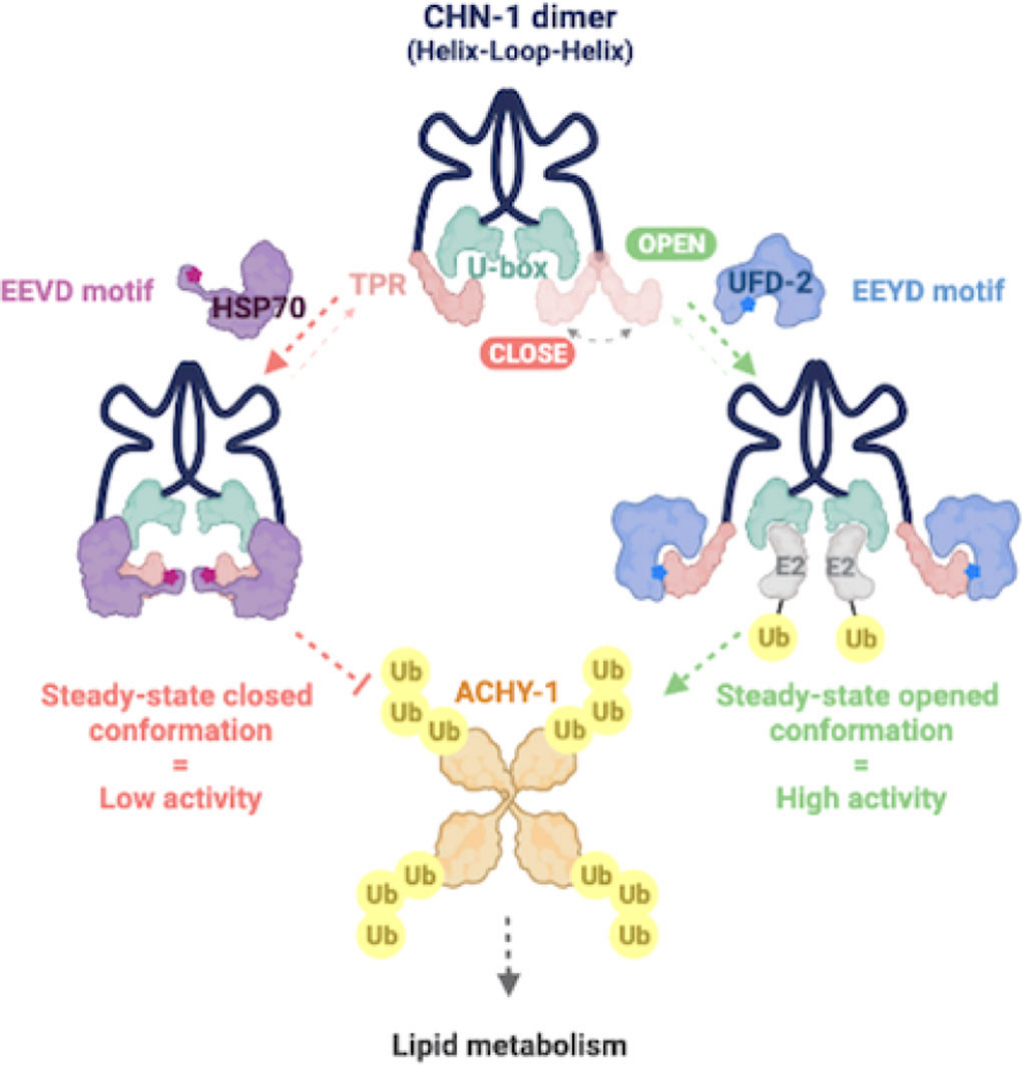


**Corrected:** 
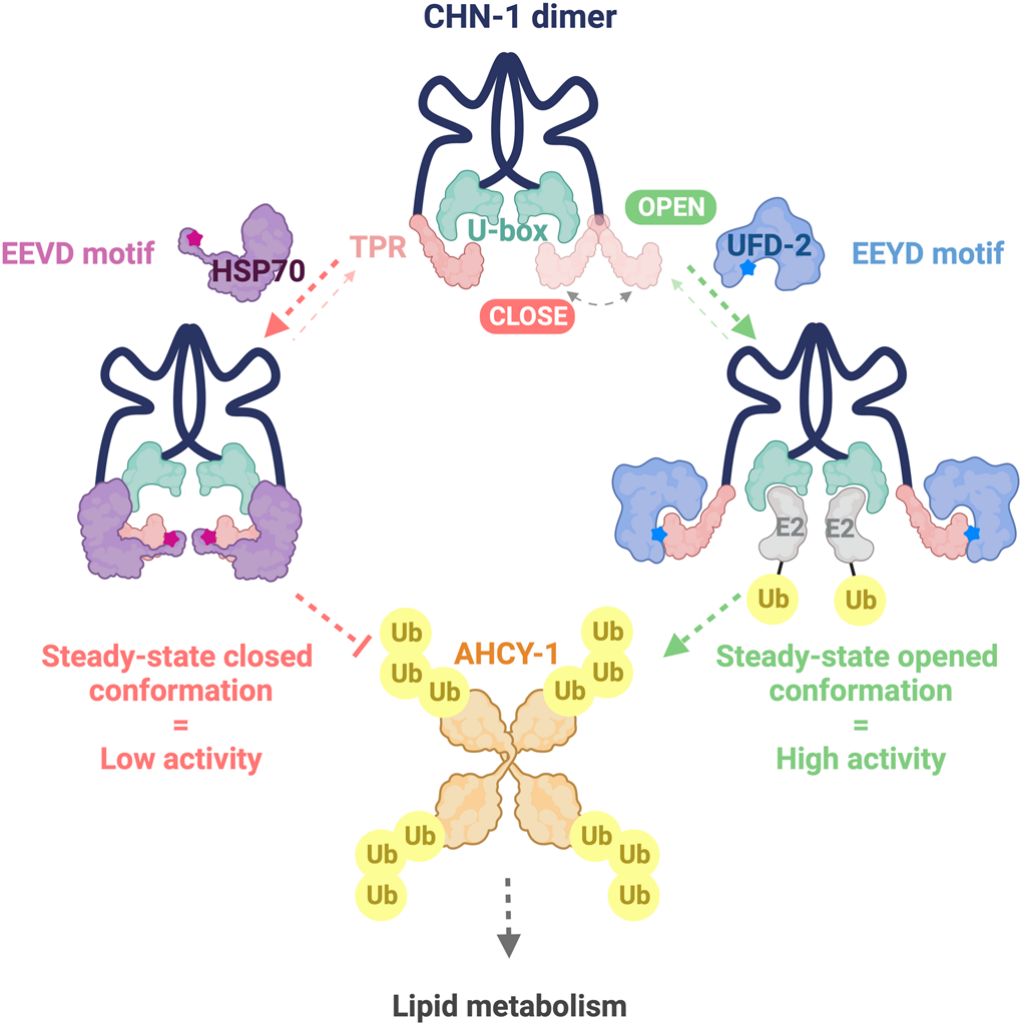


**The Results section is corrected**.

**The Figure 5A figure legend is corrected**.

Page 12, line 12 of the PDF manuscript is corrected from:

“compared with the ACHY-1 status in wild-type animals (Fig 5C). As”

To (Changes in bold):

“compared with the **AHCY-1** status in wild-type animals (Fig 5C). As”

Figure 5A figure legend is corrected from:

“A) Boxplot analysis showing the Z-score of normalized intensities of the 50 LC-MS/MS-identified peptides from ACHY-1 detected in N2 (wild-type), *chn-1(by155)*, *ufd-2(tm1380)*, and *chn-1(by155); ufd-2(tm1380)* mutant worms. The central band of each box is the median value, and the box defines the 25^th^ (lower) and 75^th^ (higher) quantile. The whiskers represent the minimum and maximum values in the data, excluding outliers. A data point is considered an outlier if the distance to the median is greater than 1.5 * inter quantile range distance to the median.”

To (Changes in bold):

“A) Boxplot analysis showing the Z-score of normalized intensities of the 50 LC-MS/MS-identified peptides from **AHCY-1** detected in N2 (wild-type), *chn-1(by155)*, *ufd-2(tm1380)*, and *chn-1(by155); ufd-2(tm1380)* mutant worms. The central band of each box is the median value, and the box defines the 25^th^ (lower) and 75^th^ (higher) quantile. The whiskers represent the minimum and maximum values in the data, excluding outliers. A data point is considered an outlier if the distance to the median is greater than 1.5 * inter quantile range distance to the median.”

These changes do not affect the text or conclusions of the article.

