# Supplementary material for: A heterotypic assembly mechanism regulates CHIP E3 ligase activity
Source: EMBO J. 2022 Jun 28;41(15):e109566. doi: 10.15252/embj.2021109566 (PMC9340540; doi:10.15252/embj.2021109566)
Supplement: Supplementary file 1 — Expanded View Figures PDF [file EMBJ-41-e109566-s001.pdf]

Expanded View Figures

Figure EV1. Interaction with UFD-2 increases the ubiquitylation activity of CHN-1.

A Auto-Ub of recombinant CHN-1 only and in the presence of UFD-2 was carried using UBE2D1, UBE2D2, or UBE2D3 E2. Protein samples were resolved via SDS–PAGE and immunoblotted with anti-CHN-1 antibodies.

B Auto-Ub of recombinant UFD-2 was performed using UBE2D1, UBE2D2, or UBE2D3 E2. Protein samples were resolved via SDS–PAGE and immunoblotted with anti-UFD-2 antibodies.

C Auto-Ub of recombinant CHN-1 only and in the presence of UFD-2 was carried using LET-70 E2. Protein samples were resolved via SDS–PAGE and immunoblotted with anti-CHN-1 antibodies.

D *In vitro* ubiquitylation assay performed in the presence of CHN-1, UFD-2 or both as indicated using UBE2N-Uev1a E2. Protein samples were resolved via SDS–PAGE and immunoblotted with anti-Ub antibodies.

E Auto-Ub of recombinant UFD-2 only and in the presence of CHN-1 was performed as indicated using UBE2N-Uev1a, UBE2D1, or UBE2D3 E2, and Ub^{WT} or Ub^{NoK}. Protein samples were resolved via SDS–PAGE and immunoblotted with anti-UFD-2 antibodies.

F Auto-Ub of recombinant CHN-1^{H218Q} was performed in the presence of UFD-2 or UFD-2^{P951A} as indicated using UBE2D1 E2. Protein samples were resolved via SDS–PAGE and immunoblotted with anti-CHN-1 antibodies. Asterisk (*) on the blot represented the signal that appeared in the presence of UFD-2^{P951A}.

G Time-dependent (0, 30, 60, 120, and 180 min) auto-Ub of CHN-1 only and in the presence of UFD-2 was performed as indicated using UBE2D2 E2. Protein samples were resolved via SDS–PAGE and immunoblotted with anti-CHN-1 antibodies.

H Time-dependent (0, 30, 60, 120, and 180 min) auto-Ub of CHN-1 only and in the presence of UFD-2 was performed as indicated using UBE2D3 E2. Protein samples were resolved via SDS–PAGE and immunoblotted with anti-CHN-1 antibodies.

I Auto-Ub of recombinant CHN-$1^{\Delta 110}$ only and in the presence of UFD-2 was performed as indicated using UBE2D1 E2. Protein samples were resolved via SDS–PAGE and immunoblotted with anti-CHN-1 antibodies.

J Co-immunoprecipitation of ubiquitin-charged GST-UBE2D1 from a mixture of ubiquitin-charged GST-UBE2D1 and CHN-1, ubiquitin-charged GST-UBE2D1 and UFD-2^{P951A}, or the ternary mixture of ubiquitin-charged GST-UBE2D1, CHN-1 and UFD-2^{P951A} using Dynabeads conjugated with anti-GST antibody. Protein samples were resolved via SDS–PAGE and immunoblotted with anti-GST, anti-UFD-2, and anti-CHN-1 antibodies.

K Auto-Ub of recombinant CHN-1 only and in the presence of UFD-2, UFD-2^{P951A}, or UFD-$2^{\Delta Ubox}$ was performed as indicated using UBE2D1 E2. Bands were labeled as Unmodified CHN-1, Multi-mono-Ubiquitylated CHN-1, and Poly-Ubiquitylated CHN-1. Right, quantification of CHN-1 modifications (Unmodified, Multi-mono-ubiquitylated, Poly-ubiquitylated) plotted as percentages. Graph plotted for CHN-1 alone (black), CHN-1 + UFD-2 (cyan), CHN-1 + UFD-2^{P951A} (yellow), or CHN-1 + UFD-$2^{\Delta Ubox}$ (magenta). Plotted data are the mean of three technical replicates. Error bars represent the SEM; statistical significance was determined using a two-way ANOVA test (****$P < 0.0001$).

Data information: Representative immunoblots for at least three independent experiments are shown in the panels.

© 2022 The Authors

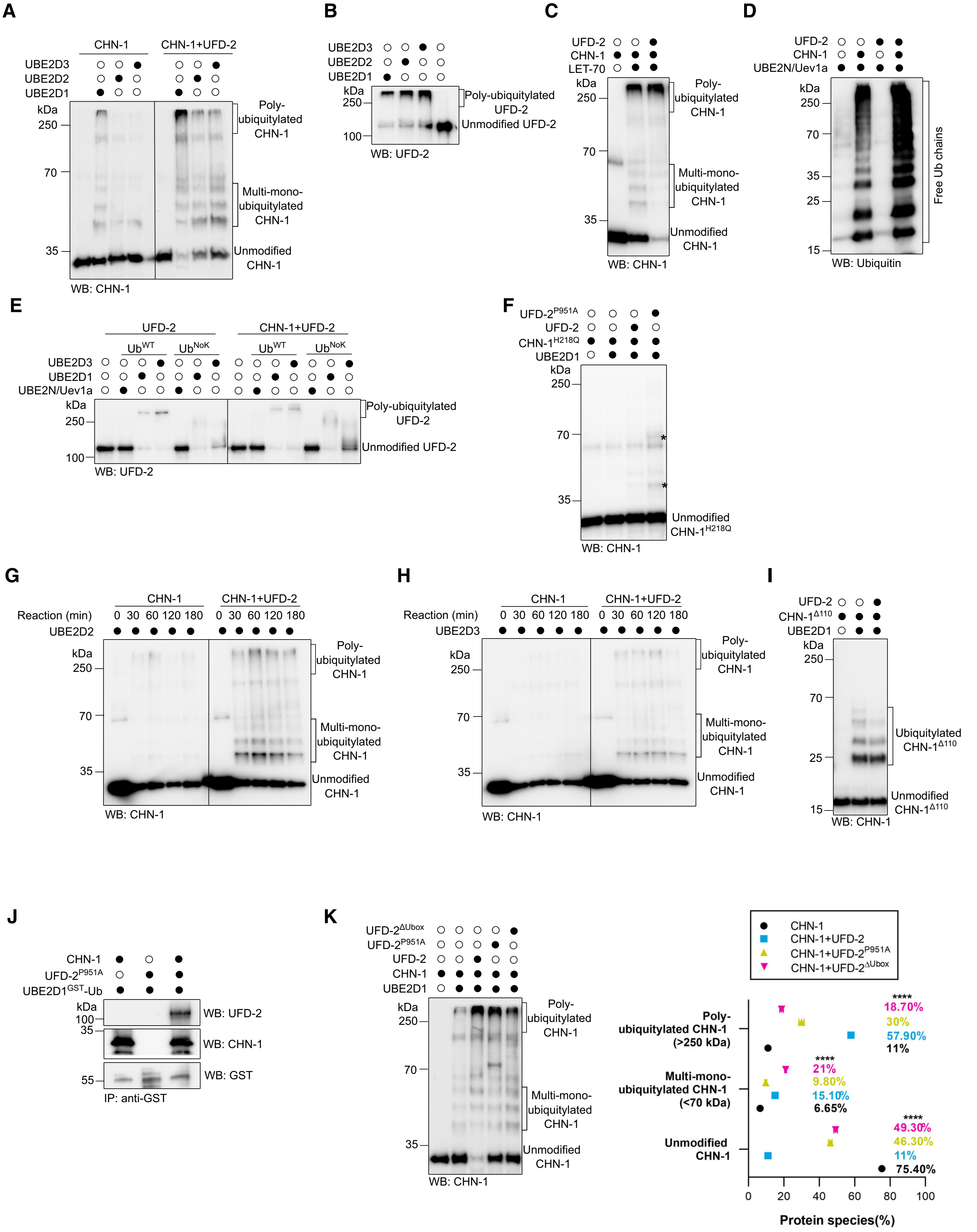

Figure EV1.

 © 2022 The Authors

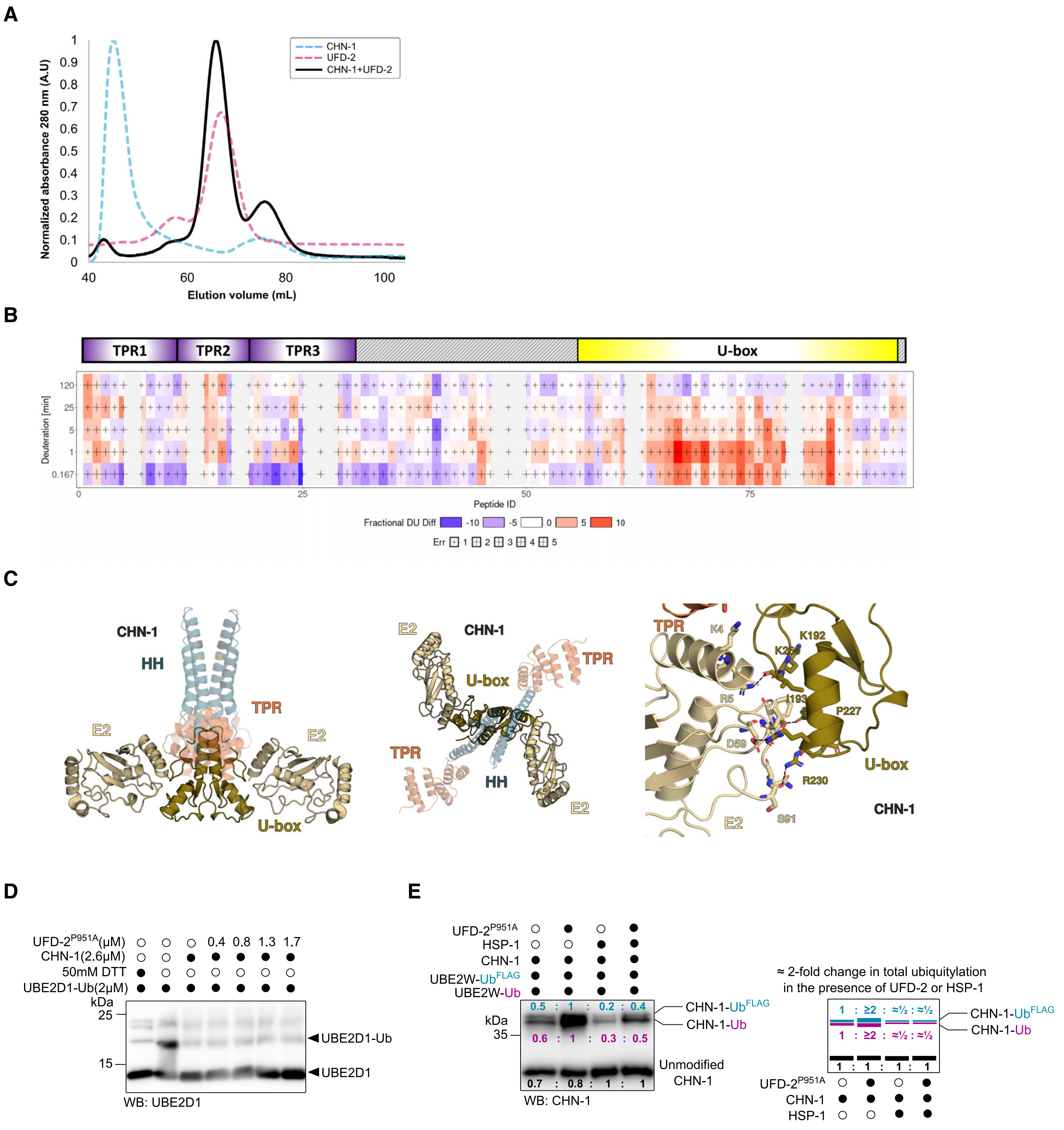
A
Normalized absorbance 280 nm (A.U)
Elution volume (mL)
CHN-1
UFD-2
CHN-1+UFD-2
B
TPR1
TPR2
TPR3
U-box
Deuteration [min]
Peptide ID
Fractional DU Diff
Err
C
CHN-1
HH
TPR
E2
U-box
K4
K192
R5
I193
P227
D59
R230
S91
D
UFD-2P951A(μM)
CHN-1(2.6μM)
50mM DTT
UBE2D1-Ub(2μM)
kDa
UBE2D1-Ub
UBE2D1
WB: UBE2D1
E
UFD-2P951A
HSP-1
UBE2W-UbFLAG
UBE2W-Ub
CHN-1-UbFLAG
CHN-1-Ub
Unmodified CHN-1
WB: CHN-1
≈ 2-fold change in total ubiquitylation in the presence of UFD-2 or HSP-1

Figure EV2.

Figure EV2. UFD-2, unlike HSP-1, potentiates E2 accessibility of CHN-1.

A Size-exclusion chromatography (SEC) profiles of the recombinant proteins CHN-1 (cyan), UFD-2 (magenta), and CHN-1 + UFD-2 mixture (black) resolved in the S200 Superdex column.

B Chiclet plot showing the differences in deuterium uptake by CHN-1 peptides due to the presence of UFD-2 across the five time points. The X-axis spans the peptide length of CHN-1 and the time points are plotted on the Y-axis (total of 99 peptides with 84.2% sequence coverage and 4.55 redundancy). Above the chiclet plot is the domain organization of CHN-1, indicating TPR and U-box domains.

C Model of the CHN-1 U-box dimer with two E2 enzymes. UbcH5 E2 (gold) (PDB ID: 2OXQ) was aligned to the co-crystal structure of CHIP (*Danio rerio*). The two structures aligned with low RMSD = 0.376. Marked are conserved residues that stabilize the critical interaction between the U-box domain and E2.

D Discharging assay of Ub-charged UBE2D1 was carried with increasing molar concentrations of recombinant UFD-2^{P951A} as indicated. The reaction was stopped after 30 min via the addition of Laemmli sample buffer. Protein samples were resolved via SDS–PAGE and immunoblotted with anti-UBE2D1antibodies.

E CHN-1 auto-Ub was performed as indicated in the presence of Ube2W-Ub or Ube2W-Ub^{FLAG} with and without a complexing equimolar concentration of recombinant CHN-1 and UFD-2^{P951A} and in the absence or presence of HSP-1. Protein samples were resolved via SDS–PAGE and immunoblotted with anti-CHN-1 antibodies. For each sample, the quantified relative signal after probing the blot using anti-CHN-1 antibodies is represented as a ratio above the respective signal. Right, schematic of the CHN-1-Ub^{FLAG} (cyan), CHN-1-Ub (magenta), and unmodified CHN-1 signal (black) presented as ratio and the signal fold change among CHN-1, CHN-1 + UFD-2^{P951A}, CHN-1 + HSP-1, and CHN-1 + UFD-2^{P951A} + HSP-1.

Data information: Representative immunoblots for at least three independent experiments are shown in the panels. Source data for HDX-MS measurements are available in the Table EV1.

Figure EV3. CHN-1 activity switch is induced by the interaction of its TPR domain with the HSP-1 EEVD or UFD-2 EEYD motif.

A ELISA-based titration assay to determine the dissociation constants (K_D) between DAF-21, HSP-1, UFD-2, and CHN-1. Y-axis: CHN-1 concentration (μM). X-axis: absorbance (OD) value at 450 nm as a function of the converted substrate (Alkaline Phosphatase Yellow). Below, a table showing the K_D value (nM) of the corresponding protein with recombinant CHN-1. Plotted data are the mean of three technical replicates. Error bars represent the SEM.

B ELISA-based titration assay performed using recombinant CHN-1, UFD-2, and DAF-21 with the results plotted as the DAF-21 concentration (μM) vs. absorbance (OD) value at 450 nm as a function of the converted substrate (Alkaline Phosphatase Yellow). Plotted data are the mean of three technical replicates. Error bars represent the SEM.

C ELISA-based titration assay performed using recombinant CHN-1, UFD-2, and HSP-1 with the results plotted as the HSP-1 concentration (μM) vs. absorbance (OD) value at 450 nm as a function of the converted substrate (Alkaline Phosphatase Yellow). Plotted data are the mean of three technical replicates. Error bars represent the SEM.

D *In vitro* ubiquitylation assay was performed as indicated using Ub-charged UBE2D1 in the presence of CHN-1, UFD-2^{P951A} or ternary mixture of recombinant CHN-1, UFD-2^{P951A} and HSP-1. The reaction was stopped after the indicated time via the addition of Laemmli sample buffer. Protein samples were resolved via SDS–PAGE and immunoblotted with anti-Ubiquitin antibodies.

E Multiple sequence alignment (MSA) of UFD-2 from different species. Orthologous sequences from selected species were obtained from the eggNOG5 database (from Orthologous Group ID ENOG5038DSP) (Huerta-Cepas *et al*, 2019) and aligned using the T-Coffee web server with default parameters (Notredame *et al*, 2000; Di Tommaso *et al*, 2011). Vertebrates possess two UFD-2 orthologs, which have been independently annotated. The MSA was visualized in the Jalview Desktop software (Waterhouse *et al*, 2009) with residues colored according to their physicochemical properties; conserved tyrosine (Y) residues and the EEYD motif in *C. elegans* are highlighted in white frames.

F *In vitro* ubiquitylation assay was performed as indicated using an increasing concentration of Ub-charged UBE2D1 (1.6, 3.3, 5, 6.6 μM) in the presence of CHN-1 or CHN-1 and HSP-1^{EEYD}. The reaction was stopped after 30 min via the addition of Laemmli sample buffer. Protein samples were resolved via SDS–PAGE and immunoblotted with anti-Ubiquitin antibodies.

G Auto-Ub of recombinant CHN-$1^{\Delta 87}$ (cyan) or CHN-$1^{\Delta 95}$ (magenta) truncation mutants as indicated using UBE2D1 E2 in the presence of UFD-2, DAF-21, DAF-$21^{\Delta EEVD}$, HSP-1 or HSP-$1^{\Delta EEVD}$. Samples were analyzed via SDS–PAGE and immunoblotted with anti-CHN-1 antibodies. Cyan asterisk (*) on the blot represented the auto-ub of CHN-$1^{\Delta 87}$ and magenta asterisk (*) on the blot represented the auto-ub of CHN-$1^{\Delta 95}$.

H Model of the CHN-1 TPR domain docked with the UFD-2 EEYD peptide. Residues 1–86 are colored in orange and residues 87–95 of CHN-1, which sequester the EEYD motif away from the CHN-1 R230 residue, are colored in magenta.

I A co-crystal structure of the murine CHIP TPR domain interacting with the HSP90 EEVD peptide (PDB ID 2C2L) reveals that CHIP R273 (conserved in CHN-1 as R230) is sufficiently close in proximity to interact with HSP90 D501.

Data information: Representative immunoblots for at least three independent experiments are shown in the panels.

 © 2022 The Authors

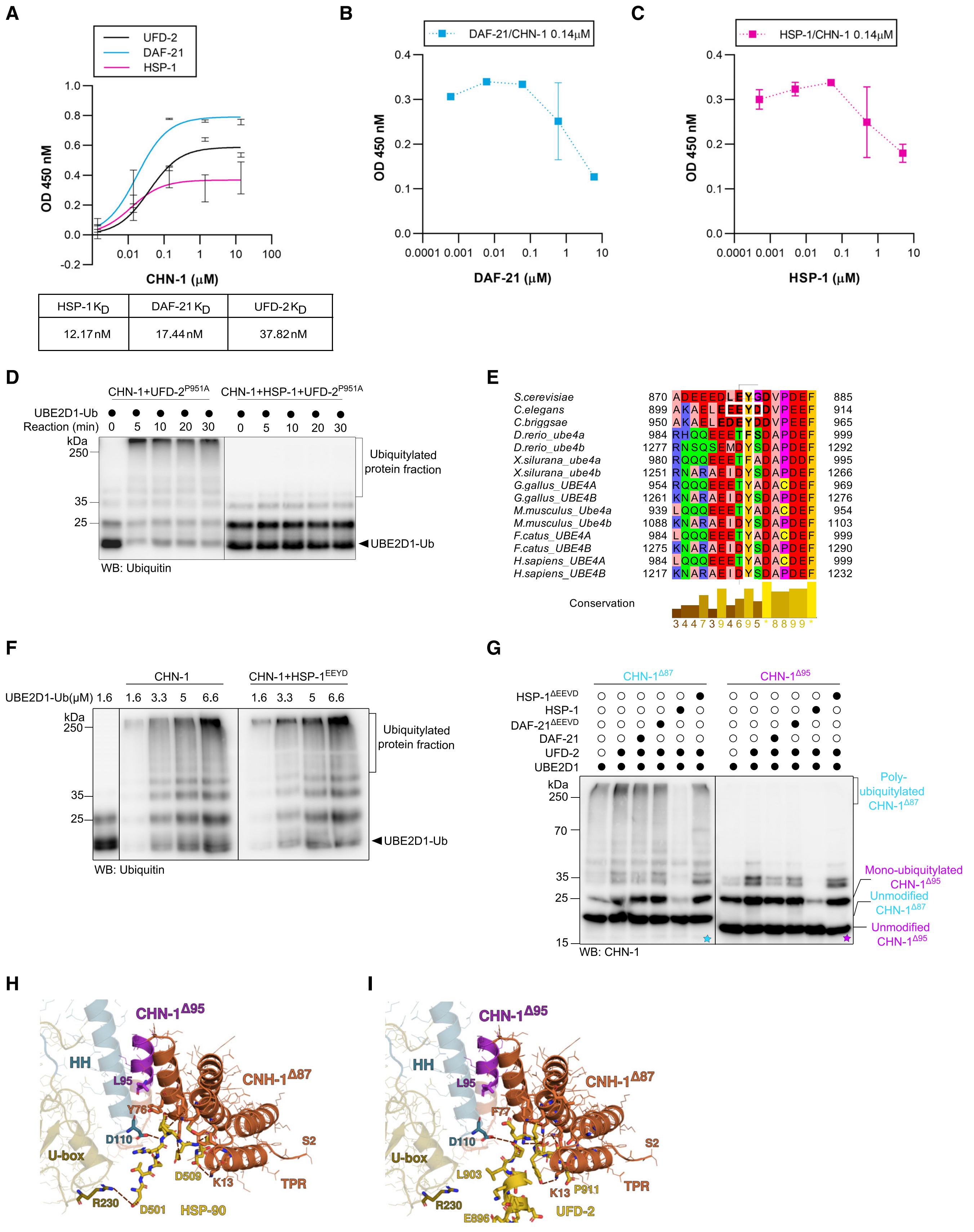

HSP-1 K_D	DAF-21 K_D	UFD-2 K_D
12.17 nM	17.44 nM	37.82 nM

Figure EV3.

Figure EV4. Poly-ubiquitylation of AHCY-1 is mediated by the CHN-1-UFD-2 complex.

A PCA showing the first and second principal components of the significantly altered proteins (ANOVA FDR < 0.05) performed in the Perseus software (Tyanova *et al*, 2016). The percentage of explained variance is represented on the axis labels.

B Schematic representation of the number of identified proteins in a single-shot analysis of LC-MS/MS gradients in five biological replicates that led to the identification of proteins with significant abundance changes in *chn-1(by155)*, *ufd-2(tm1380)*, and *chn-1(by155); ufd-2(tm1380)* worms (twofold enrichment in all mutants versus wild-type N2 animals).

C Hierarchical clustering of the Z-scores of proteins whose levels increased in *chn-1(by155)*, *ufd-2(tm1380)*, and *chn-1(by155); ufd-2(tm1380)* mutant worms (twofold enrichment in all mutants versus wild-type N2 animals from the LC-MS/MS experiment).

D Gene ontology biological process terms found to be associated with *C. elegans* genes upregulated (minimum twofold enrichment versus N2 (control), with FDR < 0.05 for ANOVA or pairwise *t*-test) in all mutants; all proteins detected in the LC-MS/MS analysis comprised a reference set. Overrepresentation analysis was performed using the WebGestalt web server with default parameters (Liao *et al*, 2019). FDR was controlled to 0.25 using the Benjamini-Hochberg method for multiple testing.

E Yeast two-hybrid prey fragment analysis. Schematic representations of the AHCY-1 fragments interacting with CHN-1 identified in the yeast two-hybrid screen (Hybrigenics). The coding sequence for CHN-1 was used as bait to screen a random-primed *C. elegans* mixed-stage cDNA library. The selected interaction domain (SID) is shown in yellow.

F Co-immunoprecipitation of AHCY-1 from young adult worms expressing CHN-1::FLAG using beads conjugated with anti-FLAG antibody. Protein samples were resolved via SDS–PAGE and immunoblotted with anti-AHCY-1 and anti-FLAG antibodies (the red boxes mark the protein band).

G CHN-1 auto-Ub was performed as indicated. Protein samples were resolved via SDS–PAGE and immunoblotted with anti-CHN-1 antibodies.

H Ubiquitylation of recombinant AHCY-1 was performed as indicated using recombinant UFD-2 and UBE2D1 E2. Protein samples were resolved via SDS–PAGE and immunoblotted with anti-AHCY-1 antibodies.

I Ubiquitylation of recombinant AHCY-1 was performed as indicated using recombinant CHN-1, UFD-2, DAF-21, or HSP-1 in the presence of UBE2D1 E2. Protein samples were resolved via SDS–PAGE and immunoblotted with anti-AHCY-1 antibodies.

J Quantitative PCR analyses of *ahcy-1* transcript levels in young adult N2 (wild-type; black), *chn-1(by155)* (magenta) and *ufd-2(tm1380)* (cyan) worms. Plotted data are the mean of three biological replicates. Error bars represent SEM; statistical significance was determined using a one-way ANOVA test.

K Protein level of endogenous AHCY-1 in N2 (wild-type), *chn-1(by155)*, and *ufd-2(tm1380)* young adult worms. After centrifugation, the supernatant obtained from the worm lysate and the resulting pellet were dissolved in 5% SDS and boiled for 5 min. Protein samples were resolved via SDS–PAGE and immunoblotted with anti-AHCY-1 antibodies.

Data information: Representative immunoblots for at least three independent experiments are shown in the panels.

© 2022 The Authors

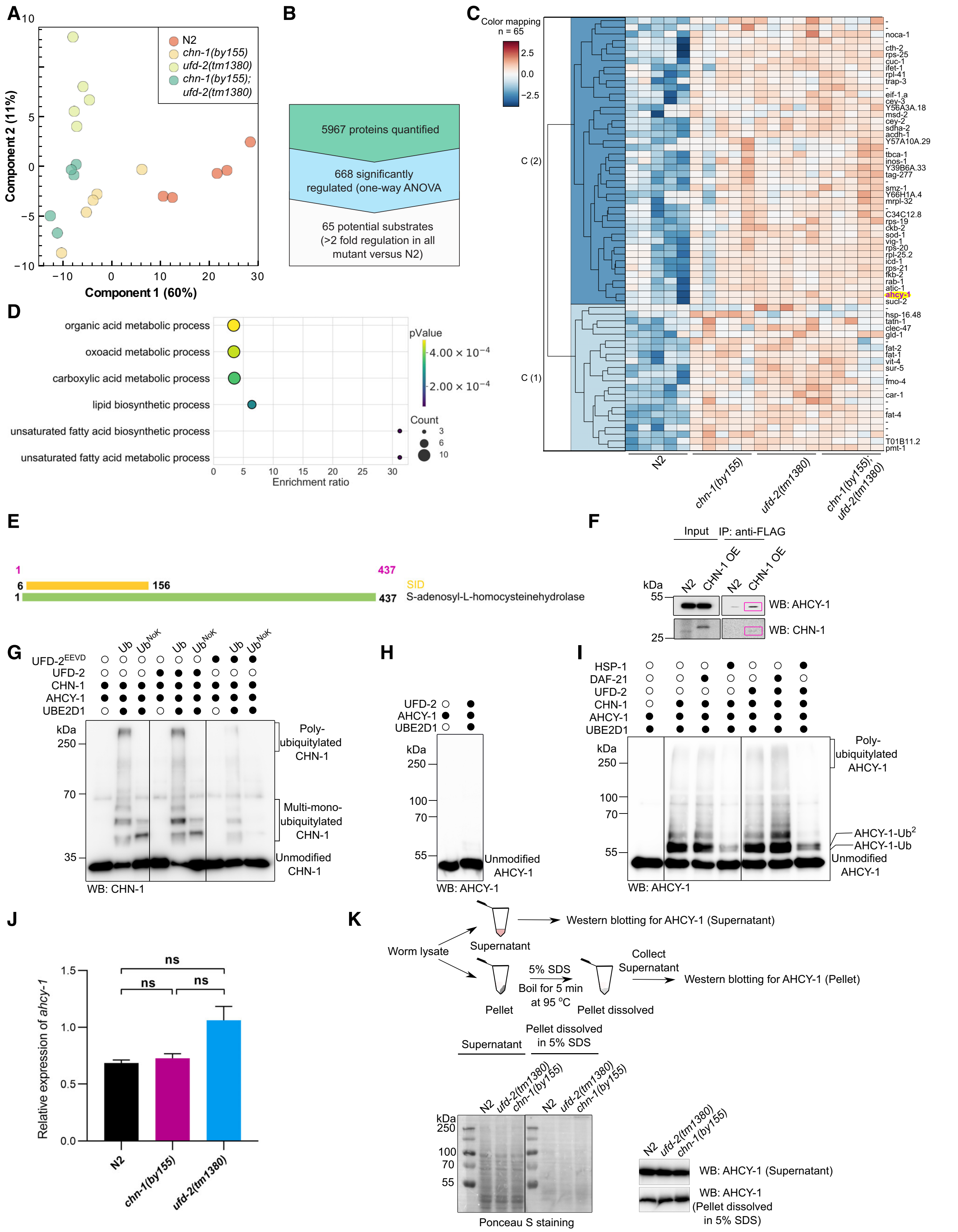
A
N2
chn-1(by155)
ufd-2(tm1380)
chn-1(by155); ufd-2(tm1380)
Component 2 (11%)
Component 1 (60%)
B
5967 proteins quantified
668 significantly regulated (one-way ANOVA
65 potential substrates (>2 fold regulation in all mutant versus N2)
C
Color mapping n = 65
C (2)
C (1)
N2
chn-1(by155)
ufd-2(tm1380)
chn-1(by155); ufd-2(tm1380)
D
organic acid metabolic process
oxoacid metabolic process
carboxylic acid metabolic process
lipid biosynthetic process
unsaturated fatty acid biosynthetic process
unsaturated fatty acid metabolic process
Enrichment ratio
pValue
Count
E
SID
S-adenosyl-L-homocysteinehydrolase
F
Input
IP: anti-FLAG
N2
CHN-1 OE
WB: AHCY-1
WB: CHN-1
G
UFD-2EEVD
UFD-2
CHN-1
AHCY-1
UBE2D1
Poly-ubiquitylated CHN-1
Multi-mono-ubiquitylated CHN-1
Unmodified CHN-1
WB: CHN-1
H
UFD-2
AHCY-1
UBE2D1
Unmodified AHCY-1
WB: AHCY-1
I
HSP-1
DAF-21
UFD-2
CHN-1
AHCY-1
UBE2D1
Poly-ubiquitylated AHCY-1
AHCY-1-Ub2
AHCY-1-Ub
Unmodified AHCY-1
WB: AHCY-1
J
Relative expression of ahcy-1
ns
N2
chn-1(by155)
ufd-2(tm1380)
K
Worm lysate
Supernatant
Western blotting for AHCY-1 (Supernatant)
Pellet
5% SDS
Boil for 5 min at 95 °C
Collect Supernatant
Pellet dissolved
Western blotting for AHCY-1 (Pellet)
Pellet dissolved in 5% SDS
Ponceau S staining
WB: AHCY-1 (Supernatant)
WB: AHCY-1 (Pellet dissolved in 5% SDS)

Figure EV4.

Funding Information – Author Query

AUTHOR: Please check that the funding details given below match the details given in the Acknowledgement and provide any missing funder details. In compliance with certain funding agencies (e.g., US Department of Energy), please add the FundRef DOI(s), which can be found, for example, at: http://www.wiley-vch.de/fundref/

1) Please check that the funding details given below list all funding agencies and grant numbers given in the Acknowledgement and provide any missing funders and funder DOIs where required by you funding agency with the proof corrections.
2) Please check that the funder agency name and DOI is provided according to the official FundRef listing.

**FundRef funding agency name**	**FundRef Doi**	**Grant number**
Fundacja na rzecz Nauki Polskiej (FNP)		POIR.04.04.00-00-5EAB/18-00
National Science Center, Poland (NCN)	10.13039/501100004281	2016/23/B/NZ3/00753, 2021/41/N/NZ1/03086
European Research Council (ERC)	10.13039/501100000781	CoG-616499
Deutsche Forschungsgemeinschaft (DFG)	10.13039/501100001659	390661388, 2030-390661388
HHS ¦ National Institutes of Health (NIH)	10.13039/100000002	R01-GM097082
