# Supplementary material for: A heterotypic assembly mechanism regulates CHIP E3 ligase activity
Source: EMBO J. 2022 Jun 28;41(15):e109566. doi: 10.15252/embj.2021109566 (PMC9340540; doi:10.15252/embj.2021109566)
Supplement: Supplementary file 3 [file EMBJ-41-e109566-s003.pdf]

THE EMBO JOURNAL

A Heterotypic Assembly Mechanism Regulates CHIP E3 Ligase Activity

Aniruddha Das, Pankaj Thapa, Ulises Santiago, Nilesh Shanmugam, Katarzyna Banasiak, Katarzyna Dabrowska, Hendrik Nolte, Natalia Szulc, Rose Gathungu, Dominik Cysewski, Marcus Krüger, Michał Dadlez, Marcin Nowotny, Carlos Camacho, Thorsten Hoppe, and Wojciech Pokrzywa
DOI: 10.15252/embj.2021109566

Corresponding author(s): Wojciech Pokrzywa (wpokrzywa@iimcb.gov.pl)

Review Timeline:

Submission Date:	26th Aug 21
Pre-decision consultation:	6th Oct 21
Pre-decision response:	11th Oct 21
Editorial Decision:	21st Oct 21
Revision Received:	4th May 22
Editorial Decision:	23rd May 22
Revision Received:	25th May 22
Accepted:	27th May 22

Editor: Hartmut Vodermaier

Transaction Report:

(Note: With the exception of the correction of typographical or spelling errors that could be a source of ambiguity, letters and reports are not edited. Depending on transfer agreements, referee reports obtained elsewhere may or may not be included in this compilation. Referee reports are anonymous unless the Referee chooses to sign their reports.)

Thank you again for your patience with the delayed review of your manuscript on distinct CHIP assemblies. Since one of the three experts who had agreed to review the study has, despite multiple reminders, still not returned a report, I am now forwarding you the comments of the other two referees. As you will see, both of them acknowledge the potential interest of your findings, but at the same time point out a number of substantive concerns that would make commitment to publication appear premature at this stage. Given that the majority of the raised issues are of technical or presentational nature, I realize that the paper might still become a more compelling candidate after extensive revisions; but since it is not always clear how these points might be best addressed, and whether this may in fact lead to the required substantiation, I would in this case appreciate hearing from you how you would envision responding to the referees' points should you be given the opportunity to revise this work for The EMBO Journal. Therefore, please carefully consider the attached reports together with your co-authors, and send back a tentative point-by-point response outlining how the referees' comments might be addressed/clarified, based on which we may further discuss via email or Zoom call before taking a definitive decision on this manuscript. It would be great if you could get back to me with such a response over the course of the next week. Should we in the meantime still receive the outstanding comments of referee 3, I would of course forward them to you too.

REFEREE REPORTS

Referee #1 (Report for Author)

The manuscript is definitely of interest to the field of cell signalling and those interested in the mechanisms of E3-ligase regulation as well as more broadly, as it explores a novel mechanism for the stimulation of CHN-1 activity by UFD-2. Though I support publication I am not convinced that currently the data, or the way it is presented and discussed, is of sufficient quality to merit publication in EMBO J. There is quite a lot of work required to improve the manuscript.

The writing of the manuscript feels rushed with the methodology and data not being fully explained - there are several instances where data is not referred to or where the methodology is poorly linked to the data. Thus, though there is a comprehensive methods section, how the methods are applied and the relevance of the data obtained are often not fully developed in the results section, making it a difficult to follow. The strength of the study is the range of approaches used and the evident attempt to link mechanistic insight to new physiologically relevant substrates. However, the breadth of the study is also a weakness as the physiological data is 'shoehorned' into a single figure and is not fully explored. I would prefer to see a structure orientated paper where the full range of E3 assays (from Fig 1) where carried out on the novel substrate and where the physiological study was perhaps left to a follow-up study where it could be more fully developed. The HD-MX study is pivotal to the manuscript yet it has not been fully utilised, there are technical issues and the data is presentation poorly.

Major points:

• Fig 1: Many of the quantitative statements in paragraph 1 of the results section are not supported by the methodology used or the lack of quantitation (e.g. densitometry), making

use of the term significant (line 109) inappropriate. The ubiquitination assays are performed over a 90 min incubation period and some of the reactions appear to have gone to completion suggesting that they are not looking at stead state kinetics. In order to ensure that CHN-1 is rate limiting whilst also avoiding substrate depletion, which is clearly seen for example in Fig 1 lane 6, the length of the assay should have been optimise. In fact, it would have been appropriate to show a time course between 0-60 min. It is likely that optimisation would actually have made the results more definitive as the data presented over-estimate the relative activity of the CHN-1:UBE2D2(3) combination, compared to UBE2D1. Hence the statement on the ability of UBE2D2(3) to catalyse CHN-1 Ub with UFD-1 is misleading. The time-course for Ub (60-90min; Fig 1B) is again a strange choice as the reaction has clearly gone to completion and the substrate (in this case CHN-1) has been depleted at the first time point (60 min) under some conditions. The time course should cover shorter time point to make the data more quantitative allowing estimation of relative differences in autoubiquitination activity. It would also have been of interest to show the effect of titrating UFD-1 from a low stoichiometry to a 1:1 ration this would also support the model proposed in the later sections.

• HD-MX: The study relies heavily on the HD-MX data to develop a model.
The experiment was apparently performed in technical triplicates, but for only n=1. However, many journals are now holding authors to n=3, the reproducibility can be questioned with n=1 as HDX experiments are extremely sensitive to environmental conditions, like temperature, buffer components, ionic strength, exchange and quenching pH, digestion conditions. (The authors may wish to refer to: Masson et al. Recommendations for performing, interpreting and reporting HDX-MS experiments. Nat Methods 16; 595-60). This might be relevant as it appears some of the steps were processed manually. For example, the authors state that 'after quenching, the samples were frozen in liquid nitrogen and kept at -80{degree sign}C until mass spectrometry'. How long was the material stored at -80{degree sign}C, and does storage and rethawing impact on peptides recovered and/or back exchange, etc?
Representation of the HD-data in EV2 and Fig 2 is poor and doesn't make it easy to assess the quality of the data or the authors interpretation. There seem to be peptides that are not deuterated in CHN-1 in the presence or absence of UFD-2. What was the % peptide cover for the undeuterated protein? If there is a discrepancy in undeuterated to deuterated peptide detection it could indicate that the undetected/undeuterated peptides provide insights into the mechanism. Please provide information on the specific peptides not recovered, if there are any and how they map onto the cartoon in figure 2. This may reveal possible additional allosteric/or direct binding effects.
When the authors form the 1:1 complex, the sample is then diluted with deuterated buffer. What does this dilution do to the stability of the complex? For example, are you looking at re-binding after complex dissociation in response to dilution? In other words, early (10 sec or 60 sec) data may reflect changes as a consequence of dilution (i.e. the complex might not be at steady state in the first seconds of dilution). The point is, that HDX is a very dynamic method and what takes place 10 or 60 sec after a 10-20 fold dilution of a preformed complex is not clear. A longer HDX might reveal equilibrium effects. As the authors use a pre-formed complex they should show if the HD-MX is different when the binding partners are combined immediately prior to deuteration. Also, as peptide regulators are identified I am suppressed that the HD-MX analysis was not repeated with the activating peptide. The authors need to discuss the limitations of the study as well as possible protein dynamics effects resulting from the dilution/deuteration/time course method.

• The HD-MX data is over interpreted and poorly explained in the sections between

lines 160-173. I would suggest presenting the figures in a way that helps the reader to get the most out of the data, for example using a more detailed colour code to map % D2O onto the structure. I also think the HD-MX study could be extended (as suggested above) and strengthened using additional experimental approaches to support the authors claims.

• Fig 2B: Why is the format of the data suddenly changes? In order to interpret the data the ubiquitination also needs to be shown.

• Fig 2C: The data and explanation of this figure is inadequate. The experiment is designed to detect Ub, when it would make much more sense to detect the E2 component, so that the ratio of charged and uncharged E2 could be determined. The UBE2D1Ub band assignment has not been validated by including a UBE2D1Ub plus reducing agent control. E2 discharge assays are notoriously sensitive to pH, temperature, etc and are known to discharge over time in the absence of E3, a buffer only control over the course of the experiment is therefore essential. It would have added to the figure to show a titration of CHN-1 and UFD-2 with fixed E2. There are similar issues with EV2B.

• I may have missed it but I can't find any reference to technical replicates in the MS methodology. I would expect to see technical triplicates for each biological replicate as standard.

• Fig 4D: The AHCY-1 portion of the immunoblot is over-exposed (especially when compared to the tubulin control); it should be redone and quantified using densitometry to determine the levels relative to tubulin. However, by eye it seems fairly evident that AHCY1 levels are decreased in the chn-1 KO relative to the WT and flag controls (in relation to tubulin). This is not unprecedented as CHIP has been identified as both a positive and negative regulator of the steady state levels of some substrates dependent on cell stress conditions. Despite not seeing a 'stabilisation' (i.e. increase in steady state levels) of the AHCY-1 protein (by immunoblot) with loss of CHN-1 function the authors still interpret the lipidomics in terms of AHCY-1 stability - presumably on the basis of the proteomics. However, caution is required during the interpretation of LC-MS/MS data as it reflects differences in the abundance of ionized peptides and not necessarily differential protein steady state levels. The abundance of ionized peptides can be influenced by a combination of factors, including post-translational modifications, inter-molecular interactions and compatibility with MS detection (ionization and fragmentation potentials).

• Discussion: The discussion is a bit limited and is treated more as a summary of the authors own findings than a forum for a more critical analysis of the data in respect to the literature. For example, human HSP70, as highlighted by the authors, inhibits CHIP on some folded substrates, however it has also been shown to stimulate the modification of some others, most convincingly the co-chaperone BAG-1. How can this be explained in light of their model? Another area that is not discussed, is the potential role here of CHN-1/CHIP-mediated ubiquitination that doesn't signal degradation - this is very pertinent as the current study does not show up regulation of AHCY-1 steady state levels in KO worms. Finally, as pointed out by the author a large portion of the literature around CHN-1/CHIP is focused on proteostasis and its role as a co-chaperone for HSP70/HSP-1. The manuscript is crying out for a critical evaluation of how the model presented here would inform the current view of CHN-1/CHIP function.

Other comments:

• The text states that assays with worms plus MG132 and NEM are shown but I do not see the data?
• Fig EV1C: It appears that the exposure time in figure EV1C is a lot shorter than in other figures (compare for example the unmodified band in Fig EV1F) to which it is being compared. Never the less it is possible to discern bands that are present in the plus UFD-2 lane compared to the minus UFD-2 lane. There should be a positive control present on the immunoblot that allows comparison with other gels and a comment should be made about these bands.
• There is no plus UFD-2 minus CHN-1 control in figure EV1B.
• The authors should comment on the fact that addition of UFD-2 to ΔTPR-CHN-1 actually decreases the amount of Ub detected.
• S3F (line 242 and 245) should be EV3F, there are several other places where EV is replaced with S.
• Fig EV4G: A minus E3 control needed.
• Fig 4C: K48 and K63 are used in the assay but the data do not appear to be referred to in the text and should therefore be removed from the figure.

Referee #2 (Report for Author)

In this manuscript, Das et al reported that UFD-2 promotes the dimerization of CHN-1 U-box domains to stimulates the autoubiquitylation of CHN-1. They proposed that HSP-1 stabilizes the autoinhibitory state of CHN-1, limiting interactions between E2s and UFD-2. The authors further showed that the interaction with UFD-2 enables CHN-1 to regulate lipid metabolism by ubiquitylation of AHCY-1. While this is an interesting observation, several issues still need to be addressed before its publication.
Major concerns:
1) It is very interesting that UFD-2 stabilizes CHN-1 U-box dimer, whereas the authors only show the HDX-MS results. Size exclusion chromatography profiles of CHN-1 alone, CHN-1 and UFD-2 (or UFD-2 PEPTIDE2) may be helpful. In addition, they have mapped the binding regions in UFD-2 EEYD, whether the mutation in this region (UFD-2 EEYD mutations) could abolish the function of UFD-2 in CHN-1 U-box dimer stabilization and ubiquitylation activity?

2) The authors claim that UFD-2 stabilizes the U-box dimer with two E2 sites, whereas their results could not sufficiently support this major point, more evidences are definitely needed to support this conclusion:

First, they showed UBE2D1GST-Ub (while they have not described how to produce ubiquitin-charged GST-UBE2D1 in the methods or legends) could directly bind to CHN-1, however, UFD-2(P951A) could not enhance the amount of CHN-1 in the immunoprecipitate in Figure EV1E. They should further confirm CHN-1/UFD-2 could bind with two E2.

Second, authors showed UFD-2 (P951A) could dramatically enhance the autoubiquitylation of CHN-1 by using equal amount of UBE2D1 in Figure 2B, whereas the effective control groups are loss. The half and 2-fold of UBE2D1 in Figure 2B lane 6 should be used in the CHN-1/UFD-2 ubiquitination assay.

Third, the E2-discharging assay is problematic. The discharging reaction samples should also be treated with reducing agent and detected with UBE2D1 blotting to further confirm the UBE2D1-Ub bands. In addition, the auto ubiquitylated CHN-1 and Di-Ub should be observed

to reflect the activity of CHN-1.

As there are some reported U-box:E2 structure (PMID: 20696396, 18485199, 20146531, 16307917), the authors could dock them with the stabilized CHN-1 dimer to further support their statements.

3) Given that HSP-1 could diminish the activity of CHN-1/UFD-2 and HSP-1 shows a higher binding affinity with CHN-1 than UFD-2, whether HSP-1 could compete with UFD-2 for binding CHN-1?

4) AHCY-1 is a newly identified substrate for CHN-1/UFD-2, whereas the protein level of AHCY-1 is not consistent in MS data and Western blot in Figure 4A and 4D. As the authors claim that assembly with UFD-2 enables CHIP/CHN-1 to regulate lipid metabolism by ubiquitylation of AHCY-1, the UFD-2 EEYD mutations should be provide as negative control in both AHCY-1 ubiquitination assay and the lipid examination.

Minor comments:
1. Figure 1A, why the authors do not used the E2 of CHN-1 in C. elegans (UBC-2/Let-70, PMID: 15294159)?
2. Figure 1B, time-dependent auto-Ub experiment sounds not work, the authors should shorten time.
3. Figure EV1C, CHN-1 signal could be observed in lane without CHN-1?
4. Figure 2B, C, as the authors state that immunoblots representative of n = 3 experiments are shown, statistical analysis could be performed. Same things in Figure 3B, 3C and 4C.
5. It is very interesting that a single point mutation in HSP-1 (EEVD to EEYD) show an opposite effect on CHN-1, whether the similar mutation in UFD-2 also show an opposite effect?
6. Figure 4C, 4 may not indicate the AHCY-1 di-Ub, whose MW should be ~70kD.
7. L463 worms treated with a proteasome inhibitor (MG132, 10μM) or DUB inhibitor (NEM, 100mM). Which lane was treated with MG132? And which one was NEM treated?
8. The ubiquitinated AHCY-1 in Figure 4D should be confirmed by AHCY-1 immunoprecipitation with ubiquitin blotting. And the overexpression of UFD-2, UFD-2 EEYD mutations in Figure 4D is helpful for further confirm AHCY-1 ubiquitination depend on the UFD-2 facilitated CHN-1 dimerization.

Dr. Hartmut Vodermaier
Senior Editor
EMBO Journal

Dear Dr. Vodermaier

Thank you for your positive response on our manuscript "Heterotypic Assembly Mechanism Regulates CHIP E3 Ligase Activity" **(EMBOJ-2021-109566).** We are pleased to read that the reviewers found our results interesting and important and that they thought the manuscript would appeal to a broad audience.

In response to the reviewers' comments, I have included previously unpublished results. Please find a detailed description of the edited paragraphs below (the reviewers' comments are **bold,** and our responses are *italic*).

Referee #1:
Major points:

1. The strength of the study is the range of approaches used and the evident attempt to link mechanistic insight to new physiologically relevant substrates. However, the breadth of the study is also a weakness as the physiological data is 'shoehorned' into a single figure and is not fully explored. I would prefer to see a structure orientated paper where the full range of E3 assays (from Fig 1) where carried out on the novel substrate and where the physiological study was perhaps left to a follow-up study where it could be more fully developed.

We are pleased that the Reviewer recognized the multiplicity of experimental approaches and the combination of in vitro data with the actual involvement of the E3 ligase complex in animal physiology. However, we feel that focusing only on in vitro study that tracks substrate modifications in multiple Ub reactions is somewhat limiting. In such a case, the reader will wonder what the de-regulation of AHCY-1 by the E3 complex will lead to and whether in vivo UFD-2 is indeed able to affect CHN-1 activity. Perhaps this last point is not sufficiently developed in the manuscript. So we intend to use more data related to CHN-1/UFD-2 interaction in vivo. Namely,

In addition, we will develop a section related to the ubiquitination and stability of AHCY-1. To this end, we will use worms we have generated with endogenous, fluorescent AHCY-1, whose localization, fluorescence intensity, and thus abundance we monitored (and will continue) by microscopy. We generated a worm line based on CRISPR/Cas9 knock-in of GFP to label AHCY-1 while maintaining its natural expression level. Thanks to this, we could track the sorting of AHCY-1 in a multi-tissue animal for the first time. Preliminary observation has established that AHCY-1 is mainly present in the nucleus of intestinal cells and the reproductive system. In contrast, the fluorescent fusion protein in worms with chn-1 depletion appeared to have higher intensity

INTERNATIONAL INSTITUTE OF MOLECULAR AND CELL BIOLOGY IN WARSAW
4 Ks. Trojdena Street, 02-109 Warsaw, Poland phone.: +48 22 597 07 00, fax: +48 22 597 07 15
secretariat@iimcb.gov.pl www.iimcb.gov.pl

throughout the whole organism than control RNAi. Direct down-regulation of AHCY-1::GFP (RNAi GFP).

Figure for Referees not shown

2. The writing of the manuscript feels rushed with the methodology and data not being fully explained - there are several instances where data is not referred to or where the methodology is poorly linked to the data.

The manuscript was prepared together with editors from Life Science Editors (lifescienceeditors.com), so I thought that its structure, descriptions were on the highest level. However, of course, I accept the Reviewer's opinion. Therefore, I will try to simplify or elaborate on parts of the text that relate to other Reviewer' comments.

3. Many of the quantitative statements in paragraph 1 of the results section are not supported by the methodology used or the lack of quantitation (e.g., densitometry), making use of the term significant (line 109) inappropriate.

The differences in signal intensities that we describe were so significant, easily seen in the figures, that we did not add densitometric quantifications to make Fig. 2 and EV1 too large. However, we obtained such measurements automatically by the Biorad detection system, which we use in WesternBlots imaging. I attach below one of such measurements, which the Reviewer asks for. We will include other quantifications of this type adjacent to other blots.

4 Ks. Trojdena Street, 02-109 Warsaw, Poland phone.: +48 22 597 07 00, fax: +48 22 597 07 15
secretariat@iimcb.gov.pl www.iimcb.gov.pl

Figure for Referees not shown

4. The ubiquitination assays are performed over a 90 min incubation period, and some of the reactions appear to have gone to completion, suggesting that they are not looking at stead state kinetics. In order to ensure that CHN-1 is rate limiting whilst also avoiding substrate depletion, which is clearly seen for example in Fig 1 lane 6, the length of the assay should have been optimise. In fact, it would have been appropriate to show a time course between 0-60 min.

We agree with the Reviewer that it is worth presenting a time course between 0-60 min on the block of Figure 1b, and we will prepare a new figure with more time points. However, we would like to point out that a similar analysis from the perspective of E2 enzyme discharging is already in EV2B, there are times 0, 5, 10, 20 and 30 min.

5. It is likely that optimisation would actually have made the results more definitive as the data presented over-estimate the relative activity of the CHN-1:UBE2D2(3) combination, compared to UBE2D1. Hence the statement on the ability of UBE2D2(3) to catalyse CHN-1 Ub with UFD-1 is misleading.

I attach quantifications showing that at fairly standard ubiquitylation reaction times for CHN-1 and CHIP (e.g., Tawo et al., 2017, Cell) we see significant increases in CHN-1 activity with the E2 enzyme UBE2D3 due to the presence of UFD-2 (Fig. 2). On densitometry quantifications (Fig. 2), we now also show the effect of UFD-2 on CHN-1 utilization of the UBE2D2 enzyme. This, along with a description, will be included in the revised version of the manuscript

6. It would also have been of interest to show the effect of titrating UFD-1 from a low stoichiometry to a 1:1 ration this would also support the model proposed in the later sections.

We agree with the Reviewer and we have such data showing that already at a ratio of 1uM (CHN-1): 0.3uM (UFD-2), the CHN-1 activity increases significantly (Fig. 3). We will prepare an appropriate panel with quantification.

Figure for Referees not shown

7. HD-MX: The study relies heavily on the HD-MX data to develop a model. The experiment was apparently performed in technical triplicates, but for only n=1. However, many journals are now holding authors to n=3, the reproducibility can be questioned with n=1 as HDX experiments are extremely sensitive to environmental conditions, like temperature, buffer components, ionic strength, exchange and quenching pH, digestion conditions. (The authors may wish to refer to: Masson et al. Recommendations for performing, interpreting and reporting HDX-MS experiments. Nat Methods 16; 595-60).

We do not quite agree with the requirement and regulations the Reviewer writes about. According to Masson et al., Recommendations for performing, interpreting, and reporting HDX-MS experiments. Nat Methods 16; 595-60:
"Repeated measurements of deuterium incorporation are necessary to ensure repeatability and deliver an estimate of the precision in the measurements. Independently generated exchange reactions serve as technical replicates. The same labeling reaction aliquoted and measured separately is not a suitable technical replicate, as this is not an independent observation. At a minimum, there should be at least three labeling reaction experiments performed for at least one time point to allow a reasonable estimate of the error of measured deuterium levels."

We have fulfilled this condition by performing measurements in all-time units as triplicates. We also followed published HDX measurements performed by industry leaders such as David Komander (Gladkova et al., 2018, Nature), where the measurement was performed as a technical triplicate of one biological repetition. And this is also, according to the guidelines (Masson et al. Nat Methods): "Biological replicates of the experiment should be conducted where possible." To perform another biological replicate, it costs almost 20.000 EUR if done by

4 Ks. Trojdena Street, 02-109 Warsaw, Poland phone.: +48 22 597 07 00, fax: +48 22 597 07 15
secretariat@iimcb.gov.pl www.iimcb.gov.pl

a company, or 7.000 EUR by a Core Facility of one of the Institute of National Science Academy in Poland. At this time, I am not collaborating with any other group specializing in HDX-MS. This is a costly and time-consuming method, and the result is likely to be related to the current one. However, if you think it is necessary to run another HDX-MS measurement, we will carry out such an analysis.

8. For example, the authors state that 'after quenching, the samples were frozen in liquid nitrogen and kept at -80{degree sign}C until mass spectrometry'. How long was the material stored at -80{degree sign}C, and does storage and rethawing impact on peptides recovered and/or back exchange, etc?

The material was stored at -80 ^{o}C overnight. Freezing step is an accepted method (Jia et al., 2020, Nature Comm.) and in previous studies of our collaborators on this manuscript indicated that freezing was found not to impact the results (Fatalska et al., 2021, ELife; Moysa et al., 2021, Structure).

9. Representation of the HD-data in EV2 and Fig 2 is poor and does not make it easy to assess the quality of the data or the authors interpretation. There seem to be peptides that are not deuterated in CHN-1 in the presence or absence of UFD-2. What was the % peptide cover for the undeuterated protein?

The X-axis represents the position in sequence for a peptide (the x value indicates the peptide length). The Y-axis presents the difference in fractional deuterium uptake with the Y-error bar indicating the uncertainty of the measurement from three independent replicates of the experiment. Positive values indicate stabilization of the region upon complex formation. Dotted lines indicate the confidence limit at 95% calculated using the Houde test (Houde et al., 2011). The upper and lower panels show results after 10 and 60 seconds of H/D exchange, respectively. Final data analysis was carried out and presented on EV2A figure using in-house HaDex software (Puchała et al., 2020). Values used by the HADEX tool to generate the figures (EV2A) can be used to generate a heat map corresponding to the level of a fraction of deuterium exchange. Since the values are numerical, we propose using these values and generating a gradient of colors to represent the extent of fraction exchange. See attached image (Fig. 4), were in the higher values or green and lower values red in color (gradient of red-white-green: representing lowest to the highest value of fraction exchange in that time point). An alternative is to have a gradient for that particular peptide across all the time points. We are also working to incorporate the heat map generation suite in the HADEX software, which we might use for such a representation. However, we can use a heat map liked by most but not showing the complete data. We have prepared a sample of such presentations, where we marked the differences in deuteration of CHN-1 in the presence of UFD-2. Eventually, we will remove the numerical values, they will be in the Excel file, and we will leave the color code. A similar color code will be applied to the model shown in Fig. 2A. We will also prepare a table with % of the peptide cover values.

4 Ks. Trojdena Street, 02-109 Warsaw, Poland phone.: +48 22 597 07 00, fax: +48 22 597 07 15
secretariat@iimcb.gov.pl www.iimcb.gov.pl

Figure for Referees not shown

10. If there is a discrepancy in undeuterated to deuterated peptide detection, it could indicate that the undetected/undeuterated peptides provide insights into the mechanism. Please provide information on the specific peptides not recovered.

Peptides "not covered" are marked in Fig 2. Peptides "not recovered" are different from "not deuterated," so it is difficult to understand what the Reviewer has in mind. However, we will prepare a different representation of these peptides and color code for an easier understanding of the model.

11. When the authors form the 1:1 complex, the sample is then diluted with deuterated buffer. What does this dilution do to the stability of the complex? For example, are you looking at re-binding after complex dissociation in response to dilution? In other words, early (10 sec or 60 sec) data may reflect changes as a consequence of dilution (i.e. the complex might not be at steady state in the first seconds of dilution). The point is, that HDX is a very dynamic method and what takes place 10 or 60 sec after a 10-20 fold dilution of a preformed complex is not clear. A longer HDX might reveal equilibrium effects. As the authors use a pre-formed complex they should show if the HD-MX is different when the binding partners are combined immediately prior to deuteration. Also, as peptide regulators are identified I am suppressed that the HD-MX analysis was not repeated with the activating peptide. The authors need to discuss the limitations of the study as well as possible protein dynamics effects resulting from the dilution/deuteration/time course method.

When CHN-1 deuteration uptake was recorded in the presence of UFD-2, CHN-1 alone was also being recorded. All the dilution and time courses were similar across this condition, with the only

4 Ks. Trojdena Street, 02-109 Warsaw, Poland phone.: +48 22 597 07 00, fax: +48 22 597 07 15
secretariat@iimcb.gov.pl www.iimcb.gov.pl

change being the addition of UFD-2. There is no other way to do HDX in native-like conditions than dilute. This applies to all HDX experiments. The scenario might be that the complex becomes destabilized upon dilution, and with incubation time, we have less complex, but still, the observed differences indicate qualitatively what is going on in the complex or no complex at all, then we see no differences. If complex disappears in the incubation timeframe, we would observe a decrease of differences at longer incubation time and not at shorter inc times. The slow dissociation would change quantitative results (i.e., the degree of change in the protection of a given region) - not qualitative (i.e., are there changes in a given region or not). Stability upon dilution depends on numbers, i.e., KD and koff. It is widely accepted that in routine HDX, the differences observed between complex and partners alone are indicative of binding events or their allosteric consequences, irrelevant to the complex's stability. A negative experiment, no differences, could be interpreted as possibly coming from complex dissociation, but not if we have positively detected changes. We only can have "less complex" and no "more complex" or "different complex" upon dilution.

Mixing "just prior" to exchange would make sense if we can estimate what "just prior" means. We will discuss the limitations of the method, i.e., dilution, as described above.

12. The HD-MX data is over interpreted and poorly explained in the sections between lines 160-173. I would suggest presenting the figures in a way that helps the reader to get the most out of the data, for example using a more detailed colour code to map % D2O onto the structure. I also think the HD-MX study could be extended (as suggested above) and strengthened using additional experimental approaches to support the authors claims.

In response to the Reviewer's concerns, we have re-analyzed the raw data to generate a model with a more accurate representation of the changes occurring in the CHN-1 structure and describe and visualize the details of these changes. However, we only will on the model and discuss the effects of convincing statistical relevance. Appropriately modified panels will be prepared in the revised manuscript.

13. Fig 2B: Why is the format of the data suddenly changes? In order to interpret the data, the ubiquitination also needs to be shown.

We have the entire blot (Fig. 5a). Because of the repetitions concerning the poly-Ub observations, we have removed this part of the blot to focus the reader's attention on the non-modified CHN-1 signal, which is the subject of quantification. In the revised version, we will show the complete detection.

4 Ks. Trojdena Street, 02-109 Warsaw, Poland phone.: +48 22 597 07 00, fax: +48 22 597 07 15
secretariat@iimcb.gov.pl www.iimcb.gov.pl

Figure for Referees not shown

14. Fig 2C: The data and explanation of this figure is inadequate. The experiment is designed to detect Ub, when it would make much more sense to detect the E2 component, so that the ratio of charged and uncharged E2 could be determined.

We do not think that the explanation of the results is inadequate since we are evaluating the process, rate, and amount of Ub chains formation, whose only source is E2-Ub. However, we will prepare a detection of this blot using anti-E2 antibodies.

The UBE2D1Ub band assignment has not been validated by including a UBE2D1Ub plus reducing agent control.

UBE2D1Ub band assignment was validated in Fig. 3E, where we show the inability of CHN-1/HSP-1 to discharge E2 in many time points in contrast to other assays performed the same way CHN-1 is functional. This allowed us to assess that the discharged system was adequately set up and matched our assumptions. However, we understand that we could have included controls with the reducing factor. We will repeat these analyses with this control.

E2 discharge assays are notoriously sensitive to pH, temperature, etc and are known to discharge over time in the absence of E3, a buffer only control over the course of the experiment is therefore essential.

E2 discharged assay was validated in Fig. 3E, where we show the inability of CHN-1/HSP-1 to discharge E2 in many time points.

It would have added to the figure to show a titration of CHN-1 and UFD-2 with fixed E2. There are similar issues with EV2B.

INTERNATIONAL INSTITUTE OF MOLECULAR AND CELL BIOLOGY IN WARSAW
4 Ks. Trojdena Street, 02-109 Warsaw, Poland phone.: +48 22 597 07 00, fax: +48 22 597 07 15
secretariat@iimcb.gov.pl www.iimcb.gov.pl

We will perform such an experiment.

15. I may have missed it but I can't find any reference to technical replicates in the MS methodology. I would expect to see technical triplicates for each biological replicate as standard.

That was the case here. Moreover, due to the pooling of thousands of worms, the biological variability was reduced to a minimum within one sample and the observed variance is due to technical variability. We will include this information in the text and emphasize it more in the material and methods section.

16. Fig 4D: The AHCY-1 portion of the immunoblot is over-exposed (especially when compared to the tubulin control); it should be redone and quantified using densitometry to determine the relative levels tubulin. However, by eye it seems fairly evident that AHCY1 levels are decreased in the chn-1 KO relative to the WT and flag controls (in relation to tubulin).

We normalized the signal using tubulin, the values are shown and quantification is shown below (Fig. 6).

Figure for Referees not shown

4 Ks. Trojdena Street, 02-109 Warsaw, Poland phone.: +48 22 597 07 00, fax: +48 22 597 07 15
secretariat@iimcb.gov.pl www.iimcb.gov.pl

This is not unprecedented as CHIP has been identified as both a positive and negative regulator of the steady state levels of some substrates dependent on cell stress conditions. Despite not seeing a 'stabilisation' (i.e. increase in steady state levels) of the AHCY-1 protein (by immunoblot) with loss of CHN-1 function the authors still interpret the lipidomics in terms of AHCY-1 stability - presumably on the basis of the proteomics. However, caution is required during the interpretation of LC-MS/MS data as it reflects differences in the abundance of ionized peptides and not necessarily differential protein steady state levels. The abundance of ionized peptides can be influenced by a combination of factors, including post-translational modifications, inter-molecular interactions and compatibility with MS detection (ionization and fragmentation potentials).

We agree with the Reviewer that the detected protein level in LC-MS/MS can be influenced by post-translational modification. Nevertheless, due to the low occupancy of most PTMs, the unmodified peptide abundance remains often unchanged. Additionally, for the protein AHCY-1, we detected 50 unique peptides in total, providing a solid base for the quantification. We will perform heat maps of an abundance of every AHCY-1 peptide detected by MS.

17. Discussion: The discussion is a bit limited and is treated more as a summary of the authors own findings than a forum for a more critical analysis of the data in respect to the literature.

I thank the Reviewer for pointing out the need for more critical discussion. I will reformat the discussion accordingly in the revised manuscript.

Other comments:
• The text states that assays with worms plus MG132 and NEM are shown but I do not see the data?

This information is included in the Fig. 4 legend of the original manuscript.

• Fig EV1C: It appears that the exposure time in figure EV1C is a lot shorter than in other figures (compare for example the unmodified band in Fig EV1F) to which it is being compared. Never the less it is possible to discern bands that are present in the plus UFD-2 lane compared to the minus UFD-2 lane. There should be a positive control present on the immunoblot that allows comparison with other gels and a comment should be made about these bands.

We have such a blot, with more prolonged exposure and control (Fig. 7). In order to better determine the nature of these barely visible signals, we will carry out a control reaction of the ubiquitination of CHN-1 H218Q by the UFD-2 mutant P951A. At this point, we can potentially speculate about the potentially minimal intrinsic activity of the CHN-1 H218Q mutant.

4 Ks. Trojdena Street, 02-109 Warsaw, Poland phone.: +48 22 597 07 00, fax: +48 22 597 07 15
secretariat@iimcb.gov.pl www.iimcb.gov.pl

Figure for Referees not shown

• There is no plus UFD-2 minus CHN-1 control in figure EV1B.

In the figure EV1A, we have shown that UFD-2 could not utilize the UBE2N/Uev1a enzyme, so we thought of omitting that as a control in the EV1B figure. Nevertheless, we can experiment and place such a control (although this result, as I mentioned, is on EV1A).

• The authors should comment on the fact that addition of UFD-2 to DTPR-CHN-1 actually decreases the amount of Ub detected.

We have not been able to determine the cause of effectively comment on the slight reduction in the signal that the Reviewer writes about. Based on the high activity data of UFD-2 itself (EV1A) and other observations, we can speculate that UFD-2 utilized most of the Ub for the auto-Ub reaction, thereby slightly reducing its availability to the slow-operating CHN-1 mutant. We can include such information in the text, although it adds little to the mechanism.

• S3F (line 242 and 245) should be EV3F, there are several other places where EV is replaced with S.

We will make the appropriate corrections to the text.

• Fig EV4G: A minus E3 control needed.

We have this blot with the required control (Fig. 8). Because of our mistake, we did not put entire blot on the figure.

4 Ks. Trojdena Street, 02-109 Warsaw, Poland phone.: +48 22 597 07 00, fax: +48 22 597 07 15
secretariat@iimcb.gov.pl www.iimcb.gov.pl

Figure for Referees not shown

• Fig 4C: K48 and K63 are used in the assay but the data do not appear to be referred to in the text and should therefore be removed from the figure.

Indeed, we did not clearly explain why we decided to use these two Ub mutants to analyze the ubiquitination of AHCY-1. We wanted to determine whether the Ub chains generated by CHN-1/UFD-2 are monotonic (Lys48 or 63 only) or mixed type. This result indicates a significantly divergent poly-Ub pattern using wild-type Ub, in contrast to Lys48 and 63 Ub mutants, suggesting ubiquitination of AHCY-1 by mixed-type chain types. We think this is an interesting observation and will incorporate an appropriate description into the text.
In addition, to document the type of chains generated by the UBE2D1/CHN-1 E2/E3 pair, we conducted autoubiquitylation assays with ubiquitin variants bearing individually mutated lysine residues ("K-to-R" mutants). It was demonstrated that UBE2D1 preferentially generates K-11, -29, -48, and -63 poly-Ub chains (Windheim et al., 2008; Wang et al., 2006; Bosanac et al., 2011). We noted that UBE2D1 efficiently generated chains made of ubiquitin, missing the side chains of lysine 11, 29, 33, or 48, but not 6 and 63 (Fig. 9, right panel). We wondered if the interaction with UFD-2 could alter the type of polyubiquitin chains generated by CHN-1 and UBE2D1. The presence of UFD-2 in the reaction significantly increases the efficiency of CHN-1 ubiquitylation with all ubiquitin mutants. However, the preferences for chain linkage-type were not changed by UFD-2 (Fig. 9, left panel). Taken together, our results suggest that UFD-2 binding to CHN-1 did not influence the specificity of CHN-1 driven ubiquitylation.

4 Ks. Trojdena Street, 02-109 Warsaw, Poland phone.: +48 22 597 07 00, fax: +48 22 597 07 15
secretariat@iimcb.gov.pl www.iimcb.gov.pl

Figure for Referees not shown

Referee #2

Major concerns:

1. It is very interesting that UFD-2 stabilizes CHN-1 U-box dimer, whereas the authors only show the HDX-MS results. Size exclusion chromatography profiles of CHN-1 alone, CHN-1 and UFD-2 (or UFD-2 PEPTIDE2) may be helpful.

We performed SEC analysis, but a clear interpretation of these data was impaired because of the tendency of CHN-1 to form oligomers, resulting in multiple peaks in the SEC chromatogram. Thus, the SEC chromatogram of CHN-1 alone gave a large peak corresponding to the oligomeric population of CHN-1 followed by a peak in the elution volume of its dimer (one example in Fig. 10). Accordingly, for each in vitro experiment, we produced fresh CHN-1 and worked with the dimer fraction only.

Figure for Referees not shown

4 Ks. Trojdena Street, 02-109 Warsaw, Poland phone.: +48 22 597 07 00, fax: +48 22 597 07 15
secretariat@iimcb.gov.pl www.iimcb.gov.pl

Interestingly, when we mixed both E3 ligases (equal molar ratio) and subjected them to SEC, we obtained two different peaks corresponding to the respective proteins (Fig. 11), suggesting a shift toward stabilizing the CHN-1 dimer population by UFD-2. This observation indicates that UFD-2 introduces some changes in the conformational stability of CHN-1 and prompted us to use other techniques (MDS and HDX MS). Previously, we did not demonstrate this because our efforts to form a stable complex (CHN-1 and UFD-2) on SEC failed, indicating a dynamic/transient complex that may shift attention from the functional significance of the complex of the two E3 ligases toward the structural part.

Figure for Referees not shown

4 Ks. Trojdena Street, 02-109 Warsaw, Poland phone.: +48 22 597 07 00, fax: +48 22 597 07 15
secretariat@iimcb.gov.pl www.iimcb.gov.pl

In addition, they have mapped the binding regions in UFD-2 EEYD, whether the mutation in this region (UFD-2 EEYD mutations) could abolish the function of UFD-2 in CHN-1 U-box dimer stabilization and ubiquitylation activity?

We did not include this result earlier because purification of such a mutant of UFD-2 (EEVD) proved complicated. We recently obtained a small fraction and have since performed the experiment that Reviewer 2 writes about. Indeed, such a mutant of UFD-2 (EEVD) is not able to induce CHN-1 activity like its wild-type form (UFD-2) (Fig. 12). We will prepare the corresponding figure in the revised manuscript.

Figure for Referees not shown

2) The authors claim that UFD-2 stabilizes the U-box dimer with two E2 sites, whereas their results could not sufficiently support this major point, more evidences are definitely needed to support this conclusion.

We agree, and we have prepared an appropriate experiment, but it needs some clarification as it involves CHN-1/CHIP monomerization, on which we have a manuscript in revision in Mol Cell. Namely, in the other manuscript, we show that while the CHIP/CHN-1 dimer promotes chaperone-directed protein quality control, the CHIP/CHN-1 monomer regulates chaperone-independent turnover of the insulin receptor (INSR) and longevity. To prevent CHN-1 dimer formation, we mutated the conserved hydrophobic residue Leu141 to arginine (L141R), which disrupts the dimer interface of the CC domain producing a stable monomer with the activity of a single U-box domain. To determine if CHN-1 U-box dimerization is necessary for the UFD-2 dependent activity boost, we analyzed the ubiquitylation of the CHN-1 ^{L141R} mutant incapable of dimerization. Indeed, the CHN-1 monomer showed no change in ubiquitination activity in the presence of UFD-2 (though it interacts), indicating that both U-box dimerization is essential for UFD-2 dependent processivity regulation (Fig. 13).

4 Ks. Trojdena Street, 02-109 Warsaw, Poland phone.: +48 22 597 07 00, fax: +48 22 597 07 15
secretariat@iimcb.gov.pl www.iimcb.gov.pl

Figure for Referees not shown

First, they showed UBE2D1GST-Ub (while they have not described how to produce ubiquitin-charged GST-UBE2D1 in the methods or legends) could directly bind to CHN-1, however, UFD-2(P951A) could not enhance the amount of CHN-1 in the immunoprecipitate in Figure EV1E. They should further confirm that CHN-1/UFD-2 could bind with two E2.

Charged E2 were obtained commercially (BostonBiochem). We will place an appropriate description in M&M. Based on IP experiments, it is difficult to readily interprete the amount of E2 bound by CHN-1 because IP is a non-equilibrium system. Nevertheless, we have other data to strengthen our conclusions apart from the ones presented in Figure 13. We carried out Ub reactions in a system with two different E2 enzymes with different specificities of Ub chain formation. Here, the presence of UFD-2 enables the formation of Ub patterns of both E2

4 Ks. Trojdena Street, 02-109 Warsaw, Poland phone.: +48 22 597 07 00, fax: +48 22 597 07 15
secretariat@iimcb.gov.pl www.iimcb.gov.pl

enzymes at once. This can be seen in the example of UBE2D1 and Uev1a/UBE2V1 (Figure 14). The revised manuscript will also use these data.

Figure for Referees not shown

Second, authors showed UFD-2 (P951A) could dramatically enhance the autoubiquitylation of CHN-1 by using equal amount of UBE2D1 in Figure 2B, whereas the effective control groups are loss. The half and 2-fold of UBE2D1 in Figure 2B lane 6 should be used in the CHN-1/UFD-2 ubiquitination assay.

We can repeat this experiment using even lower concentrations of E2. However, a similar analysis is shown in Fig. 2C, so we do not think it is necessary to repeat the experiment shown in Fig.2B. Besides, we show the effect of UFD-2 P951A on CHN-1 activity in many other panels.

Third, the E2-discharging assay is problematic. The discharging reaction samples should also be treated with reducing agent and detected with UBE2D1 blotting to further confirm the UBE2D1-Ub bands. In addition, the auto ubiquitylated CHN-1 and Di-Ub should be observed to reflect the activity of CHN-1.

UBE2D1Ub band assignment was validated in Fig. 3E, where we show the inability of CHN-1/HSP-1 to discharge E2 in many time points in contrast to other assays performed the same way, where CHN-1 is functional. This allowed us to assess that the discharged system was adequately set up and matched our assumptions. However, we understand that we could have included controls with the reducing factor. We will repeat these analyses with this control. We also have the di-Ub data for this experiment along with quantification (Fig. 15) and will include them in the revised manuscript.

4 Ks. Trojdena Street, 02-109 Warsaw, Poland phone.: +48 22 597 07 00, fax: +48 22 597 07 15
secretariat@iimcb.gov.pl www.iimcb.gov.pl

Figure for Referees not shown

Figure 15. E2 discharging assay of Ub-charged UBE2D1 in the presence of CHN-1/UFD-2^{P951A}. The reaction was stopped after the indicated time via heat inactivation in native conditions. Protein samples were resolved via SDS-PAGE and immunoblotted with anti-Ub antibodies. Quantified the poly-ub signal and di-ubiquitin signal that appeared from each lane. Representative graph, where Y-axis plotted against the intensity of the signal from each lane and X-axis plotted against the µM concentration of UBE2D1-Ub.

3. As there are some reported U-box:E2 structure (PMID: 20696396, 18485199, 20146531, 16307917), the authors could dock them with the stabilized CHN-1 dimer to further support their statements.

We have performed the preliminary docketing requested by Reviewer 2. We will expand on this analysis in the revised manuscript.

Figure for Referees not shown

4 Ks. Trojdena Street, 02-109 Warsaw, Poland phone.: +48 22 597 07 00, fax: +48 22 597 07 15
secretariat@iimcb.gov.pl www.iimcb.gov.pl

4. Given that HSP-1 could diminish the activity of CHN-1/UFD-2 and HSP-1 shows a higher binding affinity with CHN-1 than UFD-2, whether HSP-1 could compete with UFD-2 for binding CHN-1?

We performed an ELISA experiment to test the competition of UFD-2 by HSP-1 (or DAF-21) for CHN-1 and found that HSP-1, which has a higher affinity for CHN-1, could detach it from UFD-2 immobilized on the ELISA plate (Fig. 16). Below I included a schematic representation of the ELISA titration assay to test whether chaperones compete for CHN-1 bound to immobilized UFD-2.

Figure for Referees not shown

By immunoprecipitation assays, we also showed that HSP-1 interfered with the interaction of CHN-1 with UFD-2 (Fig. 17). Additionally, induction of E2 discharging by UFD-2 was blocked by the presence of HSP-1 in the system. These results indicate competence between HSP-1 and UFD-2 for CHN-1 and higher affinity of HSP-1.

INTERNATIONAL INSTITUTE OF MOLECULAR AND CELL BIOLOGY IN WARSAW
4 Ks. Trojdena Street, 02-109 Warsaw, Poland phone.: +48 22 597 07 00, fax: +48 22 597 07 15
secretariat@iimcb.gov.pl www.iimcb.gov.pl

Figure for Referees not shown

4) AHCY-1 is a newly identified substrate for CHN-1/UFD-2, whereas the protein level of AHCY-1 is not consistent in MS data and Western blot in Figure 4A and 4D. As the authors claim that assembly with UFD-2 enables CHIP/CHN-1 to regulate lipid metabolism by ubiquitylation of AHCY-1, the UFD-2 EEYD mutations should be provide as negative control in both AHCY-1 ubiquitination assay and the lipid examination.

We will use the UFD-2 EEVD mutant in an in vitro AHCY-1 ubiquitination reaction. However, based on the lack of stimulatory effect of this mutant on CHN-1 (Fig. 12), we can predict the result of the lack of effect of UFD-2 EEVD on Ub AHCY-1. To extend this issue and ultimately show the relevance of the EEYD motif in the UFD-2 protein, we will generate worms with a point mutation in UFD-2 and perform an analysis of AHCY-1 ubiquitylation and lipidomics.

Minor comments:

1. Figure 1A, why the authors do not used the E2 of CHN-1 in C. elegans (UBC-2/Let-70, PMID: 15294159)?

We had difficulty obtaining recombinant and active LET-70. Therefore, we used (purchased from Bostonbiochem) the LET-70 orthologs, UBE2D1-3, and used them in our analyses as previously performed in a publication Tawo et al., 2017, Cell. Nevertheless, we will try re-expression and other conditions to purify LET-70 to use it in an in vitro-Ub CHN-1 and UFD-2 control reaction.

2. Figure 1B, time-dependent auto-Ub experiment sounds not work, the authors should shorten time.

We agree with the Reviewer that it is worth presenting a time course between 0-60 min on the block of Figure 1b, and we will prepare a new figure with more time points. However, we would

4 Ks. Trojdena Street, 02-109 Warsaw, Poland phone.: +48 22 597 07 00, fax: +48 22 597 07 15
secretariat@iimcb.gov.pl www.iimcb.gov.pl

like to point out that similar analysis from the perspective of E2 enzyme discharging is already in figure EV2B, there are times 0, 5, 10, 20 and 30 min.

3. Figure EV1C, CHN-1 signal could be observed in lane without CHN-1?

In the lane without CHN-1, we did not record any background signals based on anti-CHN-1 antibodies (Fig. 3), so we did not use such control in EV1C. We will apply such a control in the revised manuscript.

4. Figure 2B, C, as the authors state that immunoblots representative of n = 3 experiments are shown, statistical analysis could be performed. Same things in Figure 3B, 3C and 4C.

We will perform the statistical analysis that the Reviewer asks for.

5. It is very interesting that a single point mutation in HSP-1 (EEVD to EEYD) show an opposite effect on CHN-1, whether the similar mutation in UFD-2 also show an opposite effect?

We did not include this result earlier because purification of a mutant of UFD-2 (EEVD) proved to be complicated. We recently obtained a small fraction and have since performed the experiment that Reviewer 2 writes about. Indeed,the UFD-2 EEVD mutant is not able to induce CHN-1 activity like UFD-2 wild-type protein. We will prepare the corresponding figure in the revised manuscript.

Figure for Referees not shown

4 Ks. Trojdena Street, 02-109 Warsaw, Poland phone.: +48 22 597 07 00, fax: +48 22 597 07 15
secretariat@iimcb.gov.pl www.iimcb.gov.pl

6. Figure 4C, 4 may not indicate the AHCY-1 di-Ub, whose MW should be ~70kD.

The migration of ubiquitylated proteins rarely correlates with the calculation of their mass-dependent distribution on the gel. In this case, however, it is di-Ubiquitylation. To establish this, we have performed the AHCY-1 ubiquitylation reaction using a Ub (Ub-NoK) mutant, which cannot form Ub chains (only monoubiquitylation). As can be seen below (Fig. 18), these two signals close to each other with a mass relative to the marker of less than 70kDa come from di-Ub AHCY-1. We will use this blot in EV4 figure.

Figure for Referees not shown

7. L463 worms treated with a proteasome inhibitor (MG132, 10µM) or DUB inhibitor (NEM, 100mM). Which lane was treated with MG132? And which one was NEM treated?

This information is contained in the legend of the figure. Yes, all samples were treated with MG132 and NEM.

8. The ubiquitinated AHCY-1 in Figure 4D should be confirmed by AHCY-1 immunoprecipitation with ubiquitin blotting. And the overexpression of UFD-2, UFD-2 EEYD mutations in Figure 4D is helpful for further confirm AHCY-1 ubiquitination depend on the UFD-2 facilitated CHN-1 dimerization.

We agree that preci[itation of AHCY-1 and then detecting Ub is worthwhile. We have already performed a preliminary experiment but using ubiquitin as bait. Interestingly, we were able to pull down significantly less AHCY-1 in worms lacking UFD-2 (ufd-2 tm1380 animals), a situation where CHN-1 cannot effectively ubiquitinate AHCY-1 (Fig. 19). We will further develop this experiment and include the results in a revised version of the manuscript.

4 Ks. Trojdena Street, 02-109 Warsaw, Poland phone.: +48 22 597 07 00, fax: +48 22 597 07 15
secretariat@iimcb.gov.pl www.iimcb.gov.pl

Figure for Referees not shown

We will also generate worms that produce the UFD-2 EEVD mutant, overexpress UFD-2 and examine the stability of AHCY-1/Ub.

4 Ks. Trojdena Street, 02-109 Warsaw, Poland phone.: +48 22 597 07 00, fax: +48 22 597 07 15
secretariat@iimcb.gov.pl www.iimcb.gov.pl

1st Editorial Decision **21st Oct 2021**

Thank you for your detailed tentative response to the referee reports, and proposal for revising this work for The EMBO Journal. I have now had a chance to consider these plans, and realize that the technical and presentational issues raised especially by referee 1 should be potentially satisfied by the planned modifications; this includes my agreement with your argumentation against the necessity of additional repetitions of the MS-HDX experiments.
I also appreciate your proposals for addressing referee 2's concerns, including the generation and analysis of UFD-2 mutant/overexpressing worms, and would therefore be happy to grant an extended revision period in this case. Our 'scooping protection' (meaning that any competing manuscript published elsewhere during revision will have no negative impact on our final decision on your study) will of course remain valid during this revision.

In conclusion, we shall be happy to consider a revised manuscript further for EMBO Journal publication, and would like to herewith formally invite you to prepare and resubmit a new version, modified and extended as proposed in your draft response. I should remind you that it is our policy to allow only a single round of (major) revision, making it important to carefully revise and answer all points raised to the referees' satisfaction at this point.

Additional information and more detailed guidelines on how to prepare a revision can be found below and in our online Guide to Authors. Please particularly note the possibility to add up to five Expanded View figures (which will be typeset and directly accessible in the HTML version), and the option to include up to eight or nine main figures as well, in order to make the presentation as accessible and clear as possible.

Thank you again for the opportunity to consider this work, and I look forward to your revision!

Please find a detailed description of the edited paragraphs below (the reviewers' comments are **bolded,** and our responses are in *italics*).

Referee #1:
Major points:

The strength of the study is the range of approaches used and the evident attempt to link mechanistic insight to new physiologically relevant substrates. However, the breadth of the study is also a weakness as the physiological data is 'shoehorned' into a single figure and is not fully explored. I would prefer to see a structure orientated paper where the full range of E3 assays (from Fig 1) where carried out on the novel substrate and where the physiological study was perhaps left to a follow-up study where it could be more fully developed.

We are pleased that the Reviewer recognized the multiplicity of experimental approaches and the combination of in vitro data with the real-world involvement of the E3 ligase complex in animal physiology. We believe that the physiological relevance adds depth to this story and establishes a starting point for further studies of the regulation and function of the CHN-1–UFD-2 interaction, which is of interest to a wide range of EMBO Journal readers. However, we agree with the Reviewer's suggestion to expand this part of the manuscript and analyze the role of the CHN-1–UFD-2 complex in proteostasis regulation. We have prepared a new figure (Fig. 4) showing that the interaction with UFD-2 also induces CHN-1 turnover (presumably via enhanced CHN-1 self-Ub) and affects proteostasis and worm recovery from proteotoxic stress.

In addition, we further developed a section related to the ubiquitylation and stability of AHCY-1 (Fig. 5B-D and EV4H-J). Since we did not observe a change in the stability of unmodified AHCY-1 in worm lysates, which could be related to the tendency of AHCY-1 to precipitate during sample preparation (Fig. EV4K), we generated a CRISPR/Cas9 knock-in GFP-based worm line to label AHCY-1 to track its localization and abundance without compromising the integrity of the worms while maintaining its natural expression level. The GFP tag did not affect AHCY-1 functionality, as its knockout is lethal to worms (WormBase and our observations). Next, we crossed chn-1(by155), ufd-2(tm1380), and CHN-1 OE worms with animals expressing GFP::AHCY-1. Microscopic analysis of GFP::AHCY-1 fluorescence levels revealed a significant increase in chn-1(by155) and ufd-2(tm1380) null allele worms and a decrease when CHN-1 is overexpressed (Fig. 5D). Quantitative PCR analyses showed no statistically significant changes in the AHCY-1 transcript levels (Fig. EV4J), suggesting that the increase in AHCY-1 levels is posttranslationally regulated by CHN-1 and UFD-2.

Many of the quantitative statements in paragraph 1 of the results section are not supported by the methodology used or the lack of quantitation (e.g., densitometry), making use of the term significant (line 109) inappropriate. The ubiquitination assays are performed over a 90 min incubation period and some of the reactions appear to have gone to completion suggesting that they are not looking at stead state kinetics. In order

INTERNATIONAL INSTITUTE OF MOLECULAR AND CELL BIOLOGY IN WARSAW
4 Ks. Trojdena Street, 02-109 Warsaw, Poland phone.: +48 22 597 07 00, fax: +48 22 597 07 15
secretariat@iimcb.gov.pl www.iimcb.gov.pl

to ensure that CHN-1 is rate limiting whilst also avoiding substrate depletion, which is clearly seen for example in Fig 1 lane 6, the length of the assay should have been optimise. In fact, it would have been appropriate to show a time course between 0-60 min. It is likely that optimisation would actually have made the results more definitive as the data presented over-estimate the relative activity of the CHN-1:UBE2D2(3) combination, compared to UBE2D1. Hence the statement on the ability of UBE2D2(3) to catalyse CHN-1 Ub with UFD-1 is misleading. The time-course for Ub (60-90min; Fig 1B) is again a strange choice as the reaction has clearly gone to completion and the substrate (in this case CHN-1) has been depleted at the first time point (60 min) under some conditions. The time course should cover shorter time point to make the data more quantitative allowing estimation of relative differences in autoubiquitination activity.

We agree with the Reviewer and prepared new figures with optimized time-course experiments (Fig. 1A, EV1G, and EV1H).

It would also have been of interest to show the effect of titrating UFD-1 from a low stoichiometry to a 1:1 ration this would also support the model proposed in the later sections.

We performed UFD-2 titrations (0.3, 0.65, and 1.3 µM) with a fixed concentration of CHN-1 (1.3 µM). We observed an almost 2-fold increase in CHN-1 auto-Ub in the presence of 0.65 µM UFD-2, which roughly translates to one CHN-1 dimer per one UFD-2 monomer (Fig. 1B). At higher UFD-2 concentrations, we did not observe any further increase in CHN-1 ubiquitylation; however, this effect could also be related to a Ub shortage in the reaction buffer, as the available Ub is robustly consumed by UFD-2 for its own auto-Ub.

HD-MX: The study relies heavily on the HD-MX data to develop a model. The experiment was apparently performed in technical triplicates, but for only n=1. However, many journals are now holding authors to n=3, the reproducibility can be questioned with n=1 as HDX experiments are extremely sensitive to environmental conditions, like temperature, buffer components, ionic strength, exchange and quenching pH, digestion conditions. (The authors may wish to refer to: Masson et al. Recommendations for performing, interpreting and reporting HDX-MS experiments. Nat Methods 16; 595-60).

We are pleased to explain this concern.
According to Masson et al., Recommendations for performing, interpreting, and reporting HDX-MS experiments. Nat Methods 16; 595-60:
"Repeated measurements of deuterium incorporation are necessary to ensure repeatability and deliver an estimate of the precision in the measurements. Independently generated exchange reactions serve as technical replicates. The same labeling reaction aliquoted and measured separately is not a suitable technical replicate, as this is not an independent observation. At a minimum, there should be at least three labeling reaction experiments performed for at least one time point to allow a reasonable estimate of the error of measured deuterium levels."

4 Ks. Trojdena Street, 02-109 Warsaw, Poland phone.: +48 22 597 07 00, fax: +48 22 597 07 15
secretariat@iimcb.gov.pl www.iimcb.gov.pl

We have fulfilled this condition by performing measurements across all time points as triplicates. We also followed published HDX measurements performed by leaders in the ubiquitin ligase field like David Komander (Gladkova et al., 2018, Nature), where the measurement was performed as a technical triplicate of a single biological repeat. This is also in agreement with the guidelines (Masson et al. Nat Methods): “Biological replicates of the experiment should be conducted where possible”. Performing another biological replication would exceed our budget, as the comercial cost of the whole experiment is around 17,000 EUR. I am not currently working with any group specializing in HDX-MS that could perform measurements for us in a non-remunerated, collaborative manner.

For example, the authors state that 'after quenching, the samples were frozen in liquid nitrogen and kept at -80{degree sign}C until mass spectrometry'. How long was the material stored at -80{degree sign}C, and does storage and rethawing impact on peptides recovered and/or back exchange, etc?

The material was stored overnight at - 80 °C. Freezing is an accepted method (Jia et al., 2020, Nature Comm.), and previous studies by the group performing HDX-MS analysis in this study have shown no effect of freezing on measurements (Fatalska et al., 2021, eLife; Moysa et al., 2021, Structure).

Representation of the HD-data in EV2 and Fig 2 is poor and does not make it easy to assess the quality of the data or the authors interpretation. There seem to be peptides that are not deuterated in CHN-1 in the presence or absence of UFD-2. What was the % peptide cover for the undeuterated protein? If there is a discrepancy in undeuterated to deuterated peptide detection, it could indicate that the undetected/undeuterated peptides provide insights into the mechanism. Please provide information on the specific peptides not recovered.

We prepared a new visualization based on HaDeX software (Puchała et al., 2020) as a differential chicklet plot showing the differences in deuterium uptake by CHN-1 peptides due to the presence of UFD-2 across the five time points. The values are presented for each peptide (identified by its ID, Supp. Table) in the time course of the experiment in the form of a tile. Differential values are represented by the intensity of color, with blue indicating the more extensive uptake and red indicating less uptake in the second state (CHN-1 in complex with UFD-2). The gray color indicates a lack of data for a peptide in the second state. The cross sign represents the uncertainty - the bigger the cross, the more considerable the uncertainty, as stated in the plot legend (total of 99 peptides, 84,2% sequence coverage, 4.55 redundancy) (Fig. EV2B). We have also improved the visualization of the CHN-1 structural model (Fig. 2A).
We detected 99 peptides with 84.2% sequence coverage of CHN-1. Some discrepancies in the profile of detected peptides between the two conditions (CHN-1 alone and CHN-1 + UFD-2) are shown in the source data for Figures 2 and EV2B. In this table, peptides with missing "Frac Diff DU %" values are CHN-1 peptides that were not detected when CHN-1 was complexed with UFD-2. However, most of these were

4 Ks. Trojdena Street, 02-109 Warsaw, Poland phone.: +48 22 597 07 00, fax: +48 22 597 07 15
secretariat@iimcb.gov.pl www.iimcb.gov.pl

redundant with other detected peptides, except for the "NNLKMT" peptide, which extends from the 52nd to 57th residue on CHN-1 and is a linker between the pair of antiparallel alpha helices of the 2nd TPR in CHN-1. This may indicate that CHN-1 interacts with UFD-2 through this region. However, this peptide was not detected over the entire time range of the HDX-MS experiment and therefore its role in the CHN-1-UFD-2 complex cannot be determined. We also realize that peptide deuteration coverage is limited to the detectors of the measurement device and the nature of the peptides. This causes the experimental detection to differ from the theoretical peptide list.

When the authors form the 1:1 complex, the sample is then diluted with deuterated buffer. What does this dilution do to the stability of the complex? For example, are you looking at re-binding after complex dissociation in response to dilution? In other words, early (10 sec or 60 sec) data may reflect changes as a consequence of dilution (i.e. the complex might not be at steady state in the first seconds of dilution). The point is, that HDX is a very dynamic method and what takes place 10 or 60 sec after a 10-20 fold dilution of a preformed complex is not clear. A longer HDX might reveal equilibrium effects. As the authors use a pre-formed complex they should show if the HD-MX is different when the binding partners are combined immediately prior to deuteration. Also, as peptide regulators are identified I am suppressed that the HD-MX analysis was not repeated with the activating peptide. The authors need to discuss the limitations of the study as well as possible protein dynamics effects resulting from the dilution/deuteration/time course method.

We are pleased to explain this concern. When CHN-1 deuteration uptake was recorded in the presence of UFD-2, uptake of CHN-1 itself was also recorded. All the dilution and time courses were similar across this condition, with the only change being the addition of UFD-2. There is no other way to do HDX-MS in native-like conditions than dilute. The scenario might be that the complex becomes destabilized upon dilution, and with incubation time, we have less complex, but still, the observed differences indicate qualitatively changes in CHN-1 upon UFD-2 interaction. Mixing "just prior" to exchange would make sense if we could estimate "just prior". If the complex dissociated in the incubation timeframe, we would observe a decrease of differences at longer incubation times and not at shorter incubation times. The slow dissociation would change the quantitative results (i.e., the degree of change in the protection of a given region) - not qualitative (i.e., changes in a given region or not). Stability upon dilution depends on numbers, i.e., K_D and koff. It is widely accepted that in routine HDX-MS, the differences observed between complex and partners alone indicate binding events or their allosteric consequences, irrelevant to the complex's stability. No differences could be interpreted as possibly coming from complex dissociation, but not if we have detected changes in deuteration. We can only have "less complex" and no "more complex" or "different complex" upon dilution.

4 Ks. Trojdena Street, 02-109 Warsaw, Poland phone.: +48 22 597 07 00, fax: +48 22 597 07 15
secretariat@iimcb.gov.pl www.iimcb.gov.pl

The HD-MX data is over interpreted and poorly explained in the sections between lines 160-173. I would suggest presenting the figures in a way that helps the reader to get the most out of the data, for example using a more detailed colour code to map % D2O onto the structure. I also think the HD-MX study could be extended (as suggested above) and strengthened using additional experimental approaches to support the authors claims.

In response to the Reviewer's concerns, we have re-analyzed the raw data to generate a model with a more accurate representation of the changes occurring in the CHN-1 in the presence of UFD-2 (Fig. 2A and EV2B). However, we only discuss the effects of convincing statistical relevance.

Based on this and other suggestions from the Reviewer, we performed a series of experiments designed to confirm our model that UFD-2 stabilizes the CHN-1 U-box dimer with two E2 sites available for binding (Fig. 2B-D and EV2A, D-E).

An additional proof needs some clarification as it involves CHN-1 monomerization, on which we currently have a manuscript under revision in another journal (Balaji et al., under revision). There we show that the CHN-1 monomer is active and regulates turnover of the insulin receptor (INSR) and longevity. To prevent CHN-1 dimer formation, we mutated the conserved hydrophobic residue leucine 141 to arginine (L141R), which disrupts the dimer interface of the CC domain producing a stable monomer with the activity of a single U-box domain. We used this to determine if CHN-1 U-box dimerization is necessary for the UFD-2 dependent activity boost. We confirmed interaction between UFD-2 and CHN-1 monomer. Next, we analyzed the ubiquitylation of the CHN-1^{L141R} mutant incapable of dimerization. Indeed, the CHN-1 monomer showed no change in ubiquitylation activity in the presence of UFD-2 (though it interacts), suggesting that U-box dimerization is essential for UFD-2-dependent processivity regulation (Fig. below). Unfortunately, we cannot use these data as the manuscript on CHN-1 monomer is not yet accepted for publication but we believe this observation supports our model.

INTERNATIONAL INSTITUTE OF MOLECULAR AND CELL BIOLOGY IN WARSAW
4 Ks. Trojdena Street, 02-109 Warsaw, Poland phone.: +48 22 597 07 00, fax: +48 22 597 07 15
secretariat@iimcb.gov.pl www.iimcb.gov.pl

Figure for Referees not shown

Fig 2B: Why is the format of the data suddenly changes? In order to interpret the data, the ubiquitination also needs to be shown.

We are pleased to explain this concern - we show the entire blot below. We have previously removed this part of the blot to focus the reader's attention on the unmodified CHN-1 signal, which is the subject of quantification and accurate indicator of CHN-1 modifications. Nevertheless, we decided to perform one more experiment in the revised version and show the entire blot with quantification (Fig. 2B).

4 Ks. Trojdena Street, 02-109 Warsaw, Poland phone.: +48 22 597 07 00, fax: +48 22 597 07 15
secretariat@iimcb.gov.pl www.iimcb.gov.pl

Figure for Referees not shown

Fig 2C: The data and explanation of this figure is inadequate. The experiment is designed to detect Ub, when it would make much more sense to detect the E2 component, so that the ratio of charged and uncharged E2 could be determined. The UBE2D1Ub band assignment has not been validated by including a UBE2D1Ub plus reducing agent control. E2 discharge assays are notoriously sensitive to pH, temperature, etc and are known to discharge over time in the absence of E3, a buffer only control over the course of the experiment is therefore essential.

We thank the Reviewer for this comment. We agree that also showing E2 detection would create a complete picture. Therefore, we have performed experiments using the control - reducing agent (Fig. 2C and EV2D).

It would have added to the figure to show a titration of CHN-1 and UFD-2 with fixed E2.

We have included a new panel showing a titration of UFD-2 with fixed CHN-1 and E2 (Fig. 1B and EV2D).

I may have missed it but I can't find any reference to technical replicates in the MS methodology. I would expect to see technical triplicates for each biological replicate as standard.

In proteomics studies, I rely on collaborations at other locations for proteomic analysis, such as the Max Planck Institute for Biology of Ageing, Cologne. After discussing with their experts working daily on C. elegans proteomics, we decided on the most time-efficient yet experimentally valuable setup - five biological replicates, each with no

4 Ks. Trojdena Street, 02-109 Warsaw, Poland phone.: +48 22 597 07 00, fax: +48 22 597 07 15
secretariat@iimcb.gov.pl www.iimcb.gov.pl

technical replication. As a guideline for experimental preparation, we relied on a publications from the Kenyon, Erler, and Gartner labs, where three biological replicates (however we did five in our studies) and no technical replicates were used for similar analyses (Narayan et al., 2016; Xia et al., 2017; Ofenburger et al., 2017). Information on biological replicates is available in the Results and Materials and Methods section.

Fig 4D: The AHCY-1 portion of the immunoblot is over-exposed (especially when compared to the tubulin control); it should be redone and quantified using densitometry to determine the relative levels tubulin. However, by eye it seems fairly evident that AHCY1 levels are decreased in the chn-1 KO relative to the WT and flag controls (in relation to tubulin).

We thank the reviewer for this comment, additional set of experiments was done as suggested. To clarify, we do not see significant changes in the amount of unmodified AHCY-1 but constant changes in the signal detected with AHCY-1 antibodies over the primary signal in chn-1 and ufd-2 mutants. We observed that AHCY-1 precipitates during the preparation of worm lysates, and approximately its 60% is soluble (Fig. EV4K). Therefore, we generated a CRISPR/Cas9 knock-in GFP-based worm line to label AHCY-1 to track its localization and abundance without compromising the integrity of the worms while maintaining its natural expression level. The GFP tag did not affect AHCY-1 functionality, as its knockout is lethal to worms (WormBase and our observations). Next, we crossed chn-1(by155), ufd-2(tm1380), and CHN-1 OE worms with animals expressing GFP::AHCY-1. Microscopic analysis of GFP::AHCY-1 fluorescence levels revealed a significant signal increase in chn-1(by155) and ufd-2(tm1380) null allele worms and a decrease when CHN-1 is overexpressed (Fig. 5D). Quantitative PCR analyses showed no statistically significant changes in the AHCY-1 transcript levels (Fig. EV4J), suggesting that the increase in AHCY-1 levels is posttranslationally regulated by CHN-1 and UFD-2.

This is not unprecedented as CHIP has been identified as both a positive and negative regulator of the steady state levels of some substrates dependent on cell stress conditions. Despite not seeing a 'stabilisation' (i.e. increase in steady state levels) of the AHCY-1 protein (by immunoblot) with loss of CHN-1 function the authors still interpret the lipidomics in terms of AHCY-1 stability - presumably on the basis of the proteomics. However, caution is required during the interpretation of LC-MS/MS data as it reflects differences in the abundance of ionized peptides and not necessarily differential protein steady state levels. The abundance of ionized peptides can be influenced by a combination of factors, including post-translational modifications, inter-molecular interactions and compatibility with MS detection (ionization and fragmentation potentials).

We agree with the Reviewer that the detected protein level in LC-MS/MS can be influenced by posttranslational modification (PTM). Nevertheless, due to the low occupancy of most PTMs, the unmodified peptide abundance often remains unchanged. Additionally, for the protein AHCY-1, we have detected 50 unique peptides in total, allowing an accurate quantification by LC-MS/MS. The label-free quantification

4 Ks. Trojdena Street, 02-109 Warsaw, Poland phone.: +48 22 597 07 00, fax: +48 22 597 07 15
secretariat@iimcb.gov.pl www.iimcb.gov.pl

algorithm (MaxLFQ) uses peptide ratios-based quantification to reduce the effects of differently ionized peptides. To provide further clarification, we have added a boxplot to illustrate the Z-Score normalized raw intensities for individual AHCY-1 peptides (Fig. 5A).

The discussion is a bit limited and is treated more as a summary of the authors own findings than a forum for a more critical analysis of the data in respect to the literature. For example, human HSP70, as highlighted by the authors, inhibits CHIP on some folded substrates, however it has also been shown to stimulate the modification of some others, most convincingly the co-chaperone BAG-1. How can this be explained in light of their model? Another area that is not discussed, is the potential role here of CHN-1/CHIP-mediated ubiquitination that doesn't signal degradation - this is very pertinent as the current study does not show up regulation of AHCY-1 steady state levels in KO worms. Finally, as pointed out by the author a large portion of the literature around CHN-1/CHIP is focused on proteostasis and its role as a co-chaperone for HSP70/HSP-1. The manuscript is crying out for a critical evaluation of how the model presented here would inform the current view of CHN-1/CHIP function.

We thank the Reviewer for pointing out the need for more critical discussion. We have reformated the discussion in the revised manuscript. The following is an excerpt from the BAG-1-related part of discussion.
" We observed that worm DAF-21/HSP90 has a lower affinity for CHN-1 and does not affect CHN-1 activity, unlike HSP-1/HSP70. Consistent with this observation, the C-terminal HSP70 peptide blocks CHIP activity markedly greater than the HSP90 peptide, which binds to the CHIP TPR domain weaker compared with the HSP70 peptide (Narayan et al., 2015). The K30A missense mutation in CHIP, which likely mimics HSP70 binding, also reduces CHIP activity. Furthermore, HSP70 inhibits CHIP-dependent ubiquitination of folded substrates like Smad1/5 (Wang et al., 2011), PPARγ2 (Kim et al., 2017), p53, or IRF-1 (Narayan et al., 2015). It is noteworthy that HSP70 can stimulate BAG-1 cochaperone ubiquitination under experimental conditions that inhibit p53 and IRF-1 modification; however, the increase in BAG-1 modification was not accompanied by an increase in CHIP auto-Ub, an indicator of its activity. Moreover, stimulation of BAG-1 ubiquitination was suppressed by the C-terminal peptide of HSP70 (^{634}GPTIEEVD^{641}). Thus, the authors suggest that HSP70, through its direct interaction with BAG-1, may facilitate its modification by CHIP. HSP70 can also exert different effects on CHIP-dependent ubiquitylation of TP63 isoforms (a homolog of the p53 tumor suppressor), i.e., it potentiates ubiquitination of the TAp63 isoform and reduces modification of the ΔNp63 isoform (Wu et al., 2021). However, the authors did not present results on CHIP auto-Ub or on the competition between TP63 isoforms and HSP70 for CHIP binding, which would allow a precise determination of the effect of HSP70 on CHIP processivity in their experimental system. Two different heat shock cognate protein 70 (HSC70, a member of the heat shock protein 70 family) cochaperones, BAG-2 and HspBP1, limit CHIP activity (Alberti et al., 2004; Arndt et al., 2005; Dai et al., 2005). BAG-2 inhibition is associated with reduced E2 accessibility, which is likely related to a shift favoring the CHIP "closed"

4 Ks. Trojdena Street, 02-109 Warsaw, Poland phone.: +48 22 597 07 00, fax: +48 22 597 07 15
secretariat@iimcb.gov.pl www.iimcb.gov.pl

state and stimulation of chaperone-assisted CFTR maturation. Moreover, CHIP can enhance the ubiquitination of Pael-R (Parkin-associated endothelin receptor-like receptor) by Parkin E3 ligase, and this modification was inhibited by HSP70 (Imai et al., 2002). The C. elegans Parkin ortholog, PDR-1, also interacts with CHN-1, and both are expressed in neurons and body wall muscles. However, their mechanism of action and the substrates modified by the CHN-1–PDR-1 complex are obscure. The above examples in concert with our data indicate an evolutionarily conserved role for HSP70/HSP-1 as a negative regulator of CHIP/CHN-1. We cannot exclude the possibility that posttranslational modifications of CHN-1 or the presence of specific factors that would limit the interaction of HSP-1 with the U-box domains could sustain the ability of CHN-1 to ubiquitinate chaperone-bound substrates".

Other comments:

The writing of the manuscript feels rushed with the methodology and data not being fully explained - there are several instances where data is not referred to or where the methodology is poorly linked to the data.

Done as suggested. We rewrote the manuscript and submitted it to a professional editor before submission.

The text states that assays with worms plus MG132 and NEM are shown but I do not see the data?

This information is now included in the Fig. 4B and Fig. 5C.

Fig EV1C: It appears that the exposure time in figure EV1C is a lot shorter than in other figures (compare for example the unmodified band in Fig EV1F) to which it is being compared. Never the less it is possible to discern bands that are present in the plus UFD-2 lane compared to the minus UFD-2 lane. There should be a positive control present on the immunoblot that allows comparison with other gels, and a comment should be made about these bands.

We agree with the Reviewer and have conducted a new experiment with the required controls. We ruled out the possibility that it was UFD-2 that modified CHN-1 because it was unable to ubiquitylate inactive CHN-1^{H218Q}, which probably lost its affinity towards its cognate E2 (Tawo et al., 2017) (Fig. EV1F). However, we noted that CHN-1^{H218Q} was modified specifically in the presence of UFD-2^{P951A} (bands marked with an asterisk), which might suggest recovery of some minimal activity, reflecting possible structural changes in the CHN-1 U-box domain during an interaction with UFD-2.

There is no plus UFD-2 minus CHN-1 control in figure EV1B.

We have included a blot with the requested control (now Fig. EV1D).

4 Ks. Trojdena Street, 02-109 Warsaw, Poland phone.: +48 22 597 07 00, fax: +48 22 597 07 15
secretariat@iimcb.gov.pl www.iimcb.gov.pl

The authors should comment on the fact that addition of UFD-2 to DTPR-CHN-1 actually decreases the amount of Ub detected.

We have not been able to determine the cause of the slight reduction in the signal. Based on the high activity data of UFD-2 itself (Fig. EV1B) and other observations, we can speculate that UFD-2 utilized most of the Ub for the auto-Ub reaction, thereby reducing its availability to the slow-operating CHN-1 mutant. We added this information to the manuscript.

S3F (line 242 and 245) should be EV3F, there are several other places where EV is replaced with S.

We thank the Reviewer for pointing this out. We have unified all references to figures in the revised manuscript.

Fig EV4G: A minus E3 control needed.

We thank the Reviewer for bringing this detail to our attention. We did a blot with the required control (Fig. below). Because of our mistake, we did not use it in the figure. In the revised manuscript it is Fig. EV4I.

Figure for Referees not shown

Fig 4C: K48 and K63 are used in the assay but the data do not appear to be referred to in the text and should therefore be removed from the figure.

We thank the Reviewer for pointing this out. We used the wrong blot to make a panel. We show ubiquitylation changes of AHCY-1 using different Ub variants, but these experiments were our internal controls for monitoring E2 activity from R&D Systems

4 Ks. Trojdena Street, 02-109 Warsaw, Poland phone.: +48 22 597 07 00, fax: +48 22 597 07 15
secretariat@iimcb.gov.pl www.iimcb.gov.pl

(not all batches are of adequate quality). The revised manuscript shows the experiment without adding ubiquitin variants (Fig. 5B).

Referee #2

Major concerns:

It is very interesting that UFD-2 stabilizes CHN-1 U-box dimer, whereas the authors only show the HDX-MS results. Size exclusion chromatography profiles of CHN-1 alone, CHN-1, and UFD-2 (or UFD-2 PEPTIDE2) may be helpful.

We agree with the Reviewer and have included the requested chromatogram (Fig. EV2A). We observed a tendency of CHN-1 to form oligomers, which can be seen in the size-exclusion chromatography (SEC) chromatogram as a prominent peak corresponding to its oligomeric distribution followed by a peak corresponding to the CHN-1 dimer (for each in vitro experiment, we worked only with the CHN-1 dimer fraction). When we mixed CHN-1 and UFD-2 in equal molar ratios and performed SEC separation, we obtained peaks corresponding to the respective proteins without the CHN-1 oligomerization signal, suggesting a shift toward CHN-1 dimer stabilization by UFD-2 (Fig. EV2A). Unfortunately, we did not detect a stable CHN-1–UFD-2 complex upon SEC separation, highlighting the dynamic and transient nature of this interaction. To gain mechanistic insight into the role of UFD-2 binding to CHN-1, we performed the previously included hydrogen-deuterium exchange mass spectrometry (HDX-MS) of the dimerization process of both CHN-1 alone and CHN-1 in the presence of UFD-2.

In addition, they have mapped the binding regions in UFD-2 EEYD, whether the mutation in this region (UFD-2 EEYD mutations) could abolish the function of UFD-2 in CHN-1 U-box dimer stabilization and ubiquitylation activity?

We thank the Reviewer for the suggestion. We purified a recombinant UFD-2^{EEVD} mutant protein and performed in vitro ubiquitylation assays. Indeed, UFD-2^{EEVD} no longer stimulated CHN-1 activity and even reduced it along with AHCY-1 ubiquitination. We have prepared new panels concerning the impact of mutant UFD-2^{EEVD} on CHN-1 processivity (Fig. 3E, 5B and EV4G).

The authors claim that UFD-2 stabilizes the U-box dimer with two E2 sites, whereas their results could not sufficiently support this major point, more evidences are definitely needed to support this conclusion. First, they showed UBE2D1GST-Ub (while they have not described how to produce ubiquitin-charged GST-UBE2D1 in the methods or legends) could directly bind to CHN-1, however, UFD-2(P951A) could not enhance the amount of CHN-1 in the immunoprecipitate in Figure EV1E. They should further confirm that CHN-1/UFD-2 could bind with two E2.

4 Ks. Trojdena Street, 02-109 Warsaw, Poland phone.: +48 22 597 07 00, fax: +48 22 597 07 15
secretariat@iimcb.gov.pl www.iimcb.gov.pl

We thank the Reviewer for raising this issue. Based on IP experiments, it is difficult to readily interpret the amount of E2 bound by CHN-1 because IP is a non-equilibrium system. Thus, we reformed some figure panels and performed a set of experiments designed to confirm our model that UFD-2 stabilizes the CHN-1 U-box dimer with two E2 sites available for binding (Fig. 2B-D and EV2D-E). First, we have re-analyzed the raw data of HDX-MS to generate a model with a more accurate representation of the changes occurring in the CHN-1 in the presence of UFD-2 (Fig. 2A and EV2B). Our model suggests that in a complex with non-active UFD-2^{P951A}, stabilization of the U-box dimer of CHN-1 would still occur. To verify this, we performed another auto-Ub assay with the Ube2W conjugating enzyme, which is known to maintain a strict 1:1 stoichiometry with substrate (Tatham et al., 2013; Vittal et al., 2013, Christensen et.al., 2007) and to catalyze mono-Ub of CHIP (Scaglione et al., 2011). Considering these characteristics, we added CHN-1 or CHN-1/UFD-2^{P951A} to charged Ube2W-Ub and Ube2W-Ub^{FLAG} and followed the ubiquitylation profile. When both E2 species are included in the reaction, we should observe mono-Ub of CHN-1 by Ub and Ub^{FLAG} (due to the FLAG tag, Ub molecules migrate slower on SDS-PAGE, allowing differentiation of mono-Ub from mono-Ub^{FLAG} on a single immunoblot) as well as a 2-fold increase in the level of CHN-1 ubiquitylation for the complex with UFD-2^{P951A} compared with that of CHN-1 alone. Indeed, we noted the predicted increase in CHN-1 mono-Ub with the two Ub variants (Fig. 2D). By contrast, the presence of HSP-1 in the reaction reduced the Ub conjugation to CHN-1 and altered the ratio of Ub to Ub^{FLAG} (Fig. EV2E). This increase in the stoichiometric ratio in the presence of UFD-2^{P951A} suggests the existence of dimeric CHN-1 with two available U-box domains, while HSP-1 might promote a CHN-1 conformation that limits E2 access to the U-box domains. Charged UBE2D1 was obtained from R&D Systems or generated by us (UBE2W-Ub and UBE2W-Ub^{FLAG}). We placed an appropriate description in the Materials and Methods section.

An additional piece of evidence needs some clarification as it involves CHN-1 monomerization, on which we currently have a manuscript under revision in another journal (Balaji et al., under revision. There we show that the CHN-1 monomer is active and regulates turnover of the insulin receptor (INSR) and longevity. To prevent CHN-1 dimer formation, we mutated the conserved hydrophobic residue leucine 141 to arginine (L141R), which disrupts the dimer interface of the CC domain producing a stable monomer with the activity of a single U-box domain. We used this to determine if CHN-1 U-box dimerization is necessary for the UFD-2 dependent activity boost. We conformed interaction between UFD-2 and CHN-1 monomer. Next, we analyzed the ubiquitylation of the CHN-1^{L141R} mutant incapable of dimerization. Indeed, the CHN-1 monomer showed no change in ubiquitination activity in the presence of UFD-2 (though it interacts), suggesting that U-box dimerization is essential for UFD-2 dependent

4 Ks. Trojdena Street, 02-109 Warsaw, Poland phone.: +48 22 597 07 00, fax: +48 22 597 07 15
secretariat@iimcb.gov.pl www.iimcb.gov.pl

processivity regulation (Fig. below). Unfortunately, we cannot use these data as the manuscript on CHN-1 monomer is not yet accepted for publication but we believe this observation supports our model.

Figure for Referees not shown

4 Ks. Trojdena Street, 02-109 Warsaw, Poland
secretariat@iimcb.gov.pl
phone.: +48 22 597 07 00, fax: +48 22 597 07 15
www.iimcb.gov.pl

Second, authors showed UFD-2 (P951A) could dramatically enhance the autoubiquitylation of CHN-1 by using equal amount of UBE2D1 in Figure 2B, whereas the effective control groups are loss. The half and 2-fold of UBE2D1 in Figure 2B lane 6 should be used in the CHN-1/UFD-2 ubiquitination assay.

We agree with the Reviewer, and we we titrated UBE2D1 (0.5–4 µM) at a fixed concentration of CHN-1 (1.3 µM) or CHN-1 complexed with UFD-2^{P951A}, which cannot interact with UBE2D1, and conducted an auto-Ub assay. We observed that at a constant Ub concentration, increasing the E2 concentration led to increased CHN-1 activity. However, even at the highest E2 concentration (4 µM), CHN-1 processivity did not reach the same level as in the presence of inactive UFD-2^{P951A} and approximately 10-fold lower E2 concentration (0.5 µM) (Fig. 2B). Thus, the increased CHN-1 activity of the CHN-1–UFD-2 complex was not due to an increased local E2 concentration but rather to the enhanced processivity of the E2 enzyme bound to the CHN-1 U-box. To verify this hypothesis, we performed an E2-discharging assay in the presence of CHN-1 alone or after mixing with UFD-2^{P951A} to track the use of charged-E2 by CHN-1 only. We observed that in the presence of UFD-2^{P951A}, CHN-1 could discharge at least twice as much UBE2D1-Ub compared to CHN-1 alone, independent of the E2 concentration used, which was revealed by the accumulation of uncharged UBE2D1 (Fig. 2C and EV2D).

Third, the E2-discharging assay is problematic. The discharging reaction samples should also be treated with reducing agent and detected with UBE2D1 blotting to further confirm the UBE2D1-Ub bands. In addition, the auto ubiquitylated CHN-1 and Di-Ub should be observed to reflect the activity of CHN-1.

We agree that showing E2 detection would create a complete picture. Therefore, we have redone experiments also using the control (reducing agent) suggested by the Reviewer (Fig. 2C and EV2D).

As there are some reported U-box:E2 structure (PMID: 20696396, 18485199, 20146531, 16307917), the authors could dock them with the stabilized CHN-1 dimer to further support their statements.

We are pleaed to clarify this concern. The model presented was not docked because the PDB 2OXQ co-crystal already contains the same E2 protein, and the zebrafish homolog CHN-1 shows 86% sequence similarity - a docked model of these proteins would achieve lower resolution than the complex in the crystal. We prepared the appropriate model and panel (Fig. EV2C) which underlines that the corresponding residues are conserved at the binding interface.

4 Ks. Trojdena Street, 02-109 Warsaw, Poland phone.: +48 22 597 07 00, fax: +48 22 597 07 15
secretariat@iimcb.gov.pl www.iimcb.gov.pl

Figure for Referees not shown

Given that HSP-1 could diminish the activity of CHN-1/UFD-2 and HSP-1 shows a higher binding affinity with CHN-1 than UFD-2, whether HSP-1 could compete with UFD-2 for binding CHN-1?

We tested whether the chaperones could compete with UFD-2 for CHN-1 binding. We performed an ELISA-based titration assay to determine the dissociation of CHN-1 from immobilized UFD-2 induced by the presence of HSP-1 or DAF-21. As the concentration of chaperones increased, the CHN-1 signal decreased (increased dissociation from the complex with UFD-2), indicating that chaperones compete with UFD-2 for the CHN-1 TPR domain (Fig. EV3A-C).

AHCY-1 is a newly identified substrate for CHN-1/UFD-2, whereas the protein level of AHCY-1 is not consistent in MS data and Western blot in Figure 4A and 4D. As the authors claim that assembly with UFD-2 enables CHIP/CHN-1 to regulate lipid metabolism by ubiquitylation of AHCY-1, the UFD-2 EEYD mutations should be provide as negative control in both AHCY-1 ubiquitination assay and the lipid examination.

We thank the Reviewer for raising this issue. We further developed a section related to the ubiquitylation and stability of AHCY-1 (Fig. 5B-D and EV4H-J). Since we did not observe a change in the stability of unmodified AHCY-1 in worm lysates, which could be related to the tendency of AHCY-1 to precipitate during sample preparation (Fig. EV4K), we generated a CRISPR/Cas9 knock-in GFP-based worm line to label AHCY-1 to track its localization and abundance without compromising the integrity of the worms while maintaining its natural expression level. The GFP tag did not affect AHCY-1 functionality, as its knockout is lethal to worms (WormBase and our observations). Next, we crossed chn-1(by155), ufd-2(tm1380), and CHN-1 OE worms with animals expressing GFP::AHCY-1. Microscopic analysis of GFP::AHCY-1 fluorescence levels revealed a significant signal increase in chn-1(by155) and ufd-2(tm1380) null allele worms and a decrease when CHN-1 is overexpressed (Fig. 5D). Quantitative PCR

4 Ks. Trojdena Street, 02-109 Warsaw, Poland phone.: +48 22 597 07 00, fax: +48 22 597 07 15
secretariat@iimcb.gov.pl www.iimcb.gov.pl

analyses showed no statistically significant changes in the AHCY-1 transcript levels (Fig. EV4J), suggesting that the increase in AHCY-1 levels is post-translationally regulated by CHN-1 and UFD-2.

UFD-2^{EEVD} no longer stimulated CHN-1 activity and even reduced it along with AHCY-1 ubiquitylation. We have prepared new panels concerning the impact of mutant UFD-2^{EEVD} on CHN-1 processivity (Fig. 3E, 5B and EV4G). In addition, we generated worms expressing the UFD-2^{EEVD} mutant via CRISPR/Cas9 editing. Surprisingly, unlike null allele ufd-2(tm1380) or worms producing UFD-2^{P951A}, UFD-2^{EEVD} animals exhibited growth and developmental defects. Worms normally go through four larval stages to reach adulthood. We observed that UFD-2^{EEVD} mutants are smaller and develop significantly slower before reaching young adulthood (Fig. below). We, therefore, decided not to use these worms for lipidomic analyses, being concerned about the significant abnormalities in lipid composition associated with the above phenotypes. Nonetheless, we find this result interesting, indicating the importance of the UFD-2 EEYD sequence. We plan to investigate why worms exhibit this phenotype in the future studies and whether this is due to deregulation of CHN-1, AHCY-1, or unknown UFD-2 substrate(s).

Figure for Referees not shown

Minor comments:
Figure 1A, why the authors do not used the E2 of CHN-1 in C. elegans (UBC-2/Let-70, PMID: 15294159)?

Done as suggested. We prepared the recombinant E2 enzyme LET-70 and performed the expected analysis of CHN-1 activity. The presence of UFD-2 in the reaction also potentiated CHN-1 auto-Ub with LET-70, the C. elegans ortholog of UBE2D proteins (Fig. EV1C).

4 Ks. Trojdena Street, 02-109 Warsaw, Poland phone.: +48 22 597 07 00, fax: +48 22 597 07 15
secretariat@iimcb.gov.pl www.iimcb.gov.pl

Figure 1B, time-dependent auto-Ub experiment sounds not work, the authors should shorten time.

We agree with the Reviewer and prepared new figure panels with optimized time-course experiments (Fig. 1A, EV1G-H) and added quantification along with statistics.

Figure EV1C, CHN-1 signal could be observed in lane without CHN-1?

We apologize, but this was an incorrect description of the figure (there was no lane without CHN-1). Nevertheless, we have conducted a new experiment with the required controls. We confirmed that UFD-2 could not ubiquitylate the inactive CHN-1^{H218Q} mutant, which likely lost its affinity for the E2 cognate (Tawo et al., 2017) (Fig. EV1F). However, we noticed, especially in the presence of UFD-2^{P951A}, that there was a modification of the CHN-1^{H218Q} mutant (bands marked with an asterisk), suggesting that the CHN-1 mutant regained some activity and thus structural changes in the U-box domain during interaction with UFD-2.

Figure 2B, C, as the authors state that immunoblots representative of n = 3 experiments are shown, statistical analysis could be performed. Same things in Figure 3B, 3C and 4C.

We thank the Reviewer for pointing this out. We have added quantification along with statistics where necessary (Fig. 1A-B, 2B-D, 3B-C, 4A-B, 5B-C and EV1K).

It is very interesting that a single point mutation in HSP-1 (EEVD to EEYD) show an opposite effect on CHN-1, whether the similar mutation in UFD-2 also show an opposite effect?

This is an excellent point; indeed, UFD-2^{EEVD} no longer stimulated CHN-1 activity and even reduced it along with AHCY-1 ubiquitination. We have prepared new panels concerning the impact of mutant UFD-2^{EEVD} on CHN-1 processivity (Fig. 3E, 5B and EV4G).

Figure 4C, 4 may not indicate the AHCY-1 di-Ub, whose MW should be ~70kD.

We are pleased to clarify this concern. The migration of ubiquitylated proteins does not always correlate with the calculation of their mass-dependent distribution on the gel. To determine whether these signals are di-Ub AHCY-1, we performed an AHCY-1 ubiquitylation reaction using an Ub mutant (Ub-NoK) that cannot form Ub chains (only monoubiquitylation possible) (Fig. 5B).

L463 worms treated with a proteasome inhibitor (MG132, 10µM) or DUB inhibitor (NEM, 100mM). Which lane was treated with MG132? And which one was NEM treated?

4 Ks. Trojdena Street, 02-109 Warsaw, Poland phone.: +48 22 597 07 00, fax: +48 22 597 07 15
secretariat@iimcb.gov.pl www.iimcb.gov.pl

We thank the Reviewer for raising this issue, this information is now included in the Fig. 4B and Fig. 5C.

The ubiquitinated AHCY-1 in Figure 4D should be confirmed by AHCY-1 immunoprecipitation with ubiquitin blotting. And the overexpression of UFD-2, UFD-2 EEYD mutations in Figure 4D is helpful for further confirm AHCY-1 ubiquitination depends on the UFD-2 facilitated CHN-1 dimerization.

We further developed a section related to the ubiquitination and stability of AHCY-1 (Fig. 5B-D and EV4H-J). Since we did not show a change in the level of unmodified AHCY-1 in worm lysates, which may be related to the tendency of AHCY-1 to precipitate during sample preparation (Fig. EV4K), we generated a worm line based on CRISPR/Cas9 knock-in of GFP to label AHCY-1 to track localization and abundance without compromising the integrity of the worms, while maintaining the natural expression level. Microscopic analysis of GFP::AHCY-1 fluorescence levels showed a significant increase in chn-1(by155) and ufd-2(tm1380) null alleles and a decrease associated with CHN-1 overexpression (Fig. 5D). Quantitative PCR analysis showed no statistically significant changes in AHCY-1 transcript levels in response to knock out of chn-1 or ufd-2 (Fig. EV4J), suggesting that CHN-1 and UFD-2 posttranslationally regulate the increase in AHCY-1 levels.

Next, we generated worms expressing the UFD-2^{EEVD} (EEYD to EEVD change) mutant via CRISPR/Cas9 editing. Surprisingly, unlike null allele ufd-2(tm1380) or worms producing UFD-2^{P951A}, UFD-2^{EEVD} animals exhibited growth and developmental defects. Worms normally go through four larval stages to reach adulthood. We observed that UFD-2^{EEVD} mutants are smaller and develop significantly slower before reaching young adulthood (Fig. on page 18 of this letter). We, therefore, decided not to use these worms in subsequent experiments. By performing AHCY-1 ubiquitylation reactions in vitro, we noted that in the presence of UFD-2^{EEVD} mutant CHN-1 carried out modifications less efficiently, indicating the specificity of the EEYD sequence of UFD-2 in regulating its activity (Fig. 5B).

Unfortunately, generation of worms overexpressing UFD-2 did not work out for us, therefore we used SunyBiotech service. The idea was to generate animals expressing multiple copies of UFD-2 under its regulatory sequences (promoter and 3'UTR). Since we do not know what sequence constitutes the promoter of ufd-2, we assumed that 2k bp downstream of the start codon should be enough, and 500 bp upstream of the stop codon would contain 3'UTR. SunyBiotech also had problems generating a homozygous line - we placed the order in October 2021 and received the PHX996 worms of genotype unc-119(ed9); Is[Pufd-2-ufd-2-ufd-2 3'UTR] in late March 2022. Unfortunately, a line validation that compared the amount of UFD-2 protein in wild-type worms with those potentially overexpressing UFD-2 (UFD-2 OE) showed no differences (figure below), suggesting no overexpression occurs even though the transgene was integrated (verification by genotyping). Perhaps the choice of UFD-2 regulatory elements was inappropriate, or there is a silencing of transgene expression in subsequent generations.

4 Ks. Trojdena Street, 02-109 Warsaw, Poland phone.: +48 22 597 07 00, fax: +48 22 597 07 15
secretariat@iimcb.gov.pl www.iimcb.gov.pl

Figure for Referees not shown

The unmodified form of AHCY-1 is dominant compared to the ubiquitin-modified fraction of AHCY-1, which we also observed in an in vitro assay (Fig. 5B). Thus, only 10-15% of the protein is ubiquitylated by the CHN-1–UFD-2 complex. Unfortunately, our in-house antibody was poorly suited for AHCY-1 immunoprecipitation (we wanted to verify this material for ubiquitylation later using anti-Ub detection). Therefore, we were unable to perform the analyses requested by the Reviewer. However, immunoblotting with anti-AHCY-1 antibodies detected higher molecular weight smeared bands, likely corresponding to poly-Ub AHCY-1 species (Fig. 5C) as these are only visible after treatment of the worms with inhibitors of the proteasome (MG-132) and deubiquitinating enzyme (DUB) (N-methylmaleimide, NEM) These AHCY-1 modifications were more abundant when chn-1 was overexpressed and were reduced in chn-1(by155) and ufd-2(tm1380) mutant worms compared with the ACHY-1 status in wild-type animals (Fig. 5C).

On behalf of all co-authors, I once again thank the Editor and the Reviewers for their invaluable feedback and all the suggestions on our manuscript.

With best regards,

Wojciech Pokrzywa

4 Ks. Trojdena Street, 02-109 Warsaw, Poland phone.: +48 22 597 07 00, fax: +48 22 597 07 15
secretariat@iimcb.gov.pl www.iimcb.gov.pl

Thank you again for submitting your revised manuscript to The EMBO Journal. It has now been re-reviewed by the two original referees, and I am happy to inform you that both of them are satisfied with the responses and revisions and have no further concerns. We shall therefore be ready to proceed with acceptance and EMBO Journal publication, as soon as a few remaining editorial points listed below have been addressed:

REFEREE REPORTS

Referee #1:

The revised manuscript is greatly improved and the discussion is more holistic. The authors are applauded for taking the comments of the reviewers seriously and committing to address them as fully as possible. Although additional HX-MS would be informative the cost limitations are appreciated by the reviewer. The reviewer finds the shared (unpublished) data on monomeric CHIP to be of great interest and agrees that the findings - when published - will support the current manuscript. Finally, the additional experiments on AHCY-1 make the worm data more competing although the structure:function data are now strong enough to stand alone.

Referee #2:

I find that the revised manuscript has improved significantly. The authors have now successfully addressed my concerns.

The authors have made all requested editorial changes.

Thank you for submitting your final revised manuscript for our consideration. I am pleased to inform you that we have now accepted it for publication in The EMBO Journal.

EMBO Press Author Checklist

Corresponding Author Name: Wojciech Pokrzywa
Journal Submitted to: EMBO Journal
Manuscript Number: EMBOJ-2021-109566

USEFUL LINKS FOR COMPLETING THIS FORM
The EMBO Journal - Author Guidelines
EMBO Reports - Author Guidelines
Molecular Systems Biology - Author Guidelines
EMBO Molecular Medicine - Author Guidelines

Reporting Checklist for Life Science Articles (updated January 2022)
This checklist is adapted from Materials Design Analysis Reporting (MDAR) Checklist for Authors. MDAR establishes a minimum set of requirements in transparent reporting in the life sciences (see Statement of Task: 10.31222/osf.io/9sm4x). Please follow the journal's guidelines in preparing your manuscript.
Please note that a copy of this checklist will be published alongside your article.

Abridged guidelines for figures
1. Data
The data shown in figures should satisfy the following conditions:
- the data were obtained and processed according to the field's best practice and are presented to reflect the results of the experiments in an accurate and unbiased manner.
- ideally, figure panels should include only measurements that are directly comparable to each other and obtained with the same assay.
- plots include clearly labeled error bars for independent experiments and sample sizes. Unless justified, error bars should not be shown for technical replicates.
- if n<5, the individual data points from each experiment should be plotted. Any statistical test employed should be justified.
- Source Data should be included to report the data underlying figures according to the guidelines set out in the authorship guidelines on Data Presentation.

2. Captions
Each figure caption should contain the following information, for each panel where they are relevant:
- a specification of the experimental system investigated (eg cell line, species name).
- the assay(s) and method(s) used to carry out the reported observations and measurements.
- an explicit mention of the biological and chemical entity(ies) that are being measured.
- an explicit mention of the biological and chemical entity(ies) that are altered/varied/perturbed in a controlled manner.
- the exact sample size (n) for each experimental group/condition, given as a number, not a range;
- a description of the sample collection allowing the reader to understand whether the samples represent technical or biological replicates (including how many animals, litters, cultures, etc.).
- a statement of how many times the experiment shown was independently replicated in the laboratory.
- definitions of statistical methods and measures:
  - common tests, such as t-test (please specify whether paired vs. unpaired), simple χ2 tests, Wilcoxon and Mann-Whitney tests, can be unambiguously identified by name only, but more complex techniques should be described in the methods section;
  - are tests one-sided or two-sided?
  - are there adjustments for multiple comparisons?
  - exact statistical test results, e.g., P values = x but not P values < x;
  - definition of 'center values' as median or average;
  - definition of error bars as s.d. or s.e.m.

Please complete ALL of the questions below.
Select "Not Applicable" only when the requested information is not relevant for your study.

Materials

Newly Created Materials	Information included in the manuscript?	In which section is the information available? (Reagents and Tools Table, Materials and Methods, Figures, Data Availability Section)
New materials and reagents need to be available; do any restrictions apply?	Select response	Data and Materials Availability

Antibodies	Information included in the manuscript?	In which section is the information available? (Reagents and Tools Table, Materials and Methods, Figures, Data Availability Section)
For **antibodies** provide the following information:<br>- Commercial antibodies: RRID (if possible) or supplier name, catalogue number and or/clone number<br>- Non-commercial: RRID or citation	Select response	Reagents and Tools Table

DNA and RNA sequences	Information included in the manuscript?	In which section is the information available? (Reagents and Tools Table, Materials and Methods, Figures, Data Availability Section)
**Short novel DNA or RNA including primers, probes**: provide the sequences.	Select response	List of constructs and oligonucleotides

Cell materials	Information included in the manuscript?	In which section is the information available? (Reagents and Tools Table, Materials and Methods, Figures, Data Availability Section)
**Cell lines:** Provide species information, strain. Provide accession number in repository **OR** supplier name, catalog number, clone number, and/**OR** RRID.	Select response	NA
**Primary cultures:** Provide species, strain, sex of origin, genetic modification status.	Select response	NA
Report if the cell lines were recently **authenticated** (e.g., by STR profiling) and tested for mycoplasma contamination.	Select response	NA

Experimental animals	Information included in the manuscript?	In which section is the information available? (Reagents and Tools Table, Materials and Methods, Figures, Data Availability Section)
**Laboratory animals or Model organisms:** Provide species, strain, sex, age, genetic modification status. Provide accession number in repository **OR** supplier name, catalog number, clone number, **OR** RRID.	Select response	Materials and Methods
**Animal observed in or captured from the field:** Provide species, sex, and age where possible.	Select response	Materials and Methods
Please detail **housing and husbandry conditions**.	Select response	Materials and Methods

Plants and microbes	Information included in the manuscript?	In which section is the information available? (Reagents and Tools Table, Materials and Methods, Figures, Data Availability Section)
**Plants:** provide species and strain, ecotype and cultivar where relevant, unique accession number if available, and source (including location for collected wild specimens).	Select response	NA
**Microbes:** provide species and strain, unique accession number if available, and source.	Select response	Reagents and Tools Table

Human research participants	Information included in the manuscript?	In which section is the information available? (Reagents and Tools Table, Materials and Methods, Figures, Data Availability Section)
If collected and within the bounds of privacy constraints report on age, sex and gender or ethnicity for all study participants.	Select response	NA

Core facilities	Information included in the manuscript?	In which section is the information available? (Reagents and Tools Table, Materials and Methods, Figures, Data Availability Section)
If your work benefited from core facilities, was their service mentioned in the acknowledgments section?	Select response	Acknowledgments

Design

Study protocol	Information included in the manuscript?	In which section is the information available?<br>(Reagents and Tools Table, Materials and Methods, Figures, Data Availability Section)
If study protocol has been **pre-registered, provide DOI in the manuscript**. For clinical trials, provide the trial registration number **OR** cite DOI.	Select response	NA
Report the **clinical trial registration number** (at ClinicalTrials.gov or equivalent), where applicable.	Select response	NA

Laboratory protocol	Information included in the manuscript?	In which section is the information available?<br>(Reagents and Tools Table, Materials and Methods, Figures, Data Availability Section)
Provide DOI OR other citation details if **external detailed step-by-step protocols** are available.	Select response	NA

Experimental study design and statistics	Information included in the manuscript?	In which section is the information available?<br>(Reagents and Tools Table, Materials and Methods, Figures, Data Availability Section)
Include a statement about **sample size** estimate even if no statistical methods were used.	Select response	Sample size determination was done according to standard C. elegans approaches. Exact sample sizes are indicated in the corresponding figure legends and Supplementary Information. All experimental findings were
Were any steps taken to minimize the effects of subjective bias when allocating animals/samples to treatment (e.g. **randomization procedure**)? If yes, have they been described?	Select response	Young hermaphrodites were randomly picked from our maintenance plates. After egg-laying, larvae were raised until adulthood, and adult worms were randomly assigned to the different treatment conditions (e.g., temperature, RNAi treatment) for the motility assays. The different conditions were
Include a statement about **blinding** even if no blinding was done.	Select response	The investigators were blinded to group allocation during data collection for behavioral analysis data collection. For the rest of the experiments blinding was not performed due to the unambiguous nature of measurements and
Describe **inclusion/exclusion criteria** if samples or animals were excluded from the analysis. Were the criteria pre-established?<br><br>If sample or data points were omitted from analysis, report if this was due to attrition or intentional exclusion and provide justification.	Select response	No data was excluded from the analysis.
For every figure, are **statistical tests** justified as appropriate? Do the data meet the assumptions of the tests (e.g., normal distribution)? Describe any methods used to assess it. Is there an estimate of variation within each group of data? Is the variance similar between the groups that are being statistically compared?	Select response	Yes. Description of used statistical test is in the Figure legends

Sample definition and in-laboratory replication	Information included in the manuscript?	In which section is the information available?<br>(Reagents and Tools Table, Materials and Methods, Figures, Data Availability Section)
In the figure legends: state number of times the experiment was **replicated** in laboratory.	Select response	Figure legends
In the figure legends: define whether data describe **technical or biological replicates**.	Select response	Figure legends

Ethics

Ethics	Information included in the manuscript?	In which section is the information available?<br>(Reagents and Tools Table, Materials and Methods, Figures, Data Availability Section)
Studies involving **human participants**: State details of **authority granting ethics approval** (IRB or equivalent committee(s), provide reference number for approval.	Select response	NA
Studies involving **human participants**: Include a statement confirming that **informed consent** was obtained from all subjects and that the experiments conformed to the principles set out in the WMA Declaration of Helsinki and the Department of Health and Human Services Belmont Report.	Select response	NA
Studies involving **human participants:** For publication of **patient photos**, include a statement confirming that consent to publish was obtained.	Select response	NA
Studies involving experimental **animals**: State details of **authority granting ethics approval** (IRB or equivalent committee(s), provide reference number for approval. Include a statement of compliance with ethical regulations.	Select response	The project included research using GMO (Caenorhabditis elegans) and non-pathogenic Escherichia coli strains, for which we have a permit (Decision of the Minister of Environment No. 128/2017, valid indefinitely).
Studies involving **specimen and field samples:** State if relevant **permits** obtained, provide details of authority approving study; if none were required, explain why.	Select response	NA

Dual Use Research of Concern (DURC)	Information included in the manuscript?	In which section is the information available?<br>(Reagents and Tools Table, Materials and Methods, Figures, Data Availability Section)
Could your study fall under dual use research restrictions? Please check biosecurity documents and list of **select agents and toxins** (CDC): https://www.selectagents.gov/sat/list.htm	Select response	NA
If you used a select agent, is the security level of the lab appropriate and reported in the manuscript?	Select response	NA
If a study is subject to dual use research of concern regulations, is the name of the **authority granting approval and reference number** for the regulatory approval provided in the manuscript?	Select response	NA

Reporting

The MDAR framework recommends adoption of discipline-specific guidelines, established and endorsed through community initiatives. Journals have their own policy about requiring specific guidelines and recommendations to complement MDAR.

Adherence to community standards	Information included in the manuscript?	In which section is the information available?<br>(Reagents and Tools Table, Materials and Methods, Figures, Data Availability Section)
State if relevant guidelines or checklists (e.g., **ICMJE, MIBBI, ARRIVE, PRISMA**) have been followed or provided.	Select response	NA
For **tumor marker prognostic studies**, we recommend that you follow the **REMARK** reporting guidelines (see link list at top right). See author guidelines, under 'Reporting Guidelines'. Please confirm you have followed these guidelines.	Select response	NA
For **phase II and III randomized controlled trials**, please refer to the **CONSORT** flow diagram (see link list at top right) and submit the CONSORT checklist (see link list at top right) with your submission. See author guidelines, under 'Reporting Guidelines'. Please confirm you have submitted this list.	Select response	NA

Data Availability

Data availability	Information included in the manuscript?	In which section is the information available?<br>(Reagents and Tools Table, Materials and Methods, Figures, Data Availability Section)
Have **primary datasets** been deposited according to the journal's guidelines (see 'Data Deposition' section) and the respective accession numbers provided in the Data Availability Section?	Select response	Data and Materials Availability
Were **human clinical and genomic datasets** deposited in a public access-controlled repository in accordance to ethical obligations to the patients and to the applicable consent agreement?	Select response	NA
Are **computational models** that are central and integral to a study available without restrictions in a machine-readable form? Were the relevant accession numbers or links provided?	Select response	Data and Materials Availability
If publicly available data were reused, provide the respective **data citations in the reference list**.	Select response	NA
